# Supplementary material for: Interfacility transfer of pregnant women using publicly funded emergency call centre-based ambulance services: a cross-sectional analysis of service logs from five states in India
Source: BMJ Open. 2017 Jun 9;7(6):e015077. doi: 10.1136/bmjopen-2016-015077 (PMC5734264; doi:10.1136/bmjopen-2016-015077)
Supplement: Author's manuscript [file bmjopen-2016-015077.draft_revisions.pdf]

BMJ Open

Inter-facility transfer of pregnant women in India using publicly-funded ‘108’ ambulances- Analysis of service logs

Journal:	*BMJ Open*
Manuscript ID	bmjopen-2016-015077
Article Type:	Research
Date Submitted by the Author:	07-Nov-2016
Complete List of Authors:	Singh, Samiksha; Indian Institute of Public Health-Hyderabad, PHFI<br>Doyle, Pat ; London School of Hygiene and Tropical Medicine, Faculty of Epidemiology and Population Health, Department of Non-communicable Disease Epidemiology<br>Campbell, Oona; London School of Hygiene and Tropical Medicine, Faculty of Epidemiology and Population Health, Department of Infectious Disease Epidemiology<br>Oakley, Laura; London School of Hygiene and Tropical Medicine, Faculty of Epidemiology and Population Health, Department of Non-communicable Disease Epidemiology<br>Rao, G.V. Ramana; GVK Emergency Management and Research Institute, Emergency Medicine Learning Center and Research<br>Murthy, GVS; Indian Institute of Public Health, Hyderabad, PHFI, Director; London School of Hygiene and Tropical Medicine, Faculty of Infectious and Tropical Diseases, Department of clinical research
**Primary Subject Heading**:	Public health
Secondary Subject Heading:	Health services research, Public health, Epidemiology, Obstetrics and gynaecology
Keywords:	Maternal medicine < OBSTETRICS, Ambulance, Inter-facility transfer, Obstetric emergency, Obstetric complication, Patient transport

SCHOLARONE™ Manuscripts

Title: Inter-facility transfer of pregnant women in India using publicly-funded '108' ambulances- Analysis of service logs

Short title: Inter-facility transfer of pregnant women in India using 108 ambulances

Samiksha Singh, Pat Doyle, Oona M R Campbell, Laura Oakley, GVR Rao, GVS Murthy

Samiksha Singh, (Corresponding author)

Assistant Professor,

Indian Institute of Public Health-Hyderabad, Public Health Foundation of India

Address- Plot#1 AVN Arcade, Amar Co-operative society, Kavuri Hills, Madhapur, Hyderabad, Telanagana, India - 201010; Email- samiksha.singh@iiphh.org; Phone- +91 8886 111 290

Pat Doyle

Professor,

Department of Non-communicable disease epidemiology, Faculty of Epidemiology and Population Health, London School of Hygiene and Tropical Medicine, London, UK

Oona M Campbell

Professor,

Department of Infectious Disease Epidemiology, Faculty of Epidemiology and Population Health, London School of Hygiene and Tropical Medicine, London, UK

For peer review only - http://bmjopen.bmj.com/site/about/guidelines.xhtml

Laura Oakley

Assistant Professor,

Department of Non-communicable disease epidemiology, Faculty of Epidemiology and Population Health, London School of Hygiene and Tropical Medicine, London, UK

GV Ramana Rao

Director,

Emergency Medicine Learning Center and Research, GVK-Emergency Management and Research Institute, Hyderabad, India

GVS Murthy

Professor,

Indian Institute of Public Health-Hyderabad, Public Health Foundation of India, Hyderabad, India and Department of clinical research, Faculty of Infectious and Tropical Diseases, London School of Hygiene and Tropical Medicine, London, UK

Word count: 4894

Key words: Ambulance, Inter-facility transfer, Obstetric emergency, Obstetric complication, Pregnant women, Patient transport, Access, Maternity Services, Travel time

For peer review only

ABSTRACT

Objective: To estimate the proportion of IFTs transported by '108' ambulances and to compare the characteristics of the IFTs and non-IFTs to understand the pattern of use of '108' services for pregnant women in India.

Design: A cross-sectional analysis of '108' ambulance records from five States for the period April 2013 to March 2014. Data were obtained from the GVK-EMRI database for the pregnant women, who called '108'.

Main outcomes: Proportion of all pregnancies and institutional deliveries in the population who were transported by '108', both overall and for IFT. Characteristics of the women transported; obstetric emergencies, the distances travelled, and the time taken for both IFT and non-IFT.

Results: The '108' ambulances transported 608,559 pregnant women, of whom 34,993 were transferred between facilities (5.8%) in the five States. We estimated that '108' transferred 16.5% of all pregnancies and 20.8% of institutional deliveries. Only 1.2% of all institutional deliveries in the population were transported by '108' for IFTs—lowest 0.6% in Gujarat and highest 3.0% in Himachal Pradesh. Of all '108' IFTs, only 8.4% had any pregnancy complication. For all States combined, on adjusted analysis, IFTs were more likely than non-IFTs to be for older and younger women or from urban areas, and less likely to be for women from high-priority districts, from backward or scheduled castes, or women below-the-poverty-line. Obstetric emergencies were more than twice as likely to be transferred between facilities as pregnant women without obstetric emergencies (OR=2.18, 95% CI, 2.09-2.27). There was considerable variation across States.

For peer review only - http://bmjopen.bmj.com/site/about/guidelines.xhtml

For peer review only

Conclusion: A very small proportion of institutional deliveries made use of the '108' ambulance for IFTs in India. The vast majority did not have any complication or emergency. The '108' service may need to consider strategies to prioritise the transfer of women with obstetric emergency and those requiring IFT, over uncomplicated non-IFT.

For peer review only

For peer review only - http://bmjopen.bmj.com/site/about/guidelines.xhtml

STRENGTHS & LIMITATIONS

- This study is the first to assess the role of the '108' ambulance service - the largest provider of the free emergency medical services in India – for inter-facility transfers (IFTs) of pregnant women.
- We estimated the number of institutional deliveries in five States of India and the proportion of mothers who were transported by '108' for IFTs and obstetric emergencies. This helped us assess the role of '108' ambulance services for pregnant women with emergencies.
- We assessed the proportion of obstetric emergencies and type of emergencies among pregnant women who were transported by '108' as IFTs and compared them with those who were transported by '108' as non-IFTs.
- The '108' database mostly did not record data for treatment given en-route, and doctors' notes on inter-facility transfers, thus we could not study these data.
- We had large proportions of missing information on social and economic status from two States. However they were appropriately dealt with in the analysis.

For peer review only

For peer review only - http://bmjopen.bmj.com/site/about/guidelines.xhtml

BACKGROUND

India had an estimated 83% women delivering in health facilities in 2013.[1] Despite this high proportion of institutional births, India had an estimated maternal mortality ratio (MMR) of 167/ 100,000 live births and an early neo-natal mortality rate of 28/1000 live births in 2013.[2] The country accounts for 17% (50,000) of global maternal deaths per year[3] and 26% (696,000) of global neonatal deaths.[4] Many maternal deaths occur during transit to health facilities.[5-7]

Inter-facility transfers for pregnant women are crucial, especially in resource poor countries where most peripheral health facilities provide only uncomplicated birthing or basic emergency obstetric care.[8,9] About 14% to 36% of women delivering in facilities are referred from lower to higher-level facilities.[10-13] As inter-facility transfer is more likely to be due to referral for high risk pregnancies or complications during pregnancy, childbirth or post-partum, it can play a pivotal role in reduction of maternal morbidity and mortality.[14, 15]

Ensuring an uneventful inter-facility transfer is part of good health care provision at the referring facility, and will reduce delays in access to appropriate health care (delay type-2) and delays in getting appropriate care after reaching a health facility (delay type-3).[16, 17] A good process will include prompt arrangement of transport, en-route stabilisation, communication with the referral facility to prepare them for the patient, and appropriate hand-over on arrival. Inter-facility transfer is thus a complex coordinated effort made by the referring health care provider, the en-route attendant, the receiver at the referral health facility, and the referral transport system.[16] Several successful interventions have been reported in resource-poor

For peer review only - http://bmjopen.bmj.com/site/about/guidelines.xhtml

countries to improve referral transportation, but only a few mention inter-facility transfers.[18, 19] Referral transport for inter-facility transfer may be a) an ambulance based at the referring facility, b) an ambulance called from a referral facility, c) an independent ambulance service, d) other subsidised public or commercial transport, or e) personal or commercial (non-subsidised) transport.[16, 20]

India, currently, does not have any structured inter-facility referral and transportation protocols. In India, most peripheral health facilities do not have functional ambulances of their own and if they do, they are not available round the clock.[14, 21, 22] Thus inter-facility transfer depends on other referral transport services, the majority using the free, public funded '108'/'102' call centre based ambulance services[19, 23, 24] and paid subsidised, commercial, or personal transport. The '108' service is free to all, while the '102' service is free for women in pregnancy and up to 6 weeks after delivery. Under Janani Shishu Suraksha Karyakaram, a government scheme, a cash payment can be made for the commercial transport for pregnant women and the newborn.[25] Assam has a dedicated fleet of ambulances for inter-facility transfers, operated under '108' services.[Source: Expert from GVK EMRI, Dr.GVR Rao] There are other services across States that, unlike '108', are basic patient transportation services with no en-route stabilisation care. These may also provide transport between facilities.[21, 26, 27] For example, Janani Express yojana (public private non-ambulance transport service), operating through call-centres within districts, transfers exclusively pregnant women and newborns in Madhya Pradesh and Odisha.[21, 26] In larger cities, some other ambulance services (free or paid) are also available.

For peer review only - http://bmjopen.bmj.com/site/about/guidelines.xhtml

There have been very few research studies on transportation for inter-facility transfers of pregnant or postpartum women in India, despite the large investments made in public referral transport. This study was conducted to investigate inter-facility transfers of pregnant women in India using '108' ambulance services. The objectives of the research were to i) estimate proportion of all institutional deliveries in the general population that were transported by '108' and the proportion of all institutional deliveries that were transferred between facilities by '108', ii) to estimate the proportion of all transfers of pregnant women by '108' that were transfers from one health facility to other (inter-facility transfers), and iii) to compare the characteristics of the inter-facility transfers and non-inter-facility transfers, across States.

METHODS

Context

The '108' ambulance service operates under a public private partnership. It operates 7361 ambulances, and transfers any medical emergency across 21 States and union territories (smaller less populated administrative units in India).[27] GVK-EMRI is the largest service provider for '108' and operates in 15 States and 2 union territories. In 2014-15, GVK-EMRI '108' ambulances transferred about 3.6 million pregnant women to health facilities, which were about two-fifths of all the transfers by GVK-EMRI '108'.[23] There is approximately one ambulance for every 100,000 population, and the ambulance should be well equipped and accompanied by an Emergency Medicine Technician (EMT). The EMT is trained to provide emergency care and basic life support in obstetrics. In cases of imminent childbirth, the EMT

For peer review only - http://bmjopen.bmj.com/site/about/guidelines.xhtml

should assist the delivery en-route and transfer the mother and child to the nearest health facility.[28] For inter-facility transfers, the EMT consults the '108' call-centre based medical officer and the referring health care provider to discuss indication of referral, stability of case to withstand travel, stabilising care required and place of referral [Source: Expert from GVK-EMRI).

Working definitions

Inter-facility transfer (IFT), for this study, was defined as any transfer of a pregnant woman from one health facility to another health facility on the advice of a health care provider, using a '108' ambulance. All other transfers of pregnant women to health facilities using '108' ambulances were defined as 'non-IFT'. These mostly included transfers from home to facility. An obstetric emergency was defined as any life threatening medical complication in women in pregnancy, labour or childbirth, or after (within 42 days of termination of pregnancy).

Study Design

This cross-sectional study analysed '108' ambulance records from five States for the period April 2013 to March 2014. Ethical approval for the study was obtained from Indian Institute of Public Health-Hyderabad and London School of Hygiene and Tropical Medicine.

Study population

Pregnant women who called '108' or for whom a relative or friend called on their behalf, between 1st April 2013 and 31st March 2014 in the five States in India where GVK-EMRI had

For peer review only - http://bmjopen.bmj.com/site/about/guidelines.xhtml

been fully functional for more than 3 years were included in this analysis (Andhra Pradesh, Chhattisgarh, Gujarat, Himachal Pradesh, and Telangana).

Obtaining data and data management

Formal permission to use the data was obtained from GVK-EMRI. Anonymised information on '108' calls from 1st April 2013 to 31st March 2014 was obtained from the GVK-EMRI emergency response centre database. Data were extracted onto Excel sheets, and converted to STATA 10.0 files. Data were inspected to assess consistency, range, and missing data. Any gross issue relating to the quality of records was noted, and records with improbable entries were excluded from analysis. Variables were recoded wherever needed. Variables of interest were: inter-facility transfer; age of pregnant woman; social caste; economic class (below or above poverty line); region (rural or urban); type of emergency; time of call; day of call; time taken by ambulance to reach the client; time taken to reach the health facility; and distance travelled. Districts within States were stratified into high priority districts and non-high priority districts. High priority districts are those identified by the Government of India as being in the lowest quartile of districts (or tribal districts) in each State with respect to maternal and child health indicators (including institutional delivery rate, maternal mortality and neonatal mortality rates among a total of 16 indicators).[29]

Analysis

We used information from the Census 2011 , District level household survey (DLHS) 2012-13,[30-32] and Annual health survey (AHS) 2012-13,[33] to estimate the number of pregnancies

For peer review only - http://bmjopen.bmj.com/site/about/guidelines.xhtml

and institutional deliveries in the population for the study States, as mentioned below. The numbers of pregnant women transported by '108' as recorded in the call-centre database were compared with these population estimates for each State.

For each State, the number of pregnancies in the study period was estimated as sum of estimated pregnancies in rural and urban population [population (rural) X crude birth rate (rural) X 1.1 X 1000] + [population (urban) X crude birth rate (urban) X 1.1 X 1000]. The population data were obtained from the 2011 census and the crude birth rates from the Sample Registration System 2013. The multiplier 1.1 is used to account for an estimated 10% of the pregnancies which may have ended in abortions or intra-uterine deaths.

The number of institutional deliveries in the study period was estimated as [Estimated number of pregnancies (rural) X institutional delivery rate (rural) X 100] + [Estimated number of pregnancies (urban) X institutional delivery rate (urban) X 100]; Institutional delivery rates include live births and still-births. The institutional delivery rate were obtained from DLHS-4 and AHS-2 surveys.

Information was analysed for all States combined and separately by State, comparing IFT and non-IFT. The characteristics of the women transported, distances travelled and the time taken by '108' ambulances, were described for both IFT and non-IFT journeys. The association between socio-demographic and clinical variables and the outcome (IFT vs no-IFT) was investigated using bivariate and multivariate logistic regression.

For peer review only - http://bmjopen.bmj.com/site/about/guidelines.xhtml

Social caste and economic class were missing in 55% and 95% of observations in the State of Chhattisgarh, and 14% and 22% in Himachal Pradesh. Given the nature of the variables we decided that it was likely that the data were missing not at random, and therefore multiple imputation was considered inappropriate. In order to investigate possible selection bias resulting from missing data, we performed a complete case analysis for each State and all States combined, supplemented by a series of sensitivity analyses. Our first sensitivity analysis involved running a model with and without States of Chhattisgarh and Himachal Pradesh. Presence or absence of these States did not substantially change the pattern of results. The second sensitivity analysis was performed by running a model with and without social caste and economic class for all States combined (total) and for individual States. The presence or absence of these variables did not substantially change the magnitude of the pattern of results for the total and for States (except Chhattisgarh). For our main analysis (all States combined) we thus included social caste and economic class variables and excluded any data from Chhattisgarh. Models for individual States (except Chhattisgarh) also include social caste and economic class.

RESULTS

Study Populations

The study States had population sizes ranging from 6 million to 60 million and had different social compositions (Table-1). The percentage of rural population ranged from 61% in Telangana to 90% in Himachal Pradesh while scheduled castes and tribes, together, ranged from 22% in Andhra Pradesh and Gujarat to 43% in Chhattisgarh. The crude birth rate was

For peer review only - http://bmjopen.bmj.com/site/about/guidelines.xhtml

lowest in Himachal Pradesh (1.6%) and highest in Chhattisgarh (2.5%). The States also varied in the institutional delivery rates from 39.5% in Chhattisgarh to 94.1% in Telangana, and in their MMRs from 244 in Chhattisgarh to 92 per 100,000 live births in Telangana and Andhra Pradesh. On an average '108' ambulance is sanctioned for approximately 100,000 population in all States except Himachal Pradesh where one ambulance caters to about 40,000 population. Himachal Pradesh is mostly hilly and has sparse population distribution and '108' ambulances are stationed based on geographic regions.

Table 1: Demographic characteristics of the study States

	Andhra Pradesh	Chhattisgarh	Gujarat	Himachal Pradesh	Telangana
Total Population*	49,386,799	25,540,196	60,383,628	6,856,509	35,193,978
Rural/tribal	70.4%	76.8%	57.4%	90.0%	61.3%
Urban	29.6%	23.2%	42.6%	10.0%	38.7%
Scheduled caste*	17.1%	12.8%	6.8%	25.2%	15.4%
Scheduled tribe*	5.3%	30.6%	14.8%	5.7%	9.3%
Crude birth rate per 1000 population**	17.5	24.5	21.1	16.2	17.5
Institutional delivery rate***	88.5%	39.5%	78.1%	77.8%	94.1%
MMR(2011-13)**	92	244	112	Not available	92
NMR(2012-13)**	25	31	26	25	25
No. of Ambulances under '108'[#]	468	240	506	171	334
Region	Tribal pockets	Large Tribal; hilly pockets	Tribal pockets	Majority hilly	Tribal pockets

Source- *Census 2011; ** Sample Registration System 2013- Separate data for Telangana and Andhra Pradesh not available; *** DLHS (2012-13) / AHS (2011-12); [#] GVK-EMRI annual report for period April 2013- March 2014. [$]In Chhattisgarh '102' ambulance service took over from October 2013 – March 2014.

Description of 108 calls relating to pregnancy

Across the five States, '108' call centres received 646,656 calls for pregnancy-related transfers, 6.2% of which were for IFT. Among IFT calls, an ambulance was not assigned to 1.2%, and an

For peer review only - http://bmjopen.bmj.com/site/about/guidelines.xhtml

ambulance was assigned but not used by 6.0% of callers. Among non-IFT callers these proportions were 1.4% and 3.9% respectively. A total of 608,559 pregnant women were transported using '108', and of these journeys 5.8% were for IFT.

Estimated proportions of pregnancies, institutional deliveries and IFTs transported by '108'

Table-2 presents estimates of the number of pregnant women, obstetric emergencies, institutional deliveries and inter-facility transfers in the general populations, and the proportion of these transported using '108' ambulances in the five states, and overall. The 608,559 pregnant women transferred comprised 16.5% of all estimated pregnancies, and 20.8% of all estimated institutional deliveries for the study States combined.

Table 2: Estimated proportion of pregnant women transported to hospitals by '108' from April 2013 to March 2014

	Total	Andhra Pradesh	Chhattisgarh [$]	Gujarat	Himachal Pradesh	Telangana
Estimated number of pregnancies in the population [a]	3,811,920	950,696	678,217	1,387,021	121,638	673,508
Estimated number of institutional deliveries in the population [b]	2,921,674	842,109	267,896	1,083,264	94,634	633,771
Proportion of all pregnancies transported by '108' [c]	16.5%	15.5%	9.0%	19.5%	20.5%	15.7%
Proportion of all institutional deliveries transported by '108' [d]	20.8%	17.5%	22.7%	24.9%	26.3%	16.6%
Proportion of all institutional deliveries that were transported by '108' for inter-facility transfers [e]	1.2%	1.7%	0.7%	0.6%	3.0%	1.4%
Proportion of institutional deliveries that were transported by '108' for obstetric emergencies [f]	1.0%	0.6%	1.0%	1.4%	2.4%	0.6%

[a] [population (rural) X crude birth rate (rural) X 1.1 X 1000] + [population (urban) X crude birth rate (urban) X 1.1 X 1000]; Using Census populations and crude birth rates from Sample Registration Survey in respective States

[b] [Estimated number of pregnancies (rural) X institutional delivery rate (rural) X 100] + [Estimated number of pregnancies (urban) X institutional delivery rate (urban) X 100]; Institutional delivery rates include live births and still births. Using institutional delivery rates from District Level Household Survey / Annual Household Survey in respective States

[c] Number of pregnant women transported by '108'/Estimated number of pregnancies in the population([a])

[d] Number of pregnant women transported by '108'/Estimated number of institutional deliveries in the population([b])

[e] Number of pregnant women transported between facilities (IFTs) by '108'/ estimated number of institutional deliveries in the population([b])

For peer review only - http://bmjopen.bmj.com/site/about/guidelines.xhtml

[f] Number of pregnant women transported by '108' for obstetric emergency / estimated number of institutional deliveries in the population([b])
[$]In Chhattisgarh '102' ambulance service took over from '108' service from October 2013 to March 2014. Thus number of beneficiaries of '108' and proportions may be underestimation of utilization for annual estimates.

Of the estimated institutional deliveries, '108' transported only 1.2% (34,993) women between facilities across States. This proportion was highest in Himachal Pradesh (3.0%) followed by Andhra Pradesh (1.7%), Telangana (1.4%), Chhattisgarh (0.7%) and lowest in Gujarat (0.6%). Only 1.0% (28,448) of all institutional deliveries were transported by '108' for obstetric emergencies—highest 2.4% in Himachal Pradesh and lowest 0.6% in Andhra Pradesh and Telangana (Table-2).

Characteristics of pregnant women transferred by '108' ambulances

The pregnant women who were transferred by '108' belonged mostly to lower social and economic sections- rural or tribal areas (84.6%) or scheduled castes or tribes (64.9%) and below-the-poverty-line status (76.6%). Two hundred and forty two women (4 per 100,000) died before the ambulance reached the pick-up site. The proportion of pregnant women who died before arrival of '108' ambulance was higher in Chhattisgarh (0.15%) compared to other States.

Of the pregnant women transferred by '108' ambulances, 34,993 (5.8%) had an IFT (Table-3). The proportion of IFTs among women transported by '108' was highest in Himachal Pradesh (11.3%) followed by Andhra Pradesh (9.9%), Telangana (8.7%), Chhattisgarh (3.2%) and Gujarat (2.4%).

For peer review only - http://bmjopen.bmj.com/site/about/guidelines.xhtml

Overall, IFTs were made up of a higher proportion of younger women, women from backward caste, belonging to below-the-poverty-line and from urban areas compared to non-IFTs. A Similar pattern was observed across all the States except in Himachal Pradesh where IFTs had lower proportion of women from below-the-poverty-line strata compared to non-IFTs (Table-3). Delivery en-route or in the ambulance was less likely among IFTs compared to non-IFTs across all States.

For peer review only

For peer review only - http://bmjopen.bmj.com/site/about/guidelines.xhtml

Table 3: Demographic characteristics of users of '108' ambulance service for IFT and non-IFT in different States (April 2013 to March 2014)

	Total		Andhra Pradesh		Chhattisgarh		Gujarat		Himachal Pradesh		Telangana	
	IFT (N=34,993) [5.8%]	Non-IFT (N=573,566)	IFT (N=14,574) [9.9%]	Non-IFT (N=132,800)	IFT (N=1,929) [3.2%]	Non-IFT (N=58,881)	IFT (N=6,562) [2.4%]	Non-IFT (N=263,509)	IFT (N=2,809) [11.3%]	Non-IFT (N=22,114)	IFT (N=9,119) [8.7%]	Non-IFT (N=96,262)
Age group (years)												
<u><</u>19	4.1	3.4	5.0	4.8	7.1	5.8	2.3	2.0	4.4	5.7	3.5	3.6
20 – 24	58.7	56.5	64.9	65.7	54.9	56.4	52.0	51.9	48.3	51.6	58.6	58.9
25 – 30	28.1	30.6	24.0	24.9	25.0	27.9	30.1	33.2	34.5	32.0	31.4	32.2
30 – 34	6.5	7.2	4.2	3.3	8.9	7.2	11.3	9.9	9.4	8.0	4.8	4.1
<u>></u>35	2.6	2.3	1.9	1.2	4.1	2.7	4.3	3.0	3.4	2.7	1.7	1.2
Total non-missing	(32,538) 100	(551,568) 100	(13,105) 100	(120,155) 100	(1,914) 100	(58,591) 100	(6,562) 100	(263,508) 100	(2,779) 100	(21,694) 100	(8,178) 100	(87,620) 100
missing	(2,455) 7.0	(21,988) 3.8	(1,469) 10.1	(12,645) 9.5	(15) 0.8	(290) 0.5	(0) 0	(1) 0	(30) 1.1	(420) 1.9	(941) 10.3	(8,642) 9.0
Social caste												
General caste	12.2	11.3	10.8	10.9	3.7	3.5	14.8	12.8	37.3	38.2	7.1	5.0
Other backward	40.0	38.5	41.7	44.1	48.5	43.8	38.3	35.0	17.4	11.5	43.4	43.5
Scheduled caste	29.1	22.1	34.7	34.1	14.3	14.7	11.2	11.1	37.2	42.8	32.2	34.2
Scheduled tribe	18.7	28.2	12.8	10.9	33.6	38.1	35.8	41.2	8.1	7.6	17.3	17.3
Total non-missing	(32,800) 100	(535,328) 100	(14,400) 100	(131,924) 100	(652) 100	(27,427) 100	(6,551) 100	(263,204) 100	(2,201) 100	(17,148) 100	(8,996) 100	(95,625) 100
DK/ missing	(2,193) 6.3	(38,238) 6.7	(174) 1.2	(876) 0.7	(1,277) 66.2	(31,454) 53.4	(11) 0.2	(305) 0.1	(608) 21.6	(4,966) 22.5	(123) 1.3	(637) 0.7
Economic class												
BPL	87.7	75.9	99.7	99.6	96.7	97.0	54.2	55.9	62.7	67.0	99.5	99.5
Others	12.3	24.1	0.3	0.4	3.3	3.0	45.7	44.1	37.3	33.0	0.5	0.5
Total non-missing	(32,296) 100	(511,447) 100	(14,357) 100	(131,391) 100	(61) 100	(2824) 100	(6,540) 100	(262,991) 100	(2,395) 100	(19,041) 100	(8,943) 100	(95,200) 100
DK/ missing	(2,697) 7.7	(62,119) 10.8	(217) 1.5	(1,409) 1.1	(1,868) 96.8	(56,057) 95.2	(22) 0.3	(518) 0.2	(414) 14.7	(3,073) 13.9	(176) 1.9	(1,062) 1.1
Area												
Rural/tribal	68.7	85.6	67.4	79.5	92.1	92.7	62.8	89.3	75.9	95.2	67.6	77.8
Urban	31.3	14.4	32.6	20.5	7.9	7.3	37.2	10.7	24.1	4.8	32.4	22.2
Total non-missing	(34,910) 100	(570,042) 100	(14,555) 100	(132,510) 100	(1,929) 100	(58,881) 100	(6,562) 100	(263,509) 100	(2,770) 100	(18,952) 100	(9,094) 100	(96,190) 100
Missing	(78) 0.2	(3,524) 0.6	(19) 0.1	(290) 0.2	(0) 0	(0) 0	(0) 0	(0) 0	(39) 1.4	(3,162) 14.3	(25) 0.3	(72) 0.1
Died before ambulance reached	0.07	0.04	0.04	0.02	0.36	0.14	0.02	0.03	(0) 0	0.01	0.02	0.02
Delivery en-route												
At pick up site	0.3	1.4	0.2	0.8	1.2	2.8	0.5	1.7	0.2	1.7	0.2	0.7
In ambulance	0.6	1.0	0.4	0.7	0.8	1.5	0.6	0.9	1.7	3.5	0.4	0.7

IFT= Inter-facility transfer ; non-IFT = non-inter-facility transfer

For peer review only - http://bmjopen.bmj.com/site/about/guidelines.xhtml

Obstetric emergencies among women transferred by '108' ambulance

The majority of transfers by '108' ambulances were for normal labour. By State, between 2.7% and 9.3% of transferred pregnant women had an obstetric emergency; the overall total was 4.8%. The IFTs (8.4%) had higher proportion of pregnant women who had an obstetric emergency compared to non-IFTs (4.4%) (Table-4). Himachal Pradesh had highest proportion of obstetric emergencies transferred for both IFT (21.0%) and non-IFT (7.8%). Andhra Pradesh was the lowest proportion of obstetric emergencies transferred for IFT (5.4%) and non-IFT (3.1%).

The most common obstetric emergencies were 'abnormal presentation of foetus' and 'bleeding in pregnancy' in Telangana, Andhra Pradesh and Gujarat; and 'bleeding in pregnancy' and 'medical conditions complicating pregnancy' in Himachal Pradesh and Chhattisgarh. IFTs had higher proportions of 'bleeding in pregnancy' cases across all the States and 'medical conditions complicating pregnancy' in Himachal Pradesh and Chhattisgarh (Table-4). In Telangana and Andhra Pradesh, some IFTs (0.5% and 0.2%) and non-IFTs (8.43% and 0.53%) were for antenatal women requiring check-ups at higher institutions (data not shown).

Destination facilities

About 86% of all transfers were to government or government supported hospitals. This figure was highest in Himachal Pradesh (97%) and lowest in Andhra Pradesh where around 82% of women were transferred to government hospitals (table-4). In Chhattisgarh transfers to government or supported hospitals were 9.2% more among non-IFTs than IFTs (96.8% and

For peer review only - http://bmjopen.bmj.com/site/about/guidelines.xhtml

87.6%). The proportions of transfers to government or government supported hospital were similar in IFTs and non-IFTs overall and in other States. However, with type of government facility, across the States, half of the IFTs were to district level secondary or tertiary hospitals while non-IFTs were to sub-district Area or Civil hospitals or CHCs. Gujarat reported highest percentage of transfers to middle level facilities. Himachal Pradesh had more transfers to Civil-hospitals or higher (Table-4).

Distance and time travelled by 108 ambulance for pregnant women

The median distances travelled and time taken by '108' ambulances to transfer pregnant women are shown in Table-4. Ambulances travelled less than 4 kilometres to reach half of the pregnant women for IFTs across four States but up to 10 kilometres in Andhra Pradesh. For non-IFTs, ambulances travelled farther (between 9 and 12 kilometres) to reach half of the pregnant women. Among IFTs median distances from pick-up site to destination facility were between 16 kilometres in Chhattisgarh to 32 kilometres in Himachal Pradesh, but nearer in non-IFTs (between 8 and 32).

The median time taken was corresponding to for the ambulance to travel to the pick-up site for IFTs was between 10 minutes and 15 minutes for four States but longer (24 minutes) in Andhra Pradesh. The median time taken for travel from the referring to the referral facility was less than 40 minutes in Andhra Pradesh, Chhattisgarh and Gujarat but longer for Telangana (48 minutes) and Himachal Pradesh (83 minutes). For non-IFT, these times were about 30 minutes each, except for Himachal Pradesh where travel times were longer (Table-4).

For peer review only - http://bmjopen.bmj.com/site/about/guidelines.xhtml

Table 4: Characteristics of transfers for IFT and non-IFT by state in different States (April 2013 to March 2014)

	Total		Andhra Pradesh		Chhattisgarh		Gujarat		Himachal Pradesh		Telangana	
	IFT (N=34,993)	Non-IFT (N=573,566)	IFT (N=14,574)	Non-IFT (N=132,800)	IFT (N=1,929)	Non-IFT (N=58,881)	IFT (N=6,562)	Non-IFT (N=263,509)	IFT (N=2,809)	Non-IFT (N=22,114)	IFT (N=9,119)	Non-IFT (N=96,262)
Obstetric emergency, %	8.4	4.4	5.4	3.1	18.0	3.9	10.4	5.5	21.0	7.8	5.8	3.2
Abnormal presentation	1.5	2.0	1.0	1.0	1.3	1.2	3.2	3.1	0.7	0.4	1.3	1.1
Bleeding in pregnancy	3.5	0.9	2.0	0.6	1.6	0.4	4.5	1.0	14.1	5.6	2.1	0.6
Eclampsia/ convulsion	0.7	0.1	0.6	0.2	0.7	0.1	0.6	0.1	1.1	0.1	0.8	0.2
Foetal loss	0.2	0.1	0.1	0.1	0.6	0.4	0.1	0.02	0.4	0.1	0.1	0.1
Medical condition	1.8	0.7	0.9	0.6	13.6	1.7	1.0	0.4	3.8	1.1	0.8	0.8
Previous caesarian	0.4	0.4	0.5	0.3	0.3	0.2	0.5	0.5	0.3	0.3	0.4	0.3
Precious pregnancy	0.3	0.3	0.2	0.2	0.1	0.1	0.5	0.4	0.5	0.2	0.3	0.2
Other emergency, %	3.7	1.8	4.1	2.8	8.7	1.2	2.3	1.0	0	0	4.2	3.5
Destination hospital, %												
Government	82.0	79.6	82.1	81.7	87.6	96.8	70.2	72.0	96.0	97.6	85.3	84.1
District Secondary or tertiary hospital	53.1	19.7	54.2	29.4	64.3	28.5	30.1	7.3	86.8	45.5	55.5	31.7
ESI hospital	0.1	0.1	0	0	0	0	0	0	4(0.1	1.2	0.2	0.3
Area / Civil hospital	19.8	13.4	18.1	21.1	0.6	0.8	31.5	8.3	7.7	27.3	21.3	22.5
CHC	7.6	34.6	8.3	19.7	20.5	57.4	7.4	43.6	0.9	20.4	6.3	16.2
PHC / UHC	1.1	9.0	1.2	11.3	1.9	9.3	0.2	7.0	0.3	3.2	1.9	13.1
SHC	0	0.1	0	0	0.3	0.9	0	0	0	0	0	0
Information Missing	0.4	2.8	0.3	0	0	0	0.9	5.6	0	0	0.2	0.2
Government supported	3.4	7.7	0	0	0	0	16.9	15.9	0	0	0	0
Private	14.5	12.8	17.9	18.3	12.4	3.2	12.9	12.1	4.0	2.4	14.7	15.9
Total non-missing	(31,911) 100	(531,392) 100	(12,693) 12.9	(116,028) 100	(1,784) 100	(54,093) 100	(6,442) 100	(256,898) 100	(2,777) 100	(21,449) 100	(8,215) 100	(82,924) 100
Missing	(3082) 8.8	(42,174) 7.4	(1,881) 12.9	(16,772) 12.6	(145) 7.5	(4,788) 8.1	(120) 1.8	(6,611) 2.5	(32) 1.1	(665) 3.0	(904) 9.9	(13,338) 13.9
To different district	32.1	28.5	29.8	28.8	62.5	54.3	17.7	22.0	31.6	10.1	39.7	34.3
To different mandal	86.8	55.9	89.5	75.7	83.2	68.5	74.1	33.0	96.3	93.5	89.5	74.9
Distance call to pick-up site* Km Median (IQR)	2(1-14)	12(6-18)	10(3-18)	12(6-19)	4(1-6)	9(2-17)	1(1-10)	12(7-18)	1(1-1)	11(6-18)	3(1-15)	12(6-20)
Distance pick-up site to hospital,* km Median (IQR)	28(18-40)	15(8-23)	21(12-31)	17(10-26)	16(5-30)	8(3-15)	24(14-37)	14(8-21)	32(21-48)	12(6-20)	29(19-43)	17(10-28)
Time call to pick-up site,* minutes ; Median (IQR)	11(7-28)	25(16-37)	24(12-37)	27(17-40)	15(7-33)	27(17-39)	10(7-19)	24(16-34)	12(8-27)	35(22-56)	12 (7-29)	26(16-39)
Time pick-up site to hosp,* minutes ; Median(IQR)	49(32-70)	26(16-40)	37(23-55)	32(20-46)	40(26-64)	21(13-32)	40(26-60)	23(14-34)	83(57-116)	43(28-65)	48 (33-70)	31(20-48)
Time call to hospital,* minutes ; Median (IQR)	77(56-102)	63(46-84)	75(55-97)	72(54-93)	70(50-97)	56(39-77)	64(47-86)	56(42-74)	105(72-143)	80(54-121)	77 (57-103)	70(52-93)

IFT= Inter-facilty transfer; IQR=Interquartile range; *N varies- excludes deliveries by EMT that were not transported or missing value

For peer review only - http://bmjopen.bmj.com/site/about/guidelines.xhtml

Time of day and day of week when pregnant women were transferred

The transfers by '108' were more frequent during 9:00 hours and 15:00 hours, peaking around 12:00 hours with a second smaller peak at around 22:00 hours, in all the States (Figure-1). Telangana, Himachal Pradesh and Gujarat had slightly higher proportions of transfers on Mondays, and Chhattisgarh on Thursdays. Across all the States, it was evident that a higher proportion of IFTs compared to non-IFTs were occurring between 10:00 hours to 23:00 hours.

Figure-1 here

Determinants of Inter-facility transfer

Overall, women with obstetric emergencies transported by 108 were roughly twice as likely to have an IFT as women with no obstetric emergency (crude OR 2.25, 95% CI 2.16-2.34) (Table-5). In the adjusted analysis (excluding Chhattisgarh), obstetric emergencies had 1.95 (95% CI, 1.83–2.06) times higher odds of having IFT compared to non-emergencies (Table-5). Women from urban areas were twice as likely to have IFTs as women in rural areas (AOR 2.34, 95% CI 2.26 – 2.40). There was no evidence of an independent effect of high-priority districts on IFT. Overall, there was evidence of a 'J' shaped trend with maternal age. IFTs were marginally more likely in women less than 25, with a trend of increasing odds in women aged 30 or more, compared to women aged 25-30 years (AOR 1.01, 1.04, 1, 1.21, 1.43 respectively for age groups $\leq$19, 20-24, 25-30, 30-34 and $\geq$35 years). Although the effects were small, women from backward castes (AOR 0.95, 95% C.I. 0.92-0.99), scheduled castes (AOR 0.94, 95% C.I. 0.90-0.98), and scheduled tribes (AOR 0.98, 95% C.I. 0.95-1.00) were less likely to have IFT than women from general

For peer review only - http://bmjopen.bmj.com/site/about/guidelines.xhtml

castes, after adjustment. Women from below the poverty line (AOR 0.92, 0.88-0.96) were less likely to have IFTs compared to women above the poverty line.

In respect to individual State models, the association between obstetric emergencies and IFT was strongest for Chhattisgarh (AOR 5.32, 95% CI 4.70-6.02) followed by Himachal Pradesh (AOR 3.03, 95% CI 2.65-3.47). The effect for urban women to be IFTs was highest in Himachal Pradesh (AOR 5.76, 95% CI 5.03 – 6.59) and Gujarat (AOR 4.75, 95% CI 4.50 – 5.01). Women from high priority districts had a slightly higher odds of being an IFT in the States of Chhattisgarh (AOR 1.25, 95% C.I. 1.11 – 1.41) and Andhra Pradesh (AOR 1.09, 95% C.I. 1.05 – 1.13), with no observed effect in other States. The 'J' shaped pattern for age was more evident in States of Chhattisgarh and Gujarat while no pattern was observed in Himachal Pradesh. In Telangana women from all three socially disadvantaged castes (AORs between 0.74 and 0.82) and in Andhra Pradesh women from backward caste (AOR 0.93, 95% C.I. 0.87-0.99) were less likely to have IFTs. Conversely, in Himachal Pradesh women from backward castes (AOR 1.80, 95% C.I. 1.55-2.10) and scheduled tribes (AOR 1.31, 95% C.I. 0.77-0.95) and in Andhra Pradesh women from scheduled tribe (AOR 1.22, 95% C.I. 1.13-1.31) were more likely to have IFTs. Women from below the poverty line in Himachal Pradesh (AOR 0.85, 95% C.I. 0.77-0.95) and Gujarat (AOR 0.95, 95% C.I. 0.90-1.00) were less likely to have IFT. Although evidence was weak, women from below the poverty line in Telangana (AOR 1.34, 95% C.I. 0.95-1.88) and Andhra Pradesh (AOR 1.40, 95% C.I. 1.00-1.96) were more likely to have IFTs (Table-5).

For peer review only - http://bmjopen.bmj.com/site/about/guidelines.xhtml

For peer review only

Table-5 Determinants of IFT across States (Logistic regression analysis showing Odds ratio (95% C.I.))

	Total*	Andhra Pradesh	Chhattisgarh	Gujarat	Himachal Pradesh	Telangana
Bivariate logistic regression: OR (95% CI)						
Obstetric emergency	2.25 (2.16 – 2.34)	1.81 (1.67 – 1.95)	5.39 (4.76 – 6.09)	2.01 (1.85 – 2.17)	3.15 (2.84 – 3.49)	1.90 (1.73 – 2.09)
Urban area	2.29 (2.23 – 2.34)	1.87 (1.80 – 1.94)	1.09 (0.92 – 1.29)	4.93 (4.69 – 5.20)	6.34 (5.68 – 7.07)	1.68 (1.60 – 1.76)
High priority district	0.96 (0.94 – 0.98)	1.10 (1.06 – 1.14)	1.28 (1.13 – 1.44)	0.69 (0.65 – 0.73)	0.92 (0.83 – 1.01)	0.95 (0.90 – 1.00)
Age group (years)						
≤19	1.06 (1.00 – 1.13)	1.08 (0.98 – 1.18)	1.37 (1.13 – 1.66)	1.28 (1.08 – 1.51)	0.70 (0.57 – 0.86)	1.01 (0.88 – 1.14)
20 – 24	1.03 (1.00 - 1.05)	1.02 (0.98 – 1.07)	1.09 (0.97 – 1.21)	1.10 (1.04 – 1.16)	0.87 (0.79 – 0.95)	1.02 (0.97 – 1.07)
25 – 30	1	1	1	1	1	1
30 – 34	1.24 (1.18 - 1.30)	1.29 (1.17 – 1.42)	1.38 (1.16 – 1.65)	1.25 (1.15 – 1.36)	1.08 (0.93 – 1.25)	1.21 (1.08 – 1.35)
≥35	1.52 (1.41 – 1.64)	1.62 (1.41 – 1.86)	1.68 (1.31 – 2.15)	1.58 (1.39 – 1.80)	1.16 (0.93 – 1.46)	1.47 (1.23 – 1.76)
Social caste						
General caste	1	1	1	1	1	1
Backward caste	0.92 (0.89 – 0.96)	0.95 (0.90 – 1.01)	1.04 (0.68 – 1.58)	0.94 (0.87 – 1.02)	1.55 (1.36 – 1.77)	0.71 (0.65 – 0.77)
Scheduled caste	0.91 (0.87 – 0.94)	1.02 (0.97 – 1.09)	0.91 (0.58 – 1.44)	0.87 (0.79 – 0.96)	0.89 (0.80 – 0.99)	0.67 (0.61 – 0.73)
Scheduled tribe	0.90 (0.87 – 0.94)	1.18 (1.11 – 1.28)	0.83 (0.54 – 1.27)	0.75 (0.69 – 0.81)	1.10 (0.93 – 1.31)	0.71 (0.65 – 0.78)
Below poverty line	0.92 (0.88 – 0.96)	1.45 (1.04 – 2.01)	0.92 (0.22 – 3.80)	0.93 (0.89 – 0.98)	0.83 (0.76 – 0.90)	1.03 (0.75 – 1.41)
Multivariate logistic regression model: AOR (95% CI)**						
Obstetric emergency**	1.96 (1.83 – 2.06)	1.80 (1.65 – 1.95)	5.32 (4.70 – 6.02)	1.89 (1.74 – 2.05)	3.03 (2.65 – 3.47)	1.79 (1.61 – 1.98)
Urban area	2.35 (2.26 – 2.41)	1.86 (1.79 – 1.94)	1.09 (0.92 – 1.29)	4.75 (4.50 – 5.01)	5.76 (5.03 – 6.59)	1.64 (1.56 – 1.73)
High priority district	0.98 (0.95 – 1.00)	1.09 (1.05 – 1.13)	1.25 (1.11 – 1.41)	0.90 (0.85 – 0.96)	1.17 (1.03 – 1.33)	1.00 (0.95 – 1.06)
Age group (years)						
≤19	1.01 (0.94 – 1.06)	1.01 (0.92 – 1.11)	1.36 (1.12 – 1.65)	1.25 (1.06 – 1.49)	0.82 (0.65 – 1.05)	0.91 (0.80 – 1.05)
20 – 24	1.04 (0.98 – 1.06)	1.03 (0.99 – 1.08)	1.11 (1.00 – 1.24)	1.12 (1.06 – 1.18)	0.92 (0.82 – 1.03)	1.02 (0.97 – 1.07)
25 – 30	1	1	1	1	1	1
30 – 34	1.21 (1.14 – 1.27)	1.26 (1.14 – 1.39)	1.33 (1.11 – 1.59)	1.21 (1.11 – 1.32)	0.94 (0.77 – 1.14)	1.19 (1.07 – 1.34)
≥35	1.43 (1.35 – 1.56)	1.56 (1.36 – 1.80)	1.52 (1.19 – 1.95)	1.44 (1.27 – 1.64)	0.96 (0.72 – 1.30)	1.43 (1.19 – 1.71)
Social caste						
General caste	1	1	NA[#]	1	1	1
Backward caste	0.95 (0.92 – 0.99)	0.93 (0.87 – 0.99)	NA[#]	1.02 (0.94 – 1.10)	1.80 (1.55 – 2.10)	0.79 (0.71 – 0.87)

For peer review only - http://bmjopen.bmj.com/site/about/guidelines.xhtml

Scheduled caste	0.94 (0.90 – 0.98)	1.02 (0.95 – 1.08)	NA[#]	0.99 (0.90 – 1.09)	1.01 (0.90 – 1.14)	0.74 (0.61 – 0.73)
Scheduled tribe	0.98 (0.96 – 1.00)	1.22 (1.13 – 1.31)	NA[#]	0.94 (0.87 – 1.01)	1.31 (1.08 – 1.61)	0.82 (0.67 – 0.82)
Below poverty line	0.92 (0.88 – 0.96)	1.40 (1.00 – 1.96)	NA[#]	0.95 (0.90 – 1.00)	0.85 (0.77 – 0.95)	1.34 (0.95 – 1.88)
Pseudo R2	0.076	0.014	0.033	0.054	0.085	0.010

*adjusted for i.State and does not include State of Chhattisgarh; ** Adjusted for obstetric emergency, urban area, high priority district and age group, as appropriate; # for Chhattisgarh social caste and below poverty line were not included.

DISCUSSION

This is the first study assessing inter-facility transfers for pregnant women using the '108' ambulance service—the largest provider of the emergency medical services in India. We discuss findings with respect to patterns of use and the existing health system.

We estimated that '108' transferred around one fifth of all pregnancies and institutional deliveries in the five States. However, only 1.2% of all institutional deliveries were transported by '108' for inter-facility transfers. Only 1% of all institutional deliveries were transported by '108' for obstetric emergencies. The findings suggest that the '108' service is not a preferred choice for transport to a higher-level facility, or for obstetric emergencies. The proportion of IFTs among all institutional deliveries will depend on the pattern of use of level of health care, referral practices and the availability of transport for between facility transfers.

Roughly one-half of the non-IFTs went to peripheral birthing centres or basic EmOC centres. A systematic review from India (including most studies from public health facilities) found that between 14% and 36% of all pregnancies were referred from nurse-run delivery or basic EmOC centres, and a further 2% to 7% were referred from doctor- run basic EmOC centres for complications or emergencies.[13] Assuming the above pattern of use of health facilities we

For peer review only - http://bmjopen.bmj.com/site/about/guidelines.xhtml

estimate that between 7% and 19% of all non-IFTs by ‘108’ may require further referral to higher facility. In addition, some women who went to their first facility by other means of transport may be referred further using ‘108’. In our analysis, of all the transfers by ‘108’ only 5.8% were between facilities—lowest 2.4% in Gujarat and highest 11.3% in Himachal Pradesh. The proportion of IFT was higher in States of Karnataka and Tamil Nadu (12.8% and 35.7%) among ‘108’ users in 2013-14.[24] It appears that there is potential for increasing use of ‘108’ for IFTs in the study States.

A study conducted by NHSRC in Andhra Pradesh in 2009, found that none of the pregnant women who used ‘108’, was referred from a facility.[34] Some women did not prefer to wait for ‘108’ if they perceived any emergency.[34] A maternal death review in Uttar Pradesh in 2010 found that only 5 of the 32 mothers who were transferred between facilities used an ambulance.[6] However, for other public transportation schemes, it was found that a high proportion (two-thirds) of all the inter-facility referrals in a study from Madhya Pradesh used Janani Express service (non-ambulance) while others used personal transport, taxis, autorikshaws or public transport.[10]

Although IFTs in our study were twice as likely to transport pregnant women who had any obstetric emergency compared to non-IFTs, there was a very large proportion of IFTs with no emergency or complication (92%). One of the ‘108’ doctors, during discussion to understand IFT processes, mentioned that on many occasions they were not convinced of the need for IFT. However on insistence of the referring staff, the ‘108’ doctor approved transport for IFT

For peer review only - http://bmjopen.bmj.com/site/about/guidelines.xhtml

(personal communication). Often the referral was done because there was no doctor on duty or other resources were not available, as was also found in Madhya Pradesh.[10] These findings raise the question of whether such sophisticated ambulances are needed to transfer non-emergency IFTs, making the ambulances unavailable for other emergencies.

In our study, IFTs had a higher overall proportion of women from disadvantaged social castes, below-the-poverty-line and urban areas compared to non-IFTs. Greater proportions of urban women deliver in health facilities,[30-32] and thus have a higher probability of early detection of complications and referral to a higher facility. Studies have also shown that higher proportions of women who use publically financed transportation schemes (‘108’/‘102’/Janani Express Yojana) belong to historically disadvantaged and backward social caste, below-the-poverty-line, are less educated and mostly from rural geographical areas compared to non-users.[21, 34-36]

Calls to ‘108’ were more frequent between 8:00 to 14:00 hours, as also reported by others.[36, 37] A lag of about one hour was observed between peak hours of call for non-IFTs and IFTs. It is plausible that women first reached a facility from home using ‘108’ (non-IFT) and, after initial assessment, some were referred to another facility using the ‘108’ service. The lag time was required to determine need of referral, provide initial care and arrange notes. A higher proportion of IFTs immediately after 10:00 hours could also be due to the fact that medical officers usually arrive on duty at 9:00 hours to conduct rounds, and would refer the sick cases admitted on the previous night.

For peer review only - http://bmjopen.bmj.com/site/about/guidelines.xhtml

The median time from call to '108' and reaching the destination facility was similar for non-IFT, and IFT, and it ranged from 1 hour to 1.3 hour. A study of 57 maternal death reviews from Uttar Pradesh found much longer times; the mean time taken to arrange transport and travel from home to facility was about 4 hours, and transport from one facility to another was about 10 hours.[6] Although the '108' service has a mandate to inform the destination facility before arrival to reduce delays in treatment, this is not practiced as the list of contact points is not provided to EMTs.[Source: Dr. GVR Rao]

In the absence of functioning ambulances based at facilities,[6, 22, 26], arranging an ambulance for IFT may be difficult and take time. The '108' ambulances are stationed close to CHCs and should be readily available, but if the ambulance is on route for pick-up or drop-off of another client then the IFT client with complications will have to wait or arrange for another means of transportation.[21] The '108' service may consider prioritising inter-facility transfers by having dedicated fleet of ambulances for IFTs (increase in efficiency and cost-effectiveness) over transporting all non-facility transfers by increasing the fleet of ambulances stationed closer to beneficiaries (increase in cost per beneficiary).[26] In some States, the under-utilised ambulances at the health facilities are being used through the '102' call centre along with '108', however the distribution of these into IFT and non-IFT use has not been well laid out.[21] In other States, '102' has new ambulances that focus on IFT or transfers back to home for pregnant women.[27] Since 2013, State of Assam has a dedicated fleet of 450 ambulances for IFTs operating through the '102' call system. Of these, 360 ambulances are managed by GVK

For peer review only - http://bmjopen.bmj.com/site/about/guidelines.xhtml

EMRI and just over half of all IFTs by these were for pregnant women in 2015-16.[Source: GVK EMRI annual statistics] This stresses the need for dedicated ambulances to deal with emergency IFTs, equipped with advanced life and obstetric support facilities.

The role of the '108' service in improving care at the referring and referral facility, and its overall impact on maternal morbidity and mortality reduction, could not be estimated from our analysis nor has been reported in other studies. Nonetheless, the '108' service is accepted as an important and successful intervention to improve patient transportation for obstetric, medical and other emergencies.[21, 24, 26, 35, 36] A study from five States, showed that '108' ambulances provide pre-hospital stabilising care to all pregnant women, and delivery and post-delivery care to those delivered at home or on the ambulance.[38] However, a study from Punjab found that about half of '108' ambulances did not meet required standards for basic life support.[36] Monitoring of the quality of en-route care and referral systems is integral for better outcomes and monitoring of any transport intervention.[16, 21, 39]

There are a few limitations to our analysis. Details of the type of emergency in the '108' database was based on the doctor's report or the diagnoses by the EMTs for IFTs, and only the EMTs for non-IFTs. This information is thus subject to inter-observer variability and differential reporting. However, we cannot estimate if this would have led to over-estimation or under-estimation of the effect estimate. A very few women who used '108' for antenatal care may have used it again for delivery care, and would have been counted twice. Data for treatment given en-route, and doctors' notes on inter-facility transfers, were mostly not recorded in the

For peer review only - http://bmjopen.bmj.com/site/about/guidelines.xhtml

'108' database and thus could not be studied. Details of the source hospital for IFTs were not available and thus details of transfers between type of facilities could not be assessed. There is a possibility that few calls for IFT for the women residing in rural areas were wrongly recorded, as they called from the health facilities in urban areas. This would have contributed to higher proportion of urban women among IFTs. The '108' service from Chhattisgarh was taken over by the '102' service since October 2013 thus use of '108' may be under-estimated for this State. We had large proportions of missing information on social and economic status from two States. We considered that the missingness was not at random and was not associated with outcome. There is evidence that in such situations a complete case analysis, as we reported, is associated with negligible bias compared to a multiple imputation approach.[40]

CONCLUSION

Of all the estimated institutional deliveries in India, only a very small proportion made use of the '108' ambulance for transfer between facilities. Among '108' users for IFTs around 92% did not have any complication or emergency. After adjusting for confounding factors, IFTs were more likely for women with obstetric emergencies, more than 30 years of age and from urban areas. Pregnant women from socially disadvantaged castes, below poverty line and from high priority districts were less likely to have IFTs. Utilisation of the '108' service and its determinants varied across States. Primary research is required to understand variation in utilisation and to explore the potential of the '108' ambulance service to serve a higher proportion of women requiring IFTs, in particular those having obstetric emergencies. The '108'

For peer review only

For peer review only - http://bmjopen.bmj.com/site/about/guidelines.xhtml

service would benefit by having a triage system to ensure that women with an obstetric emergency requiring an IFT are prioritised.

ACKNOWLEDGEMENTS

We acknowledge GVK-EMRI for sharing the data and allowing scientific study without any interference.

FUNDING

This work was supported by a Wellcome Trust Capacity Strengthening Strategic Award to the Public Health Foundation of India and a consortium of UK universities.

COMPETING INTERESTS

The authors have declared that no competing interests exist.

AUTHOR CONTRIBUTIONS

Conceived and designed the protocol: SS, PD, OC, GVR, GVS. Contributed in analysis plan: SS, PD, OC, LO, GVS. Analyzed and interpreted the data: SS. Contributed in preparation of manuscript: SS, PD, OC, LO, GVR, GVS.

ETHICS APPROVAL

The research obtained ethics approval from ethics committees of both LSHTM and IIPH-Hyderabad. (LSHTM Ethics Ref: 9613; IIPHH Ethics Ref: IIPHH/TRC/IEC/009/2014)

For peer review only - http://bmjopen.bmj.com/site/about/guidelines.xhtml

For peer review only

DATA SHARING

The data were obtained and analysed within the premises of GVK-EMRI office under license for the current study. The data are not publically available however can be available upon reasonable request directly to GVK-EMRI.

For peer review only

REFERENCES

1. National Rural Health Mission (NRHM). All India executive summary: status as on 31st March 2013. New Delhi: NRHM, Government of India 2013.

2. Registrar General of India. SRS: Maternal Mortality Ratio Bulletin 2011-2013. New Delhi: Government of India 2014. http://www.censusindia.gov.in/vital_statistics/mmr_bulletin_2011-13.pdf (accessed 30 September 2016).

3. WHO. Trends in maternal mortality: 1990 to 2013. Estimates by WHO, UNICEF, UNFPA, The World Bank and the United Nations Population Division. Geneva: WHO 2014. http://apps.who.int/iris/bitstream/10665/112682/2/9789241507226_eng.pdf (accessed 30 September 2016).

4. UNICEF. Levels & Trends in Child Mortality: Report 2015. Estimates Developed by the UN Inter-agency Group for Child Mortality Estimation United Nations Levels. New York: UNICEF 2015. http://www.unicef.org/publications/files/Child_Mortality_Report_2015_Web_9_Sept_15.pdf (accessed 30 September 2016).

For peer review only - http://bmjopen.bmj.com/site/about/guidelines.xhtml

5. WHO. The world health report 2005: Make every mother and child count. Geneva: WHO 2005. http://www.who.int/whr/2005/whr2005_en.pdf?ua=1 (accessed 30 September 2016).

6. Raj SS, Manthri S, Sahoo PK. Emergency referral transport for maternal complication: lessons from the community based maternal death audits in Unnao district, Uttar Pradesh, India. *International Journal of Health Policy and Management* 2015;4:99-106.

7. Singh S, Murthy GVS, Thippaiah A, Upadhyaya S, Krishna M, Shukla R, et al. Community based Maternal Death Review: Lessons learned from ten districts in Andhra Pradesh, India. *Maternal and Child Health Journal* 2015;19(7):1447-54.

8. WHO, UNFPA, UNICEF, AMDD. Monitoring emergency obstetric care: a handbook. Geneva: WHO 2009. http://apps.who.int/iris/bitstream/10665/44121/1/9789241547734_eng.pdf (accessed 30 September 2016).

9. Murraya SF, Pearson SC. Maternity referral systems in developing countries: Current knowledge and future research needs. *Social Science & Medicine* 2006;62:2205-15.

10. Chaturvedi S, Randive B, Diwan V, De Costa A. Quality of Obstetric Referral Services in India's JSY Cash Transfer Programme for Institutional Births: A Study from Madhya Pradesh Province. *PLoS ONE* 2014;9(5):e96773. doi:10.1371/journal.pone.0096773.

11. David KV, Pricilla RA, Venkatesan S, Rahman SP, G S YK, Vijayaselvi R. Outcomes of deliveries in a midwife-run labour room located at an urban health centre: results of a 5-year retrospective study. *National Medical Journal of India* 2012;25(6):323-6.

For peer review only - http://bmjopen.bmj.com/site/about/guidelines.xhtml

12. Iyengar K, Iyengar SD. Emergency obstetric care and referral: experience of two midwife-led health centres in rural Rajasthan, India. *Reproductive Health Matters* 2009;17(33):9-20.

13. Singh S, Doyle P, Campbell OM, Mathew M, Murthy GVS. Referrals between public sector health institutions for women with obstetric high-risk, complications, or emergencies in India –A systematic review. *PLoS ONE* 2016;11(8):e0159793. doi:10.1371/journal.pone.0159793

14. Raj SS, Manthri S, Sahoo PK. Emergency referral transport for maternal complication: lessons from the community based maternal death audits in Unnao district, Uttar Pradesh, India. *International Journal of Health Policy and Management* 2015;4:99-106.

15. Giovine A, Ostrowski C. Technical report on Improving Transportation and Referral for Maternal Health: Knowledge Gaps and Recommendations. Washington DC: 2010. https://www.wilsoncenter.org/sites/default/files/Transport%20Referral%20Meeting%20Technical%20Report.pdf (accessed 30 September 2016).

16. UNICEF. Operating Perinatal Referral Transport Services in Rural India: A toolkit. New Delhi: UNICEF 2010. https://www.mailman.columbia.edu/sites/default/files/pdf/unicef_2010_operating_perinatal_transport_services_in_rural_india_toolkit.pdf (accessed 30 September 2016).

17. Thaddeus S, Maine D. Too far to walk: maternal mortality in context. *Social science & medicine* 1994;38(8):1091-110.

18. Hussein J, Kanguru L, Astin M, Munjanja S. The effectiveness of emergency obstetric referral interventions in developing country settings: a systematic review. *Plos medicine* 2012;9(7):e1001264. doi:10.1371/journal.pmed.1001264.

For peer review only - http://bmjopen.bmj.com/site/about/guidelines.xhtml

19. Ministry of Health and Family Welfare. Directory of Innovations Implemented in the health Sector. New Delhi: MOHFW, Government of India 2008. http://planningcommission.nic.in/reports/genrep/health/Directory_of_innovations_march.pdf (accessed 30 September 2016).

20. Ministry of Health and Family Welfare. Operational Guidelines on Maternal and Newborn Health. New Delhi: MOHFW, Government of India 2010.

21. Sundararaman T, Chakraborty G, Nair A, Mokashi T, Ved R. Publicly Financed Emergency Response and Patient Transport Systems Under NRHM. New Delhi: Government of India 2012.

22. Ministry of Health and Family Welfare. Rural Health Statistics: 2014-15. New Delhi: Statistics Division, MOHFW, Government of India 2016. http://wcd.nic.in/sites/default/files/RHS_1.pdf (accessed 30 September 2016).

23. Kumutha J, Rao GVR, Sridhar BN, Vidyasagar D. The GVK EMRI maternal and neonatal transport system in India: A mega plan for a mammoth problem. *Seminars in Fetal & Neonatal Medicine* 2015;20(5).

24. Raj AX. Saving lives through rural ambulance services: Experiences from Karnataka and Tamil Nadu states, India. *Transport and Communications Bulletin for Asia and the Pacific*. 2014;84:52-66.

25. National Health Mission. Janani Shishu Suraksha Karyakaram (JSSK) 2012. http://www.nhp.gov.in/health-programmes/national-health-programmes/janani-shishu-suraksha-karyakaram-jssk (accessed 30 September 2016).

26. NHSRC. Emergency Medical servcie (EMS) in India: A concept paper. New Delhi: NRHM, MOHFW, Government of India 2012.

For peer review only - http://bmjopen.bmj.com/site/about/guidelines.xhtml

27. National Health Mission. ERS/Patient Transport Service: National Ambulance Service (NAS), MOHFW, Government of India 2015. http://nrhm.gov.in/nrhm-components/health-systems-strengthening/emri-patient-transport-service.html (accessed 30 September 2016).

28. GVK-EMRI. National footprints- Reaching unreachable Hyderabad, AP: GVK-EMRI 2015. http://www.emri.in/our-presence (accessed 30 September 2016).

29. Ministry of Health and Family Welfare. Guidance note for implementation of RMNCH+A interventions in High Priority Districts. New Delhi: MOHFW, Government of India 2013.

30. IIPS. Himachal Pradesh: District level household and facility survey (2012-13) Mumbai: MOHFW, Government of India 2014. https://nrhm-mis.nic.in/DLHS4/Reports/Himachal%20Pradesh.pdf (accessed 30 September 2016).

31. IIPS. Telangana: District level household and facility survey (2012-13) Mumbai: MOHFW, Government of India 2014. https://nrhm-mis.nic.in/DLHS4/Reports/Telangana.pdf (accessed 30 September 2016).

32. IIPS. Andhra Pradesh: District level household and facility survey (2012-13) Mumbai: MOHFW, Government of India 2014. https://nrhm-mis.nic.in/DLHS4/Reports/Andhra%20Pradesh.pdf (accessed 30 September 2016).

33. Registrar General of India. Annual Health Survey 2012-13 Factsheet: Chhattisgarh. New Delhi: Ministry of Home affairs, Government of India 2014 http://www.censusindia.gov.in/vital_statistics/AHSBulletins/AHS_Factsheets_2012-13/FACTSHEET-Chhattisgarh.pdf (accessed 30 September 2016).

34. The Institute of Health Systems. A study of Emergency Response Services in Three Districts of Andhra Pradesh. New Delhi: NHSRC, Government of India 2011.

For peer review only - http://bmjopen.bmj.com/site/about/guidelines.xhtml

35. Prinja S, Aggarwal AK, Kaur M. Evaluation of Haryana Emergency Ambulance Services. New Delhi: PGIMER, Chandigarh & NHSRC, 2011.

36. Prinja S, Bahuguna P, Lakshmi PVM, Mokashi T, Aggarwal AK, Kaur M, et al. Evaluation of publicly financed and privately delivered model of emergency referral services for maternal and child health care in India. *Plos ONE*. 2014;9(10): e109911. doi: 10.1371/journal.pone.0109911.

37. Prinja S, Jeet G, Kaur M, Aggarwal AK, Manchanda N, Kumar R. Impact of referral transport system on institutional deliveries in Haryana, India. *Indian Journal of Medical Research* 2014;139:883-91.

38. Strehlow MC, Newberry JA, Bills CB, Min HE, Evensen AE, Leeman L, et al. Characteristics and outcomes of women using emergency medical services for thirdtrimester pregnancy-related problems in India: a prospective observational study. *BMJ Open* 2016;6:e011459. doi:10.1136/bmjopen-2016-011459.

39. Murray SF, Davis S, Phiri KR, Ahmed Y. Tools for monitoring the effectiveness of district maternity referral systems. *Health Policy and Planning*. 2001;16:353-61.

40. White IR, John B. Carlin. Bias and efficiency of multiple imputation compared with complete-case analysis for missing covariate values. *Statistics in medicine* 2010;29(28):2920-31.

For peer review only

For peer review only - http://bmjopen.bmj.com/site/about/guidelines.xhtml

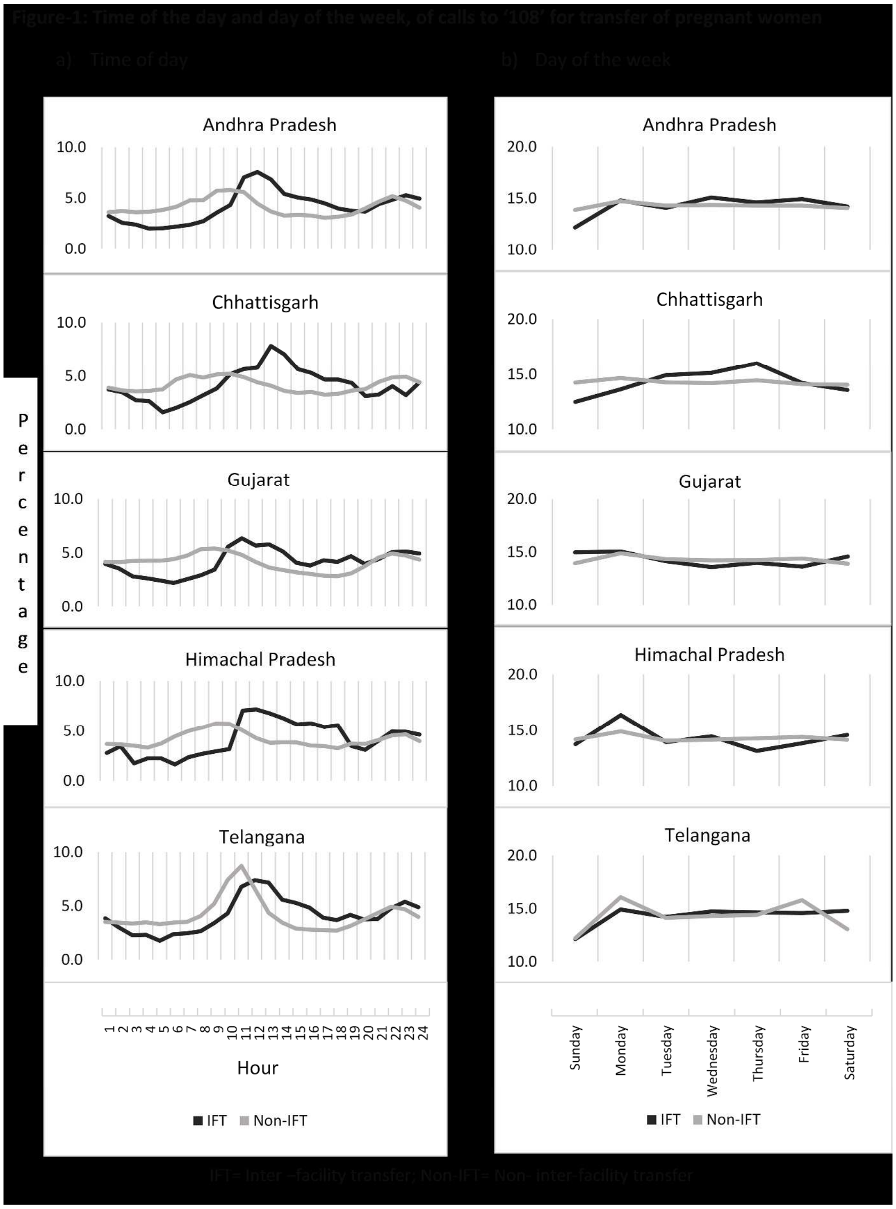

1042x1408mm (96 x 96 DPI)

STROBE 2007 (v4) Statement—Checklist of items that should be included in reports of *cross-sectional studies*

Section/Topic	Item #	Recommendation	Reported on page #
**Title and abstract**	1	(*a*) Indicate the study's design with a commonly used term in the title or the abstract	3
		(*b*) Provide in the abstract an informative and balanced summary of what was done and what was found	3,4
**Introduction**			
Background/rationale	2	Explain the scientific background and rationale for the investigation being reported	6-8
Objectives	3	State specific objectives, including any prespecified hypotheses	8
**Methods**			
Study design	4	Present key elements of study design early in the paper	9
Setting	5	Describe the setting, locations, and relevant dates, including periods of recruitment, exposure, follow-up, and data collection	9-10
Participants	6	(*a*) Give the eligibility criteria, and the sources and methods of selection of participants	10
Variables	7	Clearly define all outcomes, exposures, predictors, potential confounders, and effect modifiers. Give diagnostic criteria, if applicable	10-12
Data sources/ measurement	8*	For each variable of interest, give sources of data and details of methods of assessment (measurement). Describe comparability of assessment methods if there is more than one group	10
Bias	9	Describe any efforts to address potential sources of bias	12
Study size	10	Explain how the study size was arrived at	Universal sample
Quantitative variables	11	Explain how quantitative variables were handled in the analyses. If applicable, describe which groupings were chosen and why	11,12
Statistical methods	12	(*a*) Describe all statistical methods, including those used to control for confounding	11,12
		(*b*) Describe any methods used to examine subgroups and interactions	12
		(*c*) Explain how missing data were addressed	12
		(*d*) If applicable, describe analytical methods taking account of sampling strategy	NA
		(*e*) Describe any sensitivity analyses	12
**Results**			

For peer review only

Participants	13*	(a) Report numbers of individuals at each stage of study—eg numbers potentially eligible, examined for eligibility, confirmed eligible, included in the study, completing follow-up, and analysed	12,13
		(b) Give reasons for non-participation at each stage	13
		(c) Consider use of a flow diagram	NA
Descriptive data	14*	(a) Give characteristics of study participants (eg demographic, clinical, social) and information on exposures and potential confounders	15
		(b) Indicate number of participants with missing data for each variable of interest	15, Table-3
Outcome data	15*	Report numbers of outcome events or summary measures	14
Main results	16	(*a*) Give unadjusted estimates and, if applicable, confounder-adjusted estimates and their precision (eg, 95% confidence interval). Make clear which confounders were adjusted for and why they were included	17-20
		(*b*) Report category boundaries when continuous variables were categorized	17-20
		(*c*) If relevant, consider translating estimates of relative risk into absolute risk for a meaningful time period	17-20
Other analyses	17	Report other analyses done—eg analyses of subgroups and interactions, and sensitivity analyses	20-22
**Discussion**			
Key results	18	Summarise key results with reference to study objectives	23-26
Limitations	19	Discuss limitations of the study, taking into account sources of potential bias or imprecision. Discuss both direction and magnitude of any potential bias	27
Interpretation	20	Give a cautious overall interpretation of results considering objectives, limitations, multiplicity of analyses, results from similar studies, and other relevant evidence	27
Generalisability	21	Discuss the generalisability (external validity) of the study results	28
**Other information**			
Funding	22	Give the source of funding and the role of the funders for the present study and, if applicable, for the original study on which the present article is based	29

*Give information separately for cases and controls in case-control studies and, if applicable, for exposed and unexposed groups in cohort and cross-sectional studies.

Note: An Explanation and Elaboration article discusses each checklist item and gives methodological background and published examples of transparent reporting. The STROBE checklist is best used in conjunction with this article (freely available on the Web sites of PLoS Medicine at http://www.plosmedicine.org/, Annals of Internal Medicine at http://www.annals.org/, and Epidemiology at http://www.epidem.com/). Information on the STROBE Initiative is available at www.strobe-statement.org.

For peer review only

BMJ Open

Inter-facility transfer of pregnant women using publicly-funded emergency call-centre based ambulance services-Cross-sectional analysis of service logs from five states in India

Journal:	*BMJ Open*
Manuscript ID	bmjopen-2016-015077.R1
Article Type:	Research
Date Submitted by the Author:	23-Feb-2017
Complete List of Authors:	Singh, Samiksha; Indian Institute of Public Health-Hyderabad, PHFI<br>Doyle, Pat ; London School of Hygiene and Tropical Medicine, Faculty of Epidemiology and Population Health, Department of Non-communicable Disease Epidemiology<br>Campbell, Oona; London School of Hygiene and Tropical Medicine, Faculty of Epidemiology and Population Health, Department of Infectious Disease Epidemiology<br>Oakley, Laura; London School of Hygiene and Tropical Medicine, Faculty of Epidemiology and Population Health, Department of Non-communicable Disease Epidemiology<br>Rao, G.V. Ramana; GVK Emergency Management and Research Institute, Emergency Medicine Learning Center and Research<br>Murthy, GVS; Indian Institute of Public Health, Hyderabad, PHFI, Director; London School of Hygiene and Tropical Medicine, Faculty of Infectious and Tropical Diseases, Department of clinical research
**Primary Subject Heading**:	Public health
Secondary Subject Heading:	Health services research, Public health, Epidemiology, Obstetrics and gynaecology
Keywords:	Maternal medicine < OBSTETRICS, Ambulance, Inter-facility transfer, Obstetric emergency, Obstetric complication, Patient transport

SCHOLARONE™ Manuscripts

Title: Inter-facility transfer of pregnant women using publicly-funded emergency call-centre based ambulance services- Cross-sectional analysis of service logs from five states in India

Short title: Inter-facility transfer of pregnant women in India using 108 ambulances

Samiksha Singh, Pat Doyle, Oona M R Campbell, Laura Oakley, GVR Rao, GVS Murthy

Samiksha Singh, (Corresponding author)

Assistant Professor,

Indian Institute of Public Health-Hyderabad, Public Health Foundation of India

Address- Plot#1 AVN Arcade, Amar Co-operative society, Kavuri Hills, Madhapur, Hyderabad, Telanagana, India - 201010; Email- samiksha.singh@iiphh.org; Phone- +91 8886 111 290

Pat Doyle

Professor,

Department of Non-communicable disease epidemiology, Faculty of Epidemiology and Population Health, London School of Hygiene and Tropical Medicine, London, UK

Oona M Campbell

Professor,

Department of Infectious Disease Epidemiology, Faculty of Epidemiology and Population Health, London School of Hygiene and Tropical Medicine, London, UK

For peer review only

Laura Oakley

Assistant Professor,

Department of Non-communicable disease epidemiology, Faculty of Epidemiology and Population Health, London School of Hygiene and Tropical Medicine, London, UK

GV Ramana Rao

Director,

Emergency Medicine Learning Center and Research, GVK-Emergency Management and Research Institute, Hyderabad, India

GVS Murthy

Professor,

Indian Institute of Public Health-Hyderabad, Public Health Foundation of India, Hyderabad, India and Department of clinical research, Faculty of Infectious and Tropical Diseases, London School of Hygiene and Tropical Medicine, London, UK

Word count: 4630

Key words: Ambulance, Inter-facility transfer, Obstetric emergency, Obstetric complication, Pregnant women, Patient transport, Access, Maternity Services, Travel time

For peer review only

For peer review only - http://bmjopen.bmj.com/site/about/guidelines.xhtml

ABSTRACT

Objective: To estimate the proportion of Inter-facility transfers (IFTs) transported by '108' ambulances and to compare the characteristics of the IFTs and non-IFTs to understand the pattern of use of '108' services for pregnant women in India.

Design: A cross-sectional analysis of '108' ambulance records from five states for the period April 2013 to March 2014. Data were obtained from the call-centre database for the pregnant women, who called '108'.

Main outcomes: Proportion of all pregnancies and institutional deliveries in the population who were transported by '108', both overall and for IFT. Characteristics of the women transported; obstetric emergencies, the distances travelled, and the time taken for both IFT and non-IFT.

Results: The '108' ambulances transported 608,559 pregnant women, of whom 34,993 were IFTs (5.8%) in the five states. We estimated that '108' transferred 16.5% of all pregnancies and 20.8% of institutional deliveries. Only 1.2% of all institutional deliveries in the population were transported by '108' for IFTs—lowest 0.6% in Gujarat and highest 3.0% in Himachal Pradesh. Of all '108' IFTs, only 8.4% had any pregnancy complication. For all states combined, on adjusted analysis, IFTs were more likely than non-IFTs to be for older and younger women or from urban areas, and less likely to be for women from high-priority districts, from backward or scheduled castes, or women below-the-poverty-line. Obstetric emergencies were more than twice as likely to be IFTs as pregnant women without obstetric emergencies (OR=2.18, 95% CI, 2.09-2.27). There was considerable variation across states.

For peer review only - http://bmjopen.bmj.com/site/about/guidelines.xhtml

For peer review only

Conclusion: Only 6% institutional deliveries made use of the '108' ambulance for IFTs in India. The vast majority did not have any complication or emergency. The '108' service may need to consider strategies to prioritise the transfer of women with obstetric emergency and those requiring IFT, over uncomplicated non-IFT.

For peer review only

For peer review only - http://bmjopen.bmj.com/site/about/guidelines.xhtml

STRENGTHS & LIMITATIONS

- This study is the first to assess the role of the '108' ambulance service - the largest provider of the free emergency medical services in India – for IFTs of pregnant women.
- We assessed the characteristics of pregnant women who were transported by '108' as IFTs and compared them with those who were transported by '108' as non-IFTs, for five state.
- We did extensive cleaning and management of data to drive appropriate information.
- '108' service did not record information on post-partum obstetric emergency separately thus these could not be estimated.
- Diagnosis of obstetric emergency may be inaccurate and subject to inter-observer bias.
- The '108' database mostly did not record data for treatment given en-route, and doctors' notes on IFTs, thus we could not study these data.
- Some population estimates are based on assumptions and may not be accurate.
- We had large proportions of missing information on social and economic status from two states. We did a complete analysis and sensitivity analysis to deal with missingness.

For peer review only

For peer review only - http://bmjopen.bmj.com/site/about/guidelines.xhtml

BACKGROUND

India had an estimated 83% women delivering in health facilities in 2013.[1] Despite this high proportion of institutional births, India had an estimated maternal mortality ratio (MMR) of 167/ 100,000 live births and an early neo-natal mortality rate of 28/1000 live births in 2013.[2] The country accounts for 17% (50,000) of global maternal deaths per year[3] and 26% (696,000) of global neonatal deaths.[4] Many maternal deaths occur during transit to health facilities.[5-7]

Inter-facility transfers (IFTs) for pregnant women are crucial, especially in resource poor countries where most peripheral health facilities provide only uncomplicated birthing or basic emergency obstetric care.[8,9] About 14% to 36% of women delivering in facilities are referred from lower to higher-level facilities.[10-13] As IFT is more likely to be due to referral for high risk pregnancies or complications during pregnancy, childbirth or post-partum, it can play a pivotal role in reduction of maternal morbidity and mortality.[14, 15]

Ensuring an uneventful IFT is part of good health care provision at the referring facility, and will reduce delays in access to appropriate health care (delay type-2) and delays in getting appropriate care after reaching a health facility (delay type-3).[16, 17] A good process will include prompt arrangement of transport, en-route stabilisation, communication with the referral facility to prepare them for the patient, and appropriate hand-over on arrival. IFT is thus a complex coordinated effort made by the referring health care provider, the en-route attendant, the receiver at the referral health facility, and the referral transport system.[16] Several successful interventions have been reported in resource-poor countries to improve

For peer review only

referral transportation, but only a few mention IFT.[18, 19] Referral transport for IFT may be a) an ambulance based at the referring facility, b) an ambulance called from a referral facility, c) an independent ambulance service, d) other subsidised public or commercial transport, or e) personal or commercial (non-subsidised) transport.[16, 20]

India, currently, does not have any structured inter-facility referral and transportation protocols for pregnant women (desk review and personal communication with Maternal health specialist, Government of India). In India, most peripheral health facilities do not have functional ambulances of their own and if they do, they are not available round the clock.[14, 21, 22] Thus IFT for pregnant women depends on other referral transport services, the majority using the free, public funded '108'/'102' call centre based ambulance services[19, 23, 24] and paid subsidised, commercial, or personal transport. There are other services across states that, unlike '108', are basic patient transportation services with no en-route stabilisation care. These may also provide transport between facilities.[21, 25, 26] For example, Janani Express yojana (public private non-ambulance transport service), operating through call-centres within districts, transfers exclusively pregnant women and newborns in Madhya Pradesh and Odisha.[21, 25] In larger cities, some other ambulance services (free or paid) are also available.

There have been very few research studies on transportation for IFTs of pregnant or postpartum women in India, despite the large investments made in public referral transport. This study was conducted to investigate IFTs of pregnant women in India using '108' ambulance services. The objectives of the research were to i) estimate proportion of women transferred by

For peer review only - http://bmjopen.bmj.com/site/about/guidelines.xhtml

'108' among all institutional deliveries in the general population, ii) estimate the proportion of IFTs transferred by '108' among all institutional deliveries in the population, iii) estimate the proportion of IFTs among all transfers of pregnant women by '108', and iii) compare the characteristics of the IFTs and non-IFTs.

METHODS

Context

The '108' ambulance service operates under a public private partnership. It operates 7361 ambulances, and transfers any medical emergency across 21 states and union territories (smaller less populated administrative units in India).[26] GVK-EMRI is the largest service provider for '108' and operates in 15 states and 2 union territories. In 2014-15, GVK-EMRI '108' ambulances transferred about 3.6 million pregnant women to health facilities, which were about two-fifths of all the transfers by GVK-EMRI '108'.[23] There is approximately one ambulance for every 100,000 population, and the ambulance should be well equipped and accompanied by an Emergency Medicine Technician (EMT). The EMT is trained to provide emergency care and basic life support in obstetrics. In cases of imminent childbirth, the EMT is expected to assist the delivery en-route and transfer the mother and child to the nearest health facility.[27] For IFTs, the EMT consults the '108' call-centre based medical officer and the referring health care provider to discuss indication of referral, stability of case to withstand travel, stabilising care required and place of referral [Source: Expert from GVK-EMRI). '108' service preferably transfers mother to public facilities, however in absence of appropriate public facility nearby, they transfer the mother to empaneled private facility.[27]

For peer review only - http://bmjopen.bmj.com/site/about/guidelines.xhtml

Working definitions

Inter-facility transfer (IFT), for this study, was defined as any transfer of a pregnant woman from one health facility to another health facility on the advice of a health care provider, using a '108' ambulance. All other transfers of pregnant women to health facilities using '108' ambulances were defined as 'non-IFT'. These mostly included transfers from home to facility.

An obstetric emergency was defined as any life threatening medical complication in women in pregnancy, labour or childbirth, or after (within 42 days of termination of pregnancy). For IFT women diagnosis of obstetric emergency was made by the referring doctor and noted by EMT after discussion with call-centre based doctor. For non-IFT, diagnosis was made by EMT. We observed that even high-risk such as previous caesarean section and precious pregnancy were considered obstetric emergency by '108' services thus we included them.[28]

Study Design

This cross-sectional study analysed '108' ambulance records from five states. Ethical approval for the study was obtained from Indian Institute of Public Health-Hyderabad and London School of Hygiene and Tropical Medicine.

Study population

Pregnant women who called '108' or for whom a relative or friend or health care provider called on their behalf, between 1st April 2013 and 31st March 2014 in the five states in India where GVK-EMRI operated '108' service had been fully functional for more than 3 years were

For peer review only - http://bmjopen.bmj.com/site/about/guidelines.xhtml

included in this analysis (Andhra Pradesh, Chhattisgarh, Gujarat, Himachal Pradesh, and Telangana). The period 1st April to 31st March was aligned with reporting period of state governments. States were chosen in a manner that they had representation of North, South, East and West of India.

Obtaining data and data management

Formal permission to use the data was obtained from GVK-EMRI. Anonymised information on '108' calls from 1st April 2013 to 31st March 2014 was obtained from the GVK-EMRI emergency response centre database. Details on data management are described in another paper form the same dataset.[28] Data were extracted onto Excel sheets, and converted to STATA 10.0 files. Data were inspected to assess consistency, range, and missing data. Any gross issue relating to the quality of records was noted, and records with improbable entries were excluded from analysis. Variables were recoded wherever needed. Variables of interest were: IFT; age of pregnant woman; social caste (general, other backward, schedule tribe and scheduled caste); economic class (below or above poverty line); area (rural or urban); type of emergency; time of call; day of call; time taken by ambulance to reach the client; time taken to reach the health facility; and distance travelled. Castes are classified based on historically backward and deprived social castes. The information was provided by the caller and EMT, and recorded in the call-centre database. Districts within states were stratified into high priority districts and non-high priority districts. High priority districts are those identified by the Government of India as being in the lowest quartile of districts (or tribal districts) in each state

For peer review only - http://bmjopen.bmj.com/site/about/guidelines.xhtml

with respect to maternal and child health indicators (including institutional delivery rate, maternal mortality and neonatal mortality rates among a total of 16 indicators).[29]

Analysis

We used information from the Census 2011 , District level household survey (DLHS) 2012-13,[30-32] and Annual health survey (AHS) 2012-13,[33] to estimate the number of pregnancies and institutional deliveries in the population for the study states, as mentioned below. The numbers of pregnant women transported by '108' as recorded in the call-centre database were compared with these population estimates for each state.

For each state, the number of pregnancies in the study period was estimated as sum of estimated pregnancies in rural and urban population [population (rural) X crude birth rate (rural) X 1.1 X 1000] + [population (urban) X crude birth rate (urban) X 1.1 X 1000]. The population data were obtained from the 2011 census and the crude birth rates from the Sample Registration System 2013. The multiplier 1.1 is used to account for an estimated 10% of the pregnancies which may have ended in abortions or intra-uterine deaths.[34]

The number of institutional deliveries in the study period was estimated as [Estimated number of pregnancies (rural) X institutional delivery rate (rural) X 100] + [Estimated number of pregnancies (urban) X institutional delivery rate (urban) X 100]; Institutional delivery rates include live births and still-births. The institutional delivery rate were obtained from DLHS-4 and AHS-2 surveys.

For peer review only - http://bmjopen.bmj.com/site/about/guidelines.xhtml

Information was analysed for all states combined and separately by State, comparing IFT and non-IFT. The characteristics of the women transported, distances travelled and the time taken by '108' ambulances, were described for both IFT and non-IFT journeys. The association between socio-demographic and clinical variables and the outcome (IFT vs no-IFT) was investigated using bivariate and multivariate logistic regression.

Social caste and economic class were missing in 55% and 95% of observations in the state of Chhattisgarh, and 14% and 22% in Himachal Pradesh. Given the nature of the variables we decided that it was likely that the data were missing not at random, and therefore multiple imputation was considered inappropriate. In order to investigate possible selection bias resulting from missing data, we performed a complete case analysis for each state and all states combined, supplemented by a series of sensitivity analyses. Our first sensitivity analysis involved running a model with and without states of Chhattisgarh and Himachal Pradesh. Presence or absence of these states did not substantially change the pattern of results. The second sensitivity analysis was performed by running a model with and without social caste and economic class for all States combined (total) and for individual states. The presence or absence of these variables did not substantially change the magnitude of the pattern of results (Odds ratios and Rsq for each model) for the total and for states (except Chhattisgarh). For our main analysis (all States combined) we thus included social caste and economic class variables and excluded any data from Chhattisgarh. Models for individual states (except Chhattisgarh) also include social caste and economic class.

For peer review only - http://bmjopen.bmj.com/site/about/guidelines.xhtml

For peer review only

RESULTS

Study Populations

The study states had population sizes ranging from 6 million to 60 million and had different social compositions (Table-1). The percentage of rural population ranged from 61% in Telangana to 90% in Himachal Pradesh while scheduled castes and tribes, together, ranged from 22% in Andhra Pradesh and Gujarat to 43% in Chhattisgarh. The crude birth rate was lowest in Himachal Pradesh (1.6%) and highest in Chhattisgarh (2.5%). The states also varied in the institutional delivery rates from 39.5% in Chhattisgarh to 94.1% in Telangana, and in their MMRs from 244 in Chhattisgarh to 92 per 100,000 live births in Telangana and Andhra Pradesh. On an average '108' ambulance is sanctioned for approximately 100,000 population in all states except Himachal Pradesh where one ambulance caters to about 40,000 population. Himachal Pradesh is mostly hilly and has sparse population distribution and '108' ambulances are stationed based on geographic regions.

Table 1: Demographic characteristics of the study states

	Andhra Pradesh	Chhattisgarh	Gujarat	Himachal Pradesh	Telangana
Total Population*	49,386,799	25,540,196	60,383,628	6,856,509	35,193,978
Rural/tribal	70.4%	76.8%	57.4%	90.0%	61.3%
Urban	29.6%	23.2%	42.6%	10.0%	38.7%
Scheduled caste*	17.1%	12.8%	6.8%	25.2%	15.4%
Scheduled tribe*	5.3%	30.6%	14.8%	5.7%	9.3%
Crude birth rate per 1000 population**	17.5	24.5	21.1	16.2	17.5
Institutional delivery rate per 100 childbirth***	88.5%	39.5%	78.1%	77.8%	94.1%
MMR per 100,000 livebirths (2011-13)**	92	244	112	Not available	92
NMR per 1,000 livebirths (2012-13)**	25	31	26	25	25

For peer review only - http://bmjopen.bmj.com/site/about/guidelines.xhtml

No. of Ambulances under '108'#	468	240	506	171	334
Geographic region/terrain	Tribal pockets	Large Tribal; hilly pockets	Tribal pockets	Majority hilly	Tribal pockets

Source- *Census 2011; ** Sample Registration System 2013- Separate data for Telangana and Andhra Pradesh not available; *** DLHS (2012-13) / AHS (2011-12); # GVK-EMRI annual report for period April 2013- March 2014. $In Chhattisgarh '102' ambulance service took over from October 2013 – March 2014.

Description of 108 calls relating to pregnancy

Across the five states, '108' call centres received 646,656 calls for pregnancy-related transfers, 6.2% of which were for IFT. Among IFT calls, an ambulance was not assigned to 1.2%, and an ambulance was assigned but not used by 6.0% of callers. Among non-IFT callers these proportions were 1.4% and 3.9% respectively. A total of 608,559 pregnant women were transported using '108', and of these journeys 5.8% were for IFT.

Estimated proportions of pregnancies, institutional deliveries and IFTs transported by '108'

Table-2 presents estimates of the number of pregnant women, obstetric emergencies, institutional deliveries and IFTs in the general populations, and the proportion of these transported using '108' ambulances in the five states, and overall. The 608,559 pregnant women transferred comprised 16.5% of all estimated pregnancies, and 20.8% of all estimated institutional deliveries for the study states combined.

Of the estimated institutional deliveries, '108' transported only 1.2% (34,993) women between facilities across States. This proportion was highest in Himachal Pradesh (3.0%) followed by

For peer review only

Andhra Pradesh (1.7%), Telangana (1.4%), Chhattisgarh (0.7%) and lowest in Gujarat (0.6%). Only 1.0% (28,448) of all institutional deliveries were transported by '108' for obstetric emergencies—highest 2.4% in Himachal Pradesh and lowest 0.6% in Andhra Pradesh and Telangana (Table-2).

Table 2: Estimated proportion of pregnant women transported to hospitals by '108' from April 2013 to March 2014

	Total	Andhra Pradesh	Chhattisgarh [$]	Gujarat	Himachal Pradesh	Telangana
Estimated number of pregnancies in the population [a]	3,811,920	950,696	678,217	1,387,021	121,638	673,508
Estimated number of institutional deliveries in the population [b]	2,921,674	842,109	267,896	1,083,264	94,634	633,771
Proportion of all pregnancies transported by '108' [c]	16.5%	15.5%	9.0%	19.5%	20.5%	15.7%
Proportion of all institutional deliveries transported by '108' [d]	20.8%	17.5%	22.7%	24.9%	26.3%	16.6%
Proportion of all institutional deliveries that were transported by '108' for inter-facility transfers [e]	1.2%	1.7%	0.7%	0.6%	3.0%	1.4%
Proportion of institutional deliveries that were transported by '108' for obstetric emergencies [f]	1.0%	0.6%	1.0%	1.4%	2.4%	0.6%

[a] [population (rural) X crude birth rate (rural) X 1.1 X 1000] + [population (urban) X crude birth rate (urban) X 1.1 X 1000]; Using Census populations and crude birth rates from Sample Registration Survey in respective states

[b] [Estimated number of pregnancies (rural) X institutional delivery rate (rural) X 100] + [Estimated number of pregnancies (urban) X institutional delivery rate (urban) X 100]; Institutional delivery rates include live births and still births. Using institutional delivery rates from District Level Household Survey / Annual Household Survey in respective states

[c] Number of pregnant women transported by '108'/Estimated number of pregnancies in the population([a])

[d] Number of pregnant women transported by '108'/Estimated number of institutional deliveries in the population([b])

[e] Number of pregnant women transported between facilities (IFTs) by '108'/ estimated number of institutional deliveries in the population([b])

[f] Number of pregnant women transported by '108' for obstetric emergency / estimated number of institutional deliveries in the population([b])

[$]In Chhattisgarh '102' ambulance service took over from '108' service from October 2013 to March 2014. Thus number of beneficiaries of '108' and proportions may be underestimation of utilization for annual estimates.

Characteristics of pregnant women transferred by '108' ambulances

For peer review only - http://bmjopen.bmj.com/site/about/guidelines.xhtml

The pregnant women who were transferred by '108' belonged mostly to lower social and economic sections- rural or tribal areas (84.6%) or scheduled castes or tribes (64.9%) and below-the-poverty-line status (76.6%). Two hundred and forty two women (40 per 100,000) died before the ambulance reached the pick-up site. The proportion of pregnant women who died before arrival of '108' ambulance was higher in Chhattisgarh (150 per 100,000) compared to other states.

Of the pregnant women transferred by '108' ambulances, 34,993 (5.8%) had an IFT (Table-3). The proportion of IFTs among women transported by '108' was highest in Himachal Pradesh (11.3%) followed by Andhra Pradesh (9.9%), Telangana (8.7%), Chhattisgarh (3.2%) and Gujarat (2.4%).

Overall, IFTs were made up of a higher proportion of younger women, women from backward caste, belonging to below-the-poverty-line and from urban areas compared to non-IFTs. A Similar pattern was observed across all the states except in Himachal Pradesh where IFTs had lower proportion of women from below-the-poverty-line strata compared to non-IFTs (Table-3). Delivery en-route or in the ambulance was less likely among IFTs compared to non-IFTs across all states.

For peer review only - http://bmjopen.bmj.com/site/about/guidelines.xhtml

Table 3: Demographic characteristics of users of '108' ambulance service for IFT and non-IFT in states (April 2013 to March 2014)

	Total		Andhra Pradesh		Chhattisgarh		Gujarat		Himachal Pradesh		Telangana	
	IFT (N=34,993) [5.8%]	Non-IFT (N=573,566) [94.2%]	IFT (N=14,574) [9.9%]	Non-IFT (N=132,800) [90.1%]	IFT (N=1,929) [3.2%]	Non-IFT (N=58,881) [96.8%]	IFT (N=6,562) [2.4%]	Non-IFT (N=263,509) [97.6%]	IFT (N=2,809) [11.3%]	Non-IFT (N=22,114) [88.7%]	IFT (N=9,119) [8.7%]	Non-IFT (N=96,262) [91.3%]
Age group (years)												
<19	4.1	3.4	5.0	4.8	7.1	5.8	2.3	2.0	4.4	5.7	3.5	3.6
20 – 24	58.7	56.5	64.9	65.7	54.9	56.4	52.0	51.9	48.3	51.6	58.6	58.9
25 – 30	28.1	30.6	24.0	24.9	25.0	27.9	30.1	33.2	34.5	32.0	31.4	32.2
30 – 34	6.5	7.2	4.2	3.3	8.9	7.2	11.3	9.9	9.4	8.0	4.8	4.1
>35	2.6	2.3	1.9	1.2	4.1	2.7	4.3	3.0	3.4	2.7	1.7	1.2
Total non-missing	(32,538) 100	(551,568) 100	(13,105) 100	(120,155) 100	(1,914) 100	(58,591) 100	(6,562) 100	(263,508) 100	(2,779) 100	(21,694) 100	(8,178) 100	(87,620) 100
missing	(2,455) 7.0	(21,988) 3.8	(1,469) 10.1	(12,645) 9.5	(15) 0.8	(290) 0.5	(0) 0	(1) 0	(30) 1.1	(420) 1.9	(941) 10.3	(8,642) 9.0
Social caste												
General caste	12.2	11.3	10.8	10.9	3.7	3.5	14.8	12.8	37.3	38.2	7.1	5.0
Other backward	40.0	38.5	41.7	44.1	48.5	43.8	38.3	35.0	17.4	11.5	43.4	43.5
Scheduled caste	29.1	22.1	34.7	34.1	14.3	14.7	11.2	11.1	37.2	42.8	32.2	34.2
Scheduled tribe	18.7	28.2	12.8	10.9	33.6	38.1	35.8	41.2	8.1	7.6	17.3	17.3
Total non-missing	(32,800) 100	(535,328) 100	(14,400) 100	(131,924) 100	(652) 100	(27,427) 100	(6,551) 100	(263,204) 100	(2,201) 100	(17,148) 100	(8,996) 100	(95,625) 100
DK/ missing	(2,193) 6.3	(38,238) 6.7	(174) 1.2	(876) 0.7	(1,277) 66.2	(31,454) 53.4	(11) 0.2	(305) 0.1	(608) 21.6	(4,966) 22.5	(123) 1.3	(637) 0.7
Economic class												
BPL	87.7	75.9	99.7	99.6	96.7	97.0	54.2	55.9	62.7	67.0	99.5	99.5
Others	12.3	24.1	0.3	0.4	3.3	3.0	45.7	44.1	37.3	33.0	0.5	0.5
Total non-missing	(32,296) 100	(511,447) 100	(14,357) 100	(131,391) 100	(61) 100	(2824) 100	(6,540) 100	(262,991) 100	(2,395) 100	(19,041) 100	(8,943) 100	(95,200) 100
DK/ missing	(2,697) 7.7	(62,119) 10.8	(217) 1.5	(1,409) 1.1	(1,868) 96.8	(56,057) 95.2	(22) 0.3	(518) 0.2	(414) 14.7	(3,073) 13.9	(176) 1.9	(1,062) 1.1
Area												
Rural/tribal	68.7	85.6	67.4	79.5	92.1	92.7	62.8	89.3	75.9	95.2	67.6	77.8
Urban	31.3	14.4	32.6	20.5	7.9	7.3	37.2	10.7	24.1	4.8	32.4	22.2
Total non-missing	(34,910) 100	(570,042) 100	(14,555) 100	(132,510) 100	(1,929) 100	(58,881) 100	(6,562) 100	(263,509) 100	(2,770) 100	(18,952) 100	(9,094) 100	(96,190) 100
Missing	(78) 0.2	(3,524) 0.6	(19) 0.1	(290) 0.2	(0) 0	(0) 0	(0) 0	(0) 0	(39) 1.4	(3,162) 14.3	(25) 0.3	(72) 0.1
Died before ambulance reached	0.07	0.04	0.04	0.02	0.36	0.14	0.02	0.03	0	0.01	0.02	0.02
Delivery en-route												
At pick up site	0.3	1.4	0.2	0.8	1.2	2.8	0.5	1.7	0.2	1.7	0.2	0.7
In ambulance	0.6	1.0	0.4	0.7	0.8	1.5	0.6	0.9	1.7	3.5	0.4	0.7

IFT= Inter-facility transfer ; non-IFT = non-inter-facility transfer

For peer review only - http://bmjopen.bmj.com/site/about/guidelines.xhtml

For peer review only

Obstetric emergencies among women transferred by '108' ambulance for IFT and non-IFT

The majority of transfers by '108' ambulances were for normal labour. By state, between 2.7% and 9.3% of transferred pregnant women had an obstetric emergency; the overall average was 4.8%. The IFTs (8.4%) had higher proportion of pregnant women who had an obstetric emergency compared to non-IFTs (4.4%) (Table-4). Himachal Pradesh had highest proportion of obstetric emergencies transferred for both IFT (21.0%) and non-IFT (7.8%). Andhra Pradesh was the lowest proportion of obstetric emergencies transferred for IFT (5.4%) and non-IFT (3.1%).

The most common obstetric emergencies were 'abnormal presentation of foetus' and 'bleeding in pregnancy' in Telangana, Andhra Pradesh and Gujarat; and 'bleeding in pregnancy' and 'medical conditions complicating pregnancy' in Himachal Pradesh and Chhattisgarh. IFTs had higher proportions of 'bleeding in pregnancy' cases across all the states and 'medical conditions complicating pregnancy' in Himachal Pradesh and Chhattisgarh (Table-4). In Telangana and Andhra Pradesh, some IFTs (0.5% and 0.2%) and non-IFTs (8.43% and 0.53%) were for antenatal women requiring check-ups at higher institutions (data not shown).

Destination facilities

About 86% of all transfers were to government or government supported hospitals. This figure was highest in Himachal Pradesh (97%) and lowest in Andhra Pradesh where around 82% of women were transferred to government hospitals (table-4). With type of government facility, across the States, half of the IFTs were to district level secondary or tertiary hospitals while

For peer review only - http://bmjopen.bmj.com/site/about/guidelines.xhtml

non-IFTs were to sub-district Area or Civil hospitals or CHCs. Gujarat reported highest percentage of transfers to middle level facilities. Himachal Pradesh had more transfers to Civil-hospitals or higher (Table-4).

Distance and time travelled by 108 ambulance for pregnant women

The median distances travelled and time taken by '108' ambulances to transfer pregnant women are shown in Table-4. Ambulances travelled less than 4 kilometres to reach half of the pregnant women for IFTs across four States but up to 10 kilometres in Andhra Pradesh. For non-IFTs, ambulances travelled farther (between 9 and 12 kilometres) to reach half of the pregnant women. Among IFTs median distances from pick-up site to destination facility were between 16 kilometres in Chhattisgarh to 32 kilometres in Himachal Pradesh, but nearer in non-IFTs (between 8 and 32).

The median time taken for the ambulance to travel to the pick-up site for IFTs was between 10 minutes and 15 minutes for four states but longer (24 minutes) in Andhra Pradesh. The median time taken for travel from the referring to the destination facility was less than 40 minutes in Andhra Pradesh, Chhattisgarh and Gujarat but longer for Telangana (48 minutes) and Himachal Pradesh (83 minutes). For non-IFT, these times were about 30 minutes each, except for Himachal Pradesh where travel times were longer (Table-4).

For peer review only - http://bmjopen.bmj.com/site/about/guidelines.xhtml

For peer review only

Table 4: Characteristics of transfers for IFT and non-IFT by state (April 2013 to March 2014)

	Total		Andhra Pradesh		Chhattisgarh		Gujarat		Himachal Pradesh		Telangana	
	IFT (N=34,993)	Non-IFT (N=573,566)	IFT (N=14,574)	Non-IFT (N=132,800)	IFT (N=1,929)	Non-IFT (N=58,881)	IFT (N=6,562)	Non-IFT (N=263,509)	IFT (N=2,809)	Non-IFT (N=22,114)	IFT (N=9,119)	Non-IFT (N=96,262)
Obstetric emergency, %	8.4	4.4	5.4	3.1	18.0	3.9	10.4	5.5	21.0	7.8	5.8	3.2
Abnormal presentation	1.5	2.0	1.0	1.0	1.3	1.2	3.2	3.1	0.7	0.4	1.3	1.1
Bleeding in pregnancy	3.5	0.9	2.0	0.6	1.6	0.4	4.5	1.0	14.1	5.6	2.1	0.6
Eclampsia/ convulsion	0.7	0.1	0.6	0.2	0.7	0.1	0.6	0.1	1.1	0.1	0.8	0.2
Foetal loss	0.2	0.1	0.1	0.1	0.6	0.4	0.1	0.02	0.4	0.1	0.1	0.1
Medical condition	1.8	0.7	0.9	0.6	13.6	1.7	1.0	0.4	3.8	1.1	0.8	0.8
Previous caesarian	0.4	0.4	0.5	0.3	0.3	0.2	0.5	0.5	0.3	0.3	0.4	0.3
Precious pregnancy	0.3	0.3	0.2	0.2	0.1	0.1	0.5	0.4	0.5	0.2	0.3	0.2
Other emergency, %	3.7	1.8	4.1	2.8	8.7	1.2	2.3	1.0	0	0	4.2	3.5
Destination facility, %												
Government	82.0	79.6	82.1	81.7	87.6	96.8	70.2	72.0	96.0	97.6	85.3	84.1
District Secondary or tertiary hospital	53.2	19.8	54.2	29.4	64.3	28.5	30.1	7.3	86.9	46.7	55.7	32.0
Area / Civil hospital	19.8	13.4	18.1	21.1	0.6	0.8	31.5	8.3	7.7	27.3	21.3	22.5
Community Health Centre	7.6	34.6	8.3	19.7	20.5	57.4	7.4	43.6	0.9	20.4	6.3	16.2
Primary Health Centre	1.1	9.0	1.2	11.3	1.9	9.3	0.2	7.0	0.3	3.2	1.9	13.1
Sub-Health Centre	0	0.1	0	0	0.3	0.9	0	0	0	0	0	0
Information Missing	0.4	2.8	0.3	0	0	0	0.9	5.6	0	0	0.2	0.2
Government supported	3.4	7.7	0	0	0	0	16.9	15.9	0	0	0	0
Private	14.5	12.8	17.9	18.3	12.4	3.2	12.9	12.1	4.0	2.4	14.7	15.9
Total non-missing	(31,911) 100	(531,392) 100	(12,693) 12.9	(116,028) 100	(1,784) 100	(54,093) 100	(6,442) 100	(256,898) 100	(2,777) 100	(21,449) 100	(8,215) 100	(82,924) 100
Missing	(3082) 8.8	(42,174) 7.4	(1,881) 12.9	(16,772) 12.6	(145) 7.5	(4,788) 8.1	(120) 1.8	(6,611) 2.5	(32) 1.1	(665) 3.0	(904) 9.9	(13,338) 13.9
To different district	32.1	28.5	29.8	28.8	62.5	54.3	17.7	22.0	31.6	10.1	39.7	34.3
To different mandal	86.8	55.9	89.5	75.7	83.2	68.5	74.1	33.0	96.3	93.5	89.5	74.9
Distance call to pick-up site* Km Median (IQR)	2(1-14)	12(6-18)	10(3-18)	12(6-19)	4(1-6)	9(2-17)	1(1-10)	12(7-18)	1(1-1)	11(6-18)	3(1-15)	12(6-20)
Distance pick-up site to hospital,* km Median (IQR)	28(18-40)	15(8-23)	21(12-31)	17(10-26)	16(5-30)	8(3-15)	24(14-37)	14(8-21)	32(21-48)	12(6-20)	29(19-43)	17(10-28)
Time call to pick-up site,* minutes ; Median (IQR)	11(7-28)	25(16-37)	24(12-37)	27(17-40)	15(7-33)	27(17-39)	10(7-19)	24(16-34)	12(8-27)	35(22-56)	12 (7-29)	26(16-39)
Time pick-up site to hosp,* minutes ; Median(IQR)	49(32-70)	26(16-40)	37(23-55)	32(20-46)	40(26-64)	21(13-32)	40(26-60)	23(14-34)	83(57-116)	43(28-65)	48 (33-70)	31(20-48)
Time call to hospital,* minutes ; Median (IQR)	77(56-102)	63(46-84)	75(55-97)	72(54-93)	70(50-97)	56(39-77)	64(47-86)	56(42-74)	105(72-143)	80(54-121)	77 (57-103)	70(52-93)

IFT= Inter-facilty transfer; IQR=Interquartile range; *N varies- excludes deliveries by EMT that were not transported or missing value

For peer review only

Determinants of Inter-facility transfer

Overall, women with obstetric emergencies transported by 108 were roughly twice as likely to have an IFT as women with no obstetric emergency (crude OR 2.25, 95% CI 2.16-2.34) (Table-5). In the adjusted analysis (excluding Chhattisgarh), obstetric emergencies had 1.95 (95% CI, 1.83–2.06) times higher odds of having IFT compared to non-emergencies (Table-5). Women from urban areas were twice as likely to have IFTs as women in rural areas (AOR 2.34, 95% CI 2.26 – 2.40). There was no evidence of an independent effect of high-priority districts on IFT. Overall, there was evidence of a 'J' shaped trend with maternal age. IFTs were marginally more likely in women less than 25, with a trend of increasing odds in women aged 30 or more, compared to women aged 25-30 years (AOR 1.01, 1.04, 1, 1.21, 1.43 respectively for age groups ≤19, 20-24, 25-30, 30-34 and ≥35 years). Although the effects were small, women from backward castes (AOR 0.95, 95% C.I. 0.92-0.99), scheduled castes (AOR 0.94, 95% C.I. 0.90-0.98), and scheduled tribes (AOR 0.98, 95% C.I. 0.95-1.00) were less likely to have IFT than women from general castes, after adjustment. Women from below the poverty line (AOR 0.92, 0.88-0.96) were less likely to have IFTs compared to women above the poverty line.

The determinants of IFT had different pattern of effect across states as shown in table-5. In respect to individual state models, the association between obstetric emergencies and IFT was strongest for Chhattisgarh (AOR 5.32, 95% CI 4.70-6.02) followed by Himachal Pradesh (AOR 3.03, 95% CI 2.65-3.47).

For peer review only - http://bmjopen.bmj.com/site/about/guidelines.xhtml

For peer review only

Table-5 Determinants of IFT across states (Logistic regression analysis showing Odds ratio (95% C.I.))

	Total*	Andhra Pradesh	Chhattisgarh	Gujarat	Himachal Pradesh	Telangana
**Bivariate logistic regression: OR (95% CI)**						
Obstetric emergency	2.25 (2.16 – 2.34)	1.81 (1.67 – 1.95)	5.39 (4.76 – 6.09)	2.01 (1.85 – 2.17)	3.15 (2.84 – 3.49)	1.90 (1.73 – 2.09)
Urban area	2.29 (2.23 – 2.34)	1.87 (1.80 – 1.94)	1.09 (0.92 – 1.29)	4.93 (4.69 – 5.20)	6.34 (5.68 – 7.07)	1.68 (1.60 – 1.76)
High priority district	0.96 (0.94 – 0.98)	1.10 (1.06 – 1.14)	1.28 (1.13 – 1.44)	0.69 (0.65 – 0.73)	0.92 (0.83 – 1.01)	0.95 (0.90 – 1.00)
Age group (years)						
≤19	1.06 (1.00 – 1.13)	1.08 (0.98 – 1.18)	1.37 (1.13 – 1.66)	1.28 (1.08 – 1.51)	0.70 (0.57 – 0.86)	1.01 (0.88 – 1.14)
20 – 24	1.03 (1.00 - 1.05)	1.02 (0.98 – 1.07)	1.09 (0.97 – 1.21)	1.10 (1.04 – 1.16)	0.87 (0.79 – 0.95)	1.02 (0.97 – 1.07)
25 – 30	1	1	1	1	1	1
30 – 34	1.24 (1.18 - 1.30)	1.29 (1.17 – 1.42)	1.38 (1.16 – 1.65)	1.25 (1.15 – 1.36)	1.08 (0.93 – 1.25)	1.21 (1.08 – 1.35)
≥35	1.52 (1.41 – 1.64)	1.62 (1.41 – 1.86)	1.68 (1.31 – 2.15)	1.58 (1.39 – 1.80)	1.16 (0.93 – 1.46)	1.47 (1.23 – 1.76)
Social caste						
General caste	1	1	1	1	1	1
Backward caste	0.92 (0.89 – 0.96)	0.95 (0.90 – 1.01)	1.04 (0.68 – 1.58)	0.94 (0.87 – 1.02)	1.55 (1.36 – 1.77)	0.71 (0.65 – 0.77)
Scheduled caste	0.91 (0.87 – 0.94)	1.02 (0.97 – 1.09)	0.91 (0.58 – 1.44)	0.87 (0.79 – 0.96)	0.89 (0.80 – 0.99)	0.67 (0.61 – 0.73)
Scheduled tribe	0.90 (0.87 – 0.94)	1.18 (1.11 – 1.28)	0.83 (0.54 – 1.27)	0.75 (0.69 – 0.81)	1.10 (0.93 – 1.31)	0.71 (0.65 – 0.78)
Below poverty line	0.92 (0.88 – 0.96)	1.45 (1.04 – 2.01)	0.92 (0.22 – 3.80)	0.93 (0.89 – 0.98)	0.83 (0.76 – 0.90)	1.03 (0.75 – 1.41)
**Multivariate logistic regression model: AOR (95% CI)****						
Obstetric emergency**	1.96 (1.83 – 2.06)	1.80 (1.65 – 1.95)	5.32 (4.70 – 6.02)	1.89 (1.74 – 2.05)	3.03 (2.65 – 3.47)	1.79 (1.61 – 1.98)
Urban area	2.35 (2.26 – 2.41)	1.86 (1.79 – 1.94)	1.09 (0.92 – 1.29)	4.75 (4.50 – 5.01)	5.76 (5.03 – 6.59)	1.64 (1.56 – 1.73)
High priority district	0.98 (0.95 – 1.00)	1.09 (1.05 – 1.13)	1.25 (1.11 – 1.41)	0.90 (0.85 – 0.96)	1.17 (1.03 – 1.33)	1.00 (0.95 – 1.06)
Age group (years)						
≤19	1.01 (0.94 – 1.06)	1.01 (0.92 – 1.11)	1.36 (1.12 – 1.65)	1.25 (1.06 – 1.49)	0.82 (0.65 – 1.05)	0.91 (0.80 – 1.05)
20 – 24	1.04 (0.98 – 1.06)	1.03 (0.99 – 1.08)	1.11 (1.00 – 1.24)	1.12 (1.06 – 1.18)	0.92 (0.82 – 1.03)	1.02 (0.97 – 1.07)
25 – 30	1	1	1	1	1	1
30 – 34	1.21 (1.14 – 1.27)	1.26 (1.14 – 1.39)	1.33 (1.11 – 1.59)	1.21 (1.11 – 1.32)	0.94 (0.77 – 1.14)	1.19 (1.07 – 1.34)
≥35	1.43 (1.35 – 1.56)	1.56 (1.36 – 1.80)	1.52 (1.19 – 1.95)	1.44 (1.27 – 1.64)	0.96 (0.72 – 1.30)	1.43 (1.19 – 1.71)
Social caste						
General caste	1	1	NA#	1	1	1
Backward caste	0.95 (0.92 – 0.99)	0.93 (0.87 – 0.99)	NA#	1.02 (0.94 – 1.10)	1.80 (1.55 – 2.10)	0.79 (0.71 – 0.87)

For peer review only - http://bmjopen.bmj.com/site/about/guidelines.xhtml

Scheduled caste	0.94 (0.90 – 0.98)	1.02 (0.95 – 1.08)	NA#	0.99 (0.90 – 1.09)	1.01 (0.90 – 1.14)	0.74 (0.61 – 0.73)
Scheduled tribe	0.98 (0.96 – 1.00)	1.22 (1.13 – 1.31)	NA#	0.94 (0.87 – 1.01)	1.31 (1.08 – 1.61)	0.82 (0.67 – 0.82)
Below poverty line	0.92 (0.88 – 0.96)	1.40 (1.00 – 1.96)	NA#	0.95 (0.90 – 1.00)	0.85 (0.77 – 0.95)	1.34 (0.95 – 1.88)
Pseudo R2	0.076	0.014	0.033	0.054	0.085	0.010

*adjusted for i.state and does not include State of Chhattisgarh; ** Adjusted for obstetric emergency, urban area, high priority district and age group, as appropriate; # for Chhattisgarh social caste and below poverty line were not included.

DISCUSSION

This is the first study assessing IFTs for pregnant women using the '108' ambulance service—the largest provider of the emergency medical services in India. We discuss findings with respect to patterns of use and the existing health system.

We estimated that '108' transferred around one fifth of all pregnancies and institutional deliveries in the five states. However, '108' service transported only 1% of all institutional deliveries in the population for IFTs. Only 1% of all institutional deliveries were transported by '108' for obstetric emergencies. The findings suggest that the '108' service is not a preferred choice for transport from lower-level facility to a higher-level facility, or for obstetric emergencies. Details about characteristics of obstetric emergencies transported by '108' ambulance services and discussion about coverage are published in another paper.[28]

The proportion of IFTs among all institutional deliveries will depend on the pattern of use of level of health care, referral practices and the availability of transport for between facility transfers. Roughly one-half of the non-IFTs went to peripheral birthing centres or basic EmOC centres in our study. A systematic review from India (including most studies from public health facilities) found that between 14% and 36% of all pregnancies were referred from nurse-run

For peer review only

delivery or basic EmOC centres, and a further 2% to 7% were referred from doctor- run basic EmOC centres for complications or emergencies.[13] Assuming the pattern of use of health facilities in our study and evidence from the review, we estimate that between 40,000 and 80,000 institutional deliveries who used '108' for non-IFTs may require further referral to higher facility. In addition, among the estimated 80% (2,300,000) institutional deliveries who went to their first facility by other means of transport, some women may be referred further. Thus the absolute numbers of pregnant women referred and requiring transport for IFT are likely to be large while '108' transports only about 35,000 pregnant women for IFT.

In our analysis, of all the transfers by '108' only 5.8% were IFTs—lowest 2.4% in Gujarat and highest 11.3% in Himachal Pradesh. The proportion of IFT was higher in States of Karnataka and Tamil Nadu (12.8% and 35.7%) among '108' users in 2013-14.[24] It appears that there is potential for increasing use of '108' for IFTs in the study States.

A study conducted in Andhra Pradesh in 2009, found that none of the pregnant women who used '108', was referred from a facility.[35] Some women did not prefer to wait for '108' if they perceived any emergency.[35] A maternal death review in Uttar Pradesh in 2010 found that only 5 of the 32 mothers who were transferred between facilities used an ambulance.[6] However, for other public transportation schemes, it was found that a high proportion (two-thirds) of all the inter-facility referrals in a study from Madhya Pradesh used Janani Express service (non-ambulance) while others used personal transport, taxis, autorikshaws or public transport.[10]

For peer review only - http://bmjopen.bmj.com/site/about/guidelines.xhtml

For peer review only

Although IFTs in our study were twice as likely to transport pregnant women who had any obstetric emergency compared to non-IFTs, there was a very large proportion of IFTs with no obstetric emergency or complication (92%). One of the '108' doctors, during discussion to understand IFT processes, mentioned that on many occasions they were not convinced of the need for IFT. However on insistence of the referring staff, the '108' doctor approved transport for IFT (personal communication). Often the referral was done because there was no doctor on duty or other resources were not available, as was also found in Madhya Pradesh.[10] These non-emergency IFTs will add unnecessary load at higher facilities and also make the ambulances unavailable for other emergencies.

In our study, IFTs had a higher overall proportion of women from disadvantaged social castes, below-the-poverty-line and urban areas compared to non-IFTs. Greater proportions of urban women deliver in health facilities,[30-32] and thus have a higher probability of early detection of complications and referral to a higher facility. Studies have also shown that higher proportions of women who use publically financed transportation schemes ('108'/'102'/Janani Express Yojana) belong to historically disadvantaged and backward social caste, below-the-poverty-line, are less educated and mostly from rural geographical areas compared to non-users.[21, 35-38]

The median time from call to '108' and reaching the destination facility was similar for non-IFT, and IFT, and it ranged from 1 hour to 1.3 hour. A study of 57 maternal death reviews from Uttar

For peer review only - http://bmjopen.bmj.com/site/about/guidelines.xhtml

Pradesh found much longer times; the mean time taken to arrange transport and travel from home to facility was about 4 hours, and transport from one facility to another was about 10 hours.[6] Although the '108' service has a mandate to inform the destination facility before arrival to reduce delays in treatment, this is not practiced as the list of contact points is not provided to EMTs.[Source: Dr. GVR Rao] Few women were transferred to a district different from the originating district due to sparse distribution of referral facilities. These women also travelled longer distances. Continuity of care and monitoring of IFTs can be better if transfers are in same administrative unit i.e. district.

The '108' ambulances are stationed close to CHCs and should be readily available, but if the ambulance is on route for pick-up or drop-off of another client then the IFT client with complications will have to wait or arrange for another means of transportation.[21] The '108' service may consider prioritising IFTs by having dedicated fleet of ambulances for IFTs over transporting all non-IFTs by increasing the fleet of ambulances stationed closer to beneficiaries.[25] In some states, the under-utilised ambulances at the health facilities are being used through the '102' call centre along with '108', however the distribution of these into IFT and non-IFT use has not been well laid out.[21] In other States, '102' has new ambulances that focus on IFT or transfers back to home for pregnant women.[26] Since 2013, state of Assam has a dedicated fleet of 450 ambulances for IFTs operating through the '102' call system. Of these, over half of all IFTs were for pregnant women in 2015-16.[Source: GVK EMRI annual statistics] This stresses the need for dedicated ambulances to deal with emergency IFTs, equipped with advanced life and obstetric support facilities. Other services like Janani Express

For peer review only - http://bmjopen.bmj.com/site/about/guidelines.xhtml

yojana, do not provide clinical support during transfer and are found to take longer times in transfer of pregnant women.[21, 25]

The role of the '108' service in improving care at the referring and referral facility, and its overall impact on maternal morbidity and mortality reduction, could not be estimated from our analysis nor has been reported in other studies. Nonetheless, the '108' service is accepted as an important and successful intervention to improve patient transportation for obstetric, medical and other emergencies.[21, 24, 26, 35, 37] A study from five states, showed that '108' ambulances provide pre-hospital stabilising care to all pregnant women, and delivery and post-delivery care to those delivered at home or on the ambulance.[39] However, a study from Punjab found that about half of '108' ambulances did not meet required standards for basic life support.[37] Monitoring of the quality of en-route care and referral systems is integral for better outcomes and monitoring of any transport intervention.[16, 21, 40]

There are a few limitations to our analysis. Details of the type of emergency in the '108' database was based on the doctor's report or the diagnoses by the EMTs for IFTs, and only the EMTs for non-IFTs. This information is thus subject to inter-observer variability and differential reporting. However, we cannot estimate if this would have led to over-estimation or under-estimation of the effect estimate. A very few women who used '108' for antenatal care may have used it again for delivery care, and would have been counted twice. Data for treatment given en-route, and doctors' notes on IFTs, were mostly not recorded in the '108' database and thus could not be studied. Details of the source hospital for IFTs were not available and thus

For peer review only - http://bmjopen.bmj.com/site/about/guidelines.xhtml

For peer review only

details of transfers between type of facilities could not be assessed. There is a possibility that few calls for IFT for the women residing in rural areas were wrongly recorded, as they called from the health facilities in urban areas. This would have contributed to higher proportion of urban women among IFTs. The '108' service from Chhattisgarh was taken over by the '102' service since October 2013 thus use of '108' may be under-estimated for this state. We had large proportions of missing information on social and economic status from two states. We considered that the missingness was not at random and was not associated with outcome. There is evidence that in such situations a complete case analysis, as we reported, is associated with negligible bias compared to a multiple imputation approach.[41]

CONCLUSION

Of all the estimated institutional deliveries in India, only a very small proportion (6%) made use of the '108' ambulance for transfer between facilities. Among '108' users for IFTs around 92% did not have any complication or emergency. After adjusting for confounding factors, IFTs were more likely for women with obstetric emergencies, more than 30 years of age and from urban areas. Pregnant women from socially disadvantaged castes, below poverty line and from high priority districts were less likely to have IFTs. Utilisation of the '108' service and its determinants varied across states. Primary research is required to understand variation in utilisation and to explore the potential of the '108' ambulance service to serve a higher proportion of women requiring IFTs, in particular those having obstetric emergencies. The '108' service would benefit by having a triage system to ensure that women with an obstetric emergency requiring an IFT are prioritised.

For peer review only - http://bmjopen.bmj.com/site/about/guidelines.xhtml

ACKNOWLEDGEMENTS

We acknowledge GVK-EMRI for sharing the data and allowing scientific study without any interference.

FUNDING

This work was supported by a Wellcome Trust Capacity Strengthening Strategic Award to the Public Health Foundation of India and a consortium of UK universities.

COMPETING INTERESTS

The authors have declared that no competing interests exist.

AUTHOR CONTRIBUTIONS

Conceived and designed the protocol: SS, PD, OC, GVR, GVS. Contributed in analysis plan: SS, PD, OC, LO, GVS. Analyzed and interpreted the data: SS. Contributed in preparation of manuscript: SS, PD, OC, LO, GVR, GVS.

ETHICS APPROVAL

The research obtained ethics approval from ethics committees of both LSHTM and IIPH-Hyderabad. (LSHTM Ethics Ref: 9613; IIPHH Ethics Ref: IIPHH/TRC/IEC/009/2014)

DATA SHARING

For peer review only

For peer review only - http://bmjopen.bmj.com/site/about/guidelines.xhtml

The data were obtained and analysed within the premises of GVK-EMRI office under license for the current study. The data are not publically available however can be available upon reasonable request directly to GVK-EMRI.

REFERENCES

1. National Rural Health Mission (NRHM). All India executive summary: status as on 31st March 2013. New Delhi: NRHM, Government of India 2013.

2. Registrar General of India. SRS: Maternal Mortality Ratio Bulletin 2011-2013. New Delhi: Government of India 2014. http://www.censusindia.gov.in/vital_statistics/mmr_bulletin_2011-13.pdf (accessed 30 September 2016).

3. WHO. Trends in maternal mortality: 1990 to 2013. Estimates by WHO, UNICEF, UNFPA, The World Bank and the United Nations Population Division. Geneva: WHO 2014. http://apps.who.int/iris/bitstream/10665/112682/2/9789241507226_eng.pdf (accessed 30 September 2016).

4. UNICEF. Levels & Trends in Child Mortality: Report 2015. Estimates Developed by the UN Inter-agency Group for Child Mortality Estimation United Nations Levels. New York: UNICEF 2015.

http://www.unicef.org/publications/files/Child_Mortality_Report_2015_Web_9_Sept_15.pdf (accessed 30 September 2016).

5. WHO. The world health report 2005: Make every mother and child count. Geneva: WHO 2005. http://www.who.int/whr/2005/whr2005_en.pdf?ua=1 (accessed 30 September 2016).

For peer review only - http://bmjopen.bmj.com/site/about/guidelines.xhtml

6. Raj SS, Manthri S, Sahoo PK. Emergency referral transport for maternal complication: lessons from the community based maternal death audits in Unnao district, Uttar Pradesh, India. *International Journal of Health Policy and Management* 2015;4:99-106.

7. Singh S, Murthy GVS, Thippaiah A, Upadhyaya S, Krishna M, Shukla R, et al. Community based Maternal Death Review: Lessons learned from ten districts in Andhra Pradesh, India. *Maternal and Child Health Journal* 2015;19(7):1447-54.

8. WHO, UNFPA, UNICEF, AMDD. Monitoring emergency obstetric care: a handbook. Geneva: WHO 2009. http://apps.who.int/iris/bitstream/10665/44121/1/9789241547734_eng.pdf (accessed 30 September 2016).

9. Murraya SF, Pearson SC. Maternity referral systems in developing countries: Current knowledge and future research needs. *Social Science & Medicine* 2006;62:2205-15.

10. Chaturvedi S, Randive B, Diwan V, De Costa A. Quality of Obstetric Referral Services in India's JSY Cash Transfer Programme for Institutional Births: A Study from Madhya Pradesh Province. *PLoS ONE* 2014;9(5):e96773. doi:10.1371/journal.pone.0096773.

11. David KV, Pricilla RA, Venkatesan S, Rahman SP, G S YK, Vijayaselvi R. Outcomes of deliveries in a midwife-run labour room located at an urban health centre: results of a 5-year retrospective study. *National Medical Journal of India* 2012;25(6):323-6.

12. Iyengar K, Iyengar SD. Emergency obstetric care and referral: experience of two midwife-led health centres in rural Rajasthan, India. *Reproductive Health Matters* 2009;17(33):9-20.

For peer review only - http://bmjopen.bmj.com/site/about/guidelines.xhtml

13. Singh S, Doyle P, Campbell OM, Mathew M, Murthy GVS. Referrals between public sector health institutions for women with obstetric high-risk, complications, or emergencies in India –A systematic review. *PLoS ONE* 2016;11(8):e0159793. doi:10.1371/journal.pone.0159793

14. Raj SS, Manthri S, Sahoo PK. Emergency referral transport for maternal complication: lessons from the community based maternal death audits in Unnao district, Uttar Pradesh, India. *International Journal of Health Policy and Management* 2015;4:99-106.

15. Giovine A, Ostrowski C. Technical report on Improving Transportation and Referral for Maternal Health: Knowledge Gaps and Recommendations. Washington DC: 2010. https://www.wilsoncenter.org/sites/default/files/Transport%20Referral%20Meeting%20Technical%20Report.pdf (accessed 30 September 2016).

16. UNICEF. Operating Perinatal Referral Transport Services in Rural India: A toolkit. New Delhi: UNICEF 2010. https://www.mailman.columbia.edu/sites/default/files/pdf/unicef_2010_operating_perinatal_transport_services_in_rural_india_toolkit.pdf (accessed 30 September 2016).

17. Thaddeus S, Maine D. Too far to walk: maternal mortality in context. *Social science & medicine* 1994;38(8):1091-110.

18. Hussein J, Kanguru L, Astin M, Munjanja S. The effectiveness of emergency obstetric referral interventions in developing country settings: a systematic review. *Plos medicine* 2012;9(7):e1001264. doi:10.1371/journal.pmed.1001264.

19. Ministry of Health and Family Welfare. Directory of Innovations Implemented in the health Sector. New Delhi: MOHFW, Government of India 2008.

For peer review only - http://bmjopen.bmj.com/site/about/guidelines.xhtml

http://planningcommission.nic.in/reports/genrep/health/Directory_of_innovations_march.pdf (accessed 30 September 2016).

20. Ministry of Health and Family Welfare. Operational Guidelines on Maternal and Newborn Health. New Delhi: MOHFW, Government of India 2010.

21. Sundararaman T, Chakraborty G, Nair A, Mokashi T, Ved R. Publicly Financed Emergency Response and Patient Transport Systems Under NRHM. New Delhi: Government of India 2012.

22. Ministry of Health and Family Welfare. Rural Health Statistics: 2014-15. New Delhi: Statistics Division, MOHFW, Government of India 2016. http://wcd.nic.in/sites/default/files/RHS_1.pdf (accessed 30 September 2016).

23. Kumutha J, Rao GVR, Sridhar BN, Vidyasagar D. The GVK EMRI maternal and neonatal transport system in India: A mega plan for a mammoth problem. *Seminars in Fetal & Neonatal Medicine* 2015;20(5).

24. Raj AX. Saving lives through rural ambulance services: Experiences from Karnataka and Tamil Nadu states, India. *Transport and Communications Bulletin for Asia and the Pacific*. 2014;84:52-66.

25. NHSRC. Emergency Medical servcie (EMS) in India: A concept paper. New Delhi: NRHM, MOHFW, Government of India 2012.

26. National Health Mission. ERS/Patient Transport Service: National Ambulance Service (NAS), MOHFW, Government of India 2015. http://nrhm.gov.in/nrhm-components/health-systems-strengthening/emri-patient-transport-service.html (accessed 30 September 2016).

27. GVK-EMRI. National footprints- Reaching unreachable Hyderabad, AP: GVK-EMRI 2015. http://www.emri.in/our-presence (accessed 30 September 2016).

For peer review only - http://bmjopen.bmj.com/site/about/guidelines.xhtml

28. Singh S, Doyle P, Campbell OM, Oakley L, Rao GVR, Murthy GVS. Transport of pregnant women and obstetric emergencies in India: An analysis of '108' ambulance service system data. BMC Pregnancy and Childbirth. 2016;16(299).

29. Ministry of Health and Family Welfare. Guidance note for implementation of RMNCH+A interventions in High Priority Districts. New Delhi: MOHFW, Government of India 2013.

30. IIPS. Himachal Pradesh: District level household and facility survey (2012-13) Mumbai: MOHFW, Government of India 2014. https://nrhm-mis.nic.in/DLHS4/Reports/Himachal%20Pradesh.pdf (accessed 30 September 2016).

31. IIPS. Telangana: District level household and facility survey (2012-13) Mumbai: MOHFW, Government of India 2014. https://nrhm-mis.nic.in/DLHS4/Reports/Telangana.pdf (accessed 30 September 2016).

32. IIPS. Andhra Pradesh: District level household and facility survey (2012-13) Mumbai: MOHFW, Government of India 2014. https://nrhm-mis.nic.in/DLHS4/Reports/Andhra%20Pradesh.pdf (accessed 30 September 2016).

33. NRHM. Formulas for calculating health and family planning indicators: NRHM, Government of India; 2008 [Available from: http://pbnrhm.org/formulas/health_indicators.pdf (accessed 10 February 2017).

34. Registrar General of India. Annual Health Survey 2012-13 Factsheet: Chhattisgarh. New Delhi: Ministry of Home affairs, Government of India 2014 http://www.censusindia.gov.in/vital_statistics/AHSBulletins/AHS_Factsheets_2012-13/FACTSHEET-Chhattisgarh.pdf (accessed 30 September 2016).

For peer review only - http://bmjopen.bmj.com/site/about/guidelines.xhtml

35. The Institute of Health Systems. A study of Emergency Response Services in Three Districts of Andhra Pradesh. New Delhi: NHSRC, Government of India 2011.

36. Prinja S, Aggarwal AK, Kaur M. Evaluation of Haryana Emergency Ambulance Services. New Delhi: PGIMER, Chandigarh & NHSRC, 2011.

37. Prinja S, Bahuguna P, Lakshmi PVM, Mokashi T, Aggarwal AK, Kaur M, et al. Evaluation of publicly financed and privately delivered model of emergency referral services for maternal and child health care in India. *Plos ONE*. 2014;9(10): e109911. doi: 10.1371/journal.pone.0109911.

38. Prinja S, Jeet G, Kaur M, Aggarwal AK, Manchanda N, Kumar R. Impact of referral transport system on institutional deliveries in Haryana, India. *Indian Journal of Medical Research* 2014;139:883-91.

39. Strehlow MC, Newberry JA, Bills CB, Min HE, Evensen AE, Leeman L, et al. Characteristics and outcomes of women using emergency medical services for thirdtrimester pregnancy-related problems in India: a prospective observational study. *BMJ Open* 2016;6:e011459. doi:10.1136/bmjopen-2016-011459.

40. Murray SF, Davis S, Phiri KR, Ahmed Y. Tools for monitoring the effectiveness of district maternity referral systems. *Health Policy and Planning*. 2001;16:353-61.

41. White IR, John B. Carlin. Bias and efficiency of multiple imputation compared with complete-case analysis for missing covariate values. *Statistics in medicine* 2010;29(28):2920-31.

For peer review only - http://bmjopen.bmj.com/site/about/guidelines.xhtml

STROBE 2007 (v4) Statement—Checklist of items that should be included in reports of *cross-sectional studies*

Section/Topic	Item #	Recommendation	Reported on page #
**Title and abstract**	1	(*a*) Indicate the study's design with a commonly used term in the title or the abstract	1,3,4
		(*b*) Provide in the abstract an informative and balanced summary of what was done and what was found	3,4
**Introduction**			
Background/rationale	2	Explain the scientific background and rationale for the investigation being reported	6-8
Objectives	3	State specific objectives, including any prespecified hypotheses	8
**Methods**			
Study design	4	Present key elements of study design early in the paper	9
Setting	5	Describe the setting, locations, and relevant dates, including periods of recruitment, exposure, follow-up, and data collection	9,10
Participants	6	(*a*) Give the eligibility criteria, and the sources and methods of selection of participants	9,10
Variables	7	Clearly define all outcomes, exposures, predictors, potential confounders, and effect modifiers. Give diagnostic criteria, if applicable	10-12
Data sources/ measurement	8*	For each variable of interest, give sources of data and details of methods of assessment (measurement). Describe comparability of assessment methods if there is more than one group	10
Bias	9	Describe any efforts to address potential sources of bias	12
Study size	10	Explain how the study size was arrived at	Universal sample
Quantitative variables	11	Explain how quantitative variables were handled in the analyses. If applicable, describe which groupings were chosen and why	11,12
Statistical methods	12	(*a*) Describe all statistical methods, including those used to control for confounding	11,12
		(*b*) Describe any methods used to examine subgroups and interactions	12
		(*c*) Explain how missing data were addressed	12
		(*d*) If applicable, describe analytical methods taking account of sampling strategy	NA
		(*e*) Describe any sensitivity analyses	12
**Results**			

For peer review only

Participants	13*	(a) Report numbers of individuals at each stage of study—eg numbers potentially eligible, examined for eligibility, confirmed eligible, included in the study, completing follow-up, and analysed	12,13
		(b) Give reasons for non-participation at each stage	13
		(c) Consider use of a flow diagram	NA
Descriptive data	14*	(a) Give characteristics of study participants (eg demographic, clinical, social) and information on exposures and potential confounders	15,16
		(b) Indicate number of participants with missing data for each variable of interest	16,17, Table-3
Outcome data	15*	Report numbers of outcome events or summary measures	14-16,
Main results	16	(*a*) Give unadjusted estimates and, if applicable, confounder-adjusted estimates and their precision (eg, 95% confidence interval). Make clear which confounders were adjusted for and why they were included	15-19
		(*b*) Report category boundaries when continuous variables were categorized	15-19
		(*c*) If relevant, consider translating estimates of relative risk into absolute risk for a meaningful time period	15-19
Other analyses	17	Report other analyses done—eg analyses of subgroups and interactions, and sensitivity analyses	20-22
**Discussion**			
Key results	18	Summarise key results with reference to study objectives	23-26
Limitations	19	Discuss limitations of the study, taking into account sources of potential bias or imprecision. Discuss both direction and magnitude of any potential bias	27
Interpretation	20	Give a cautious overall interpretation of results considering objectives, limitations, multiplicity of analyses, results from similar studies, and other relevant evidence	27
Generalisability	21	Discuss the generalisability (external validity) of the study results	28
**Other information**			
Funding	22	Give the source of funding and the role of the funders for the present study and, if applicable, for the original study on which the present article is based	29

*Give information separately for cases and controls in case-control studies and, if applicable, for exposed and unexposed groups in cohort and cross-sectional studies.

Note: An Explanation and Elaboration article discusses each checklist item and gives methodological background and published examples of transparent reporting. The STROBE checklist is best used in conjunction with this article (freely available on the Web sites of PLoS Medicine at http://www.plosmedicine.org/, Annals of Internal Medicine at http://www.annals.org/, and Epidemiology at http://www.epidem.com/). Information on the STROBE Initiative is available at www.strobe-statement.org.

For peer review only

BMJ Open

Inter-facility transfer of pregnant women using publicly-funded emergency call-centre based ambulance services: A cross-sectional analysis of service logs from five states in India

Journal:	*BMJ Open*
Manuscript ID	bmjopen-2016-015077.R2
Article Type:	Research
Date Submitted by the Author:	02-May-2017
Complete List of Authors:	Singh, Samiksha; Indian Institute of Public Health-Hyderabad, PHFI<br>Doyle, Pat ; London School of Hygiene and Tropical Medicine, Faculty of Epidemiology and Population Health, Department of Non-communicable Disease Epidemiology<br>Campbell, Oona; London School of Hygiene and Tropical Medicine, Faculty of Epidemiology and Population Health, Department of Infectious Disease Epidemiology<br>Oakley, Laura; London School of Hygiene and Tropical Medicine, Faculty of Epidemiology and Population Health, Department of Non-communicable Disease Epidemiology<br>Rao, G.V. Ramana; GVK Emergency Management and Research Institute, Emergency Medicine Learning Center and Research<br>Murthy, GVS; Indian Institute of Public Health, Hyderabad, PHFI, Director; London School of Hygiene and Tropical Medicine, Faculty of Infectious and Tropical Diseases, Department of clinical research
**Primary Subject Heading**:	Public health
Secondary Subject Heading:	Health services research, Public health, Epidemiology, Obstetrics and gynaecology
Keywords:	Maternal medicine < OBSTETRICS, Ambulance, Inter-facility transfer, Obstetric emergency, Obstetric complication, Patient transport

SCHOLARONE™ Manuscripts

Title: Inter-facility transfer of pregnant women using publicly-funded emergency call-centre based ambulance services:A cross-sectional analysis of service logs from five states in India

Short title: Inter-facility transfer of pregnant women in India using 108 ambulances

Samiksha Singh, Pat Doyle, Oona M R Campbell, Laura Oakley, GVR Rao, GVS Murthy

Samiksha Singh, (Corresponding author)

Assistant Professor,

Indian Institute of Public Health-Hyderabad, Public Health Foundation of India

Address- Plot#1 AVN Arcade, Amar Co-operative society, Kavuri Hills, Madhapur, Hyderabad, Telanagana, India - 201010; Email- samiksha.singh@iiphh.org; Phone- +91 8886 111 290

Pat Doyle

Professor,

Department of Non-communicable disease epidemiology, Faculty of Epidemiology and Population Health, London School of Hygiene and Tropical Medicine, London, UK

Oona M Campbell

Professor,

Department of Infectious Disease Epidemiology, Faculty of Epidemiology and Population Health, London School of Hygiene and Tropical Medicine, London, UK

For peer review only

For peer review only - http://bmjopen.bmj.com/site/about/guidelines.xhtml

Laura Oakley

Assistant Professor,

Department of Non-communicable disease epidemiology, Faculty of Epidemiology and Population Health, London School of Hygiene and Tropical Medicine, London, UK

GV Ramana Rao

Director,

Emergency Medicine Learning Center and Research, GVK-Emergency Management and Research Institute, Hyderabad, India

GVS Murthy

Professor,

Indian Institute of Public Health-Hyderabad, Public Health Foundation of India, Hyderabad, India and Department of clinical research, Faculty of Infectious and Tropical Diseases, London School of Hygiene and Tropical Medicine, London, UK

Word count: 4630

Key words: Ambulance, Inter-facility transfer, Obstetric emergency, Obstetric complication, Pregnant women, Patient transport, Access, Maternity Services, Travel time

For peer review only

For peer review only - http://bmjopen.bmj.com/site/about/guidelines.xhtml

ABSTRACT

Objective: To estimate the proportion of Inter-facility transfers (IFTs) transported by '108' ambulances and to compare the characteristics of the IFTs and non-IFTs to understand the pattern of use of '108' services for pregnant women in India.

Design: A cross-sectional analysis of '108' ambulance records from five states for the period April 2013 to March 2014. Data were obtained from the call-centre database for the pregnant women, who called '108'.

Main outcomes: Proportion of all pregnancies and institutional deliveries in the population who were transported by '108', both overall and for IFT. Characteristics of the women transported; obstetric emergencies, the distances travelled, and the time taken for both IFT and non-IFT.

Results: The '108' ambulances transported 608,559 pregnant women, of whom 34,993 were IFTs (5.8%) in the five states. We estimated that '108' transferred 16.5% of all pregnancies and 20.8% of institutional deliveries. Only 1.2% of all institutional deliveries in the population were transported by '108' for IFTs—lowest 0.6% in Gujarat and highest 3.0% in Himachal Pradesh. Of all '108' IFTs, only 8.4% had any pregnancy complication. For all states combined, on adjusted analysis, IFTs were more likely than non-IFTs to be for older and younger women or from urban areas, and less likely to be for women from high-priority districts, from backward or scheduled castes, or women below-the-poverty-line. Obstetric emergencies were more than twice as likely to be IFTs as pregnant women without obstetric emergencies (OR=2.18, 95% CI, 2.09-2.27). There was considerable variation across states.

For peer review only - http://bmjopen.bmj.com/site/about/guidelines.xhtml

Conclusion: Only 6% institutional deliveries made use of the '108' ambulance for IFTs in India. The vast majority did not have any complication or emergency. The '108' service may need to consider strategies to prioritise the transfer of women with obstetric emergency and those requiring IFT, over uncomplicated non-IFT.

For peer review only

For peer review only - http://bmjopen.bmj.com/site/about/guidelines.xhtml

STRENGTHS & LIMITATIONS

- This study is the first to assess the role of the '108' ambulance service - the largest provider of the free emergency medical services in India – for IFTs of pregnant women.
- We assessed the characteristics of pregnant women who were transported by '108' as IFTs and compared them with those who were transported by '108' as non-IFTs, for five state.
- We did extensive cleaning and management of data to drive appropriate information.
- '108' service did not record information on post-partum obstetric emergency separately thus these could not be estimated.
- Diagnosis of obstetric emergency may be inaccurate and subject to inter-observer bias.
- The '108' database mostly did not record data for treatment given en-route, and doctors' notes on IFTs, thus we could not study these data.
- Some population estimates are based on assumptions and may not be accurate.
- We had large proportions of missing information on social and economic status from two states. We did a complete analysis and sensitivity analysis to deal with missingness.

For peer review only

For peer review only - http://bmjopen.bmj.com/site/about/guidelines.xhtml

BACKGROUND

India had an estimated 83% women delivering in health facilities in 2013.[1] Despite this high proportion of institutional births, India had an estimated maternal mortality ratio (MMR) of 167/ 100,000 live births and an early neo-natal mortality rate of 28/1000 live births in 2013.[2] The country accounts for 17% (50,000) of global maternal deaths per year[3] and 26% (696,000) of global neonatal deaths.[4] Many maternal deaths occur during transit to health facilities.[5-7]

Inter-facility transfers (IFTs) for pregnant women are crucial, especially in resource poor countries where most peripheral health facilities provide only uncomplicated birthing or basic emergency obstetric care.[8,9] About 14% to 36% of women delivering in facilities are referred from lower to higher-level facilities.[10-13] As IFT is more likely to be due to referral for high risk pregnancies or complications during pregnancy, childbirth or post-partum, it can play a pivotal role in reduction of maternal morbidity and mortality.[14, 15]

Ensuring an uneventful IFT is part of good health care provision at the referring facility, and will reduce delays in access to appropriate health care (delay type-2) and delays in getting appropriate care after reaching a health facility (delay type-3).[16, 17] A good process will include prompt arrangement of transport, en-route stabilisation, communication with the referral facility to prepare them for the patient, and appropriate hand-over on arrival. IFT is thus a complex coordinated effort made by the referring health care provider, the en-route attendant, the receiver at the referral health facility, and the referral transport system.[16] Several successful interventions have been reported in resource-poor countries to improve

For peer review only

For peer review only - http://bmjopen.bmj.com/site/about/guidelines.xhtml

referral transportation, but only a few mention IFT.[18, 19] Referral transport for IFT may be a) an ambulance based at the referring facility, b) an ambulance called from a referral facility, c) an independent ambulance service, d) other subsidised public or commercial transport, or e) personal or commercial (non-subsidised) transport.[16, 20]

India, currently, does not have any structured inter-facility referral and transportation protocols for pregnant women (desk review and personal communication with Maternal health specialist, Government of India). In India, most peripheral health facilities do not have functional ambulances of their own and if they do, they are not available round the clock.[14, 21, 22] Thus IFT for pregnant women depends on other referral transport services, the majority using the free, public funded '108'/'102' call centre based ambulance services[19, 23, 24] and paid subsidised, commercial, or personal transport. There are other services across states that, unlike '108', are basic patient transportation services with no en-route stabilisation care. These may also provide transport between facilities.[21, 25, 26] For example, Janani Express yojana (public private non-ambulance transport service), operating through call-centres within districts, transfers exclusively pregnant women and newborns in Madhya Pradesh and Odisha.[21, 25] In larger cities, some other ambulance services (free or paid) are also available.

There have been very few research studies on transportation for IFTs of pregnant or postpartum women in India, despite the large investments made in public referral transport. This study was conducted to investigate IFTs of pregnant women in India using '108' ambulance services. The objectives of the research were to i) estimate proportion of women transferred by

For peer review only - http://bmjopen.bmj.com/site/about/guidelines.xhtml

‘108’ among all institutional deliveries in the general population, ii) estimate the proportion of IFTs transferred by ‘108’ among all institutional deliveries in the population, iii) estimate the proportion of IFTs among all transfers of pregnant women by ‘108’, and iii) compare the characteristics of the IFTs and non-IFTs.

METHODS

Context

The ‘108’ ambulance service operates under a public private partnership. It operates 7361 ambulances, and transfers any medical emergency across 21 states and union territories (smaller less populated administrative units in India).[26] GVK-EMRI is the largest service provider for ‘108’ and operates in 15 states and 2 union territories. In 2014-15, GVK-EMRI ‘108’ ambulances transferred about 3.6 million pregnant women to health facilities, which were about two-fifths of all the transfers by GVK-EMRI ‘108’.[23] There is approximately one ambulance for every 100,000 population, and the ambulance should be well equipped and accompanied by an Emergency Medicine Technician (EMT). The EMT is trained to provide emergency care and basic life support in obstetrics. In cases of imminent childbirth, the EMT is expected to assist the delivery en-route and transfer the mother and child to the nearest health facility.[27] For IFTs, the EMT consults the ‘108’ call-centre based medical officer and the referring health care provider to discuss indication of referral, stability of case to withstand travel, stabilising care required and place of referral [Source: Expert from GVK-EMRI). ‘108’ service preferably transfers mother to public facilities, however in absence of appropriate public facility nearby, they transfer the mother to empaneled private facility.[27]

For peer review only - http://bmjopen.bmj.com/site/about/guidelines.xhtml

Working definitions

Inter-facility transfer (IFT), for this study, was defined as any transfer of a pregnant woman from one health facility to another health facility on the advice of a health care provider, using a '108' ambulance. All other transfers of pregnant women to health facilities using '108' ambulances were defined as 'non-IFT'. These mostly included transfers from home to facility.

An obstetric emergency was defined as any life threatening medical complication in women in pregnancy, labour or childbirth, or after (within 42 days of termination of pregnancy). For IFT women diagnosis of obstetric emergency was made by the referring doctor and noted by EMT after discussion with call-centre based doctor. For non-IFT, diagnosis was made by EMT. We observed that even high-risk such as previous caesarean section and precious pregnancy were considered obstetric emergency by '108' services thus we included them.[28]

Study Design

This cross-sectional study analysed '108' ambulance records from five states. Ethical approval for the study was obtained from Indian Institute of Public Health-Hyderabad and London School of Hygiene and Tropical Medicine.

Study population

Pregnant women who called '108' or for whom a relative or friend or health care provider called on their behalf, between 1st April 2013 and 31st March 2014 in the five states in India where GVK-EMRI operated '108' service had been fully functional for more than 3 years were

For peer review only - http://bmjopen.bmj.com/site/about/guidelines.xhtml

included in this analysis (Andhra Pradesh, Chhattisgarh, Gujarat, Himachal Pradesh, and Telangana). The period 1st April to 31st March was aligned with reporting period of state governments. States were chosen in a manner that they had representation of North, South, East and West of India.

Obtaining data and data management

Formal permission to use the data was obtained from GVK-EMRI. Anonymised information on '108' calls from 1st April 2013 to 31st March 2014 was obtained from the GVK-EMRI emergency response centre database. Details on data management are described in another paper form the same dataset.[28] Data were extracted onto Excel sheets, and converted to STATA 10.0 files. Data were inspected to assess consistency, range, and missing data. Any gross issue relating to the quality of records was noted, and records with improbable entries were excluded from analysis. Variables were recoded wherever needed. Variables of interest were: IFT; age of pregnant woman; social caste (general, other backward, schedule tribe and scheduled caste); economic class (below or above poverty line); area (rural or urban); type of emergency; time of call; day of call; time taken by ambulance to reach the client; time taken to reach the health facility; and distance travelled. Castes are classified based on historically backward and deprived social castes. The information was provided by the caller and EMT, and recorded in the call-centre database. Districts within states were stratified into high priority districts and non-high priority districts. High priority districts are those identified by the Government of India as being in the lowest quartile of districts (or tribal districts) in each state

For peer review only - http://bmjopen.bmj.com/site/about/guidelines.xhtml

with respect to maternal and child health indicators (including institutional delivery rate, maternal mortality and neonatal mortality rates among a total of 16 indicators).[29]

Analysis

We used information from the Census 2011 , District level household survey (DLHS) 2012-13,[30-32] and Annual health survey (AHS) 2012-13,[33] to estimate the number of pregnancies and institutional deliveries in the population for the study states, as mentioned below. The numbers of pregnant women transported by '108' as recorded in the call-centre database were compared with these population estimates for each state.

For each state, the number of pregnancies in the study period was estimated as sum of estimated pregnancies in rural and urban population [population (rural) X crude birth rate (rural) X 1.1 X 1000] + [population (urban) X crude birth rate (urban) X 1.1 X 1000]. The population data were obtained from the 2011 census and the crude birth rates from the Sample Registration System 2013. The multiplier 1.1 is used to account for an estimated 10% of the pregnancies which may have ended in abortions or intra-uterine deaths.[34]

The number of institutional deliveries in the study period was estimated as [Estimated number of pregnancies (rural) X institutional delivery rate (rural) X 100] + [Estimated number of pregnancies (urban) X institutional delivery rate (urban) X 100]; Institutional delivery rates include live births and still-births. The institutional delivery rate were obtained from DLHS-4 and AHS-2 surveys.

For peer review only - http://bmjopen.bmj.com/site/about/guidelines.xhtml

Information was analysed for all states combined and separately by State, comparing IFT and non-IFT. The characteristics of the women transported, distances travelled and the time taken by '108' ambulances, were described for both IFT and non-IFT journeys. The association between socio-demographic and clinical variables and the outcome (IFT vs no-IFT) was investigated using bivariate and multivariate logistic regression.

Social caste and economic class were missing in 55% and 95% of observations in the state of Chhattisgarh, and 14% and 22% in Himachal Pradesh. Given the nature of the variables we decided that it was likely that the data were missing not at random, and therefore multiple imputation was considered inappropriate. In order to investigate possible selection bias resulting from missing data, we performed a complete case analysis for each state and all states combined, supplemented by a series of sensitivity analyses. Our first sensitivity analysis involved running a model with and without states of Chhattisgarh and Himachal Pradesh. Presence or absence of these states did not substantially change the pattern of results. The second sensitivity analysis was performed by running a model with and without social caste and economic class for all States combined (total) and for individual states. The presence or absence of these variables did not substantially change the magnitude of the pattern of results (Odds ratios and Rsq for each model) for the total and for states (except Chhattisgarh). For our main analysis (all States combined) we thus included social caste and economic class variables and excluded any data from Chhattisgarh. Models for individual states (except Chhattisgarh) also include social caste and economic class.

For peer review only

For peer review only - http://bmjopen.bmj.com/site/about/guidelines.xhtml

RESULTS

Study Populations

The study states had population sizes ranging from 6 million to 60 million and had different social compositions (Table-1). The percentage of rural population ranged from 61% in Telangana to 90% in Himachal Pradesh while scheduled castes and tribes, together, ranged from 22% in Andhra Pradesh and Gujarat to 43% in Chhattisgarh. The crude birth rate was lowest in Himachal Pradesh (1.6%) and highest in Chhattisgarh (2.5%). The states also varied in the institutional delivery rates from 39.5% in Chhattisgarh to 94.1% in Telangana, and in their MMRs from 244 in Chhattisgarh to 92 per 100,000 live births in Telangana and Andhra Pradesh. On an average '108' ambulance is sanctioned for approximately 100,000 population in all states except Himachal Pradesh where one ambulance caters to about 40,000 population. Himachal Pradesh is mostly hilly and has sparse population distribution and '108' ambulances are stationed based on geographic regions.

Table 1: Demographic characteristics of the study states

	Andhra Pradesh	Chhattisgarh	Gujarat	Himachal Pradesh	Telangana
Total Population*	49,386,799	25,540,196	60,383,628	6,856,509	35,193,978
Rural/tribal	70.4%	76.8%	57.4%	90.0%	61.3%
Urban	29.6%	23.2%	42.6%	10.0%	38.7%
Scheduled caste*	17.1%	12.8%	6.8%	25.2%	15.4%
Scheduled tribe*	5.3%	30.6%	14.8%	5.7%	9.3%
Crude birth rate per 1000 population**	17.5	24.5	21.1	16.2	17.5
Institutional delivery rate per 100 childbirth***	88.5%	39.5%	78.1%	77.8%	94.1%
MMR per 100,000 livebirths (2011-13)**	92	244	112	Not available	92
NMR per 1,000 livebirths (2012-13)**	25	31	26	25	25

For peer review only - http://bmjopen.bmj.com/site/about/guidelines.xhtml

No. of Ambulances under ‘108’[#]	468	240	506	171	334
Geographic region/terrain	Tribal pockets	Large Tribal; hilly pockets	Tribal pockets	Majority hilly	Tribal pockets

Source- *Census 2011; ** Sample Registration System 2013- Separate data for Telangana and Andhra Pradesh not available; *** DLHS (2012-13) / AHS (2011-12); [#] GVK-EMRI annual report for period April 2013- March 2014. [$]In Chhattisgarh ‘102’ ambulance service took over from October 2013 – March 2014.

For peer review only

Description of 108 calls relating to pregnancy

Across the five states, ‘108’ call centres received 646,656 calls for pregnancy-related transfers, 6.2% of which were for IFT. Among IFT calls, an ambulance was not assigned to 1.2%, and an ambulance was assigned but not used by 6.0% of callers. Among non-IFT callers these proportions were 1.4% and 3.9% respectively. A total of 608,559 pregnant women were transported using ‘108’, and of these journeys 5.8% were for IFT.

Estimated proportions of pregnancies, institutional deliveries and IFTs transported by ‘108’

Table-2 presents estimates of the number of pregnant women, obstetric emergencies, institutional deliveries and IFTs in the general populations, and the proportion of these transported using ‘108’ ambulances in the five states, and overall. The 608,559 pregnant women transferred comprised 16.5% of all estimated pregnancies, and 20.8% of all estimated institutional deliveries for the study states combined.

Of the estimated institutional deliveries, ‘108’ transported only 1.2% (34,993) women between facilities across States. This proportion was highest in Himachal Pradesh (3.0%) followed by

For peer review only - http://bmjopen.bmj.com/site/about/guidelines.xhtml

Andhra Pradesh (1.7%), Telangana (1.4%), Chhattisgarh (0.7%) and lowest in Gujarat (0.6%). Only 1.0% (28,448) of all institutional deliveries were transported by '108' for obstetric emergencies—highest 2.4% in Himachal Pradesh and lowest 0.6% in Andhra Pradesh and Telangana (Table-2).

Table 2: Estimated proportion of pregnant women transported to hospitals by '108' from April 2013 to March 2014

	Total	Andhra Pradesh	Chhattisgarh [$]	Gujarat	Himachal Pradesh	Telangana
Estimated number of pregnancies in the population [a]	3,811,920	950,696	678,217	1,387,021	121,638	673,508
Estimated number of institutional deliveries in the population [b]	2,921,674	842,109	267,896	1,083,264	94,634	633,771
Proportion of all pregnancies transported by '108' [c]	16.5%	15.5%	9.0%	19.5%	20.5%	15.7%
Proportion of all institutional deliveries transported by '108' [d]	20.8%	17.5%	22.7%	24.9%	26.3%	16.6%
Proportion of all institutional deliveries that were transported by '108' for inter-facility transfers [e]	1.2%	1.7%	0.7%	0.6%	3.0%	1.4%
Proportion of institutional deliveries that were transported by '108' for obstetric emergencies [f]	1.0%	0.6%	1.0%	1.4%	2.4%	0.6%

[a] [population (rural) X crude birth rate (rural) X 1.1 X 1000] + [population (urban) X crude birth rate (urban) X 1.1 X 1000]; Using Census populations and crude birth rates from Sample Registration Survey in respective states

[b] [Estimated number of pregnancies (rural) X institutional delivery rate (rural) X 100] + [Estimated number of pregnancies (urban) X institutional delivery rate (urban) X 100]; Institutional delivery rates include live births and still births. Using institutional delivery rates from District Level Household Survey / Annual Household Survey in respective states

[c] Number of pregnant women transported by '108'/Estimated number of pregnancies in the population([a])

[d] Number of pregnant women transported by '108'/Estimated number of institutional deliveries in the population([b])

[e] Number of pregnant women transported between facilities (IFTs) by '108'/ estimated number of institutional deliveries in the population([b])

[f] Number of pregnant women transported by '108' for obstetric emergency / estimated number of institutional deliveries in the population([b])

[$]In Chhattisgarh '102' ambulance service took over from '108' service from October 2013 to March 2014. Thus number of beneficiaries of '108' and proportions may be underestimation of utilization for annual estimates.

Characteristics of pregnant women transferred by '108' ambulances

For peer review only - http://bmjopen.bmj.com/site/about/guidelines.xhtml

The pregnant women who were transferred by '108' belonged mostly to lower social and economic sections- rural or tribal areas (84.6%) or scheduled castes or tribes (64.9%) and below-the-poverty-line status (76.6%). Two hundred and forty two women (40 per 100,000) died before the ambulance reached the pick-up site. The proportion of pregnant women who died before arrival of '108' ambulance was higher in Chhattisgarh (150 per 100,000) compared to other states.

Of the pregnant women transferred by '108' ambulances, 34,993 (5.8%) had an IFT (Table-3). The proportion of IFTs among women transported by '108' was highest in Himachal Pradesh (11.3%) followed by Andhra Pradesh (9.9%), Telangana (8.7%), Chhattisgarh (3.2%) and Gujarat (2.4%).

Overall, IFTs were made up of a higher proportion of younger women, women from backward caste, belonging to below-the-poverty-line and from urban areas compared to non-IFTs. A Similar pattern was observed across all the states except in Himachal Pradesh where IFTs had lower proportion of women from below-the-poverty-line strata compared to non-IFTs (Table-3). Delivery en-route or in the ambulance was less likely among IFTs compared to non-IFTs across all states.

For peer review only - http://bmjopen.bmj.com/site/about/guidelines.xhtml

Table 3: Demographic characteristics of users of '108' ambulance service for IFT and non-IFT in states (April 2013 to March 2014)

	Total		Andhra Pradesh		Chhattisgarh		Gujarat		Himachal Pradesh		Telangana	
	IFT (N=34,993) [5.8%]	Non-IFT (N=573,566) [94.2%]	IFT (N=14,574) [9.9%]	Non-IFT (N=132,800) [90.1%]	IFT (N=1,929) [3.2%]	Non-IFT (N=58,881) [96.8%]	IFT (N=6,562) [2.4%]	Non-IFT (N=263,509) [97.6%]	IFT (N=2,809) [11.3%]	Non-IFT (N=22,114) [88.7%]	IFT (N=9,119) [8.7%]	Non-IFT (N=96,262) [91.3%]
Age group (years)												
<u><</u>19	4.1	3.4	5.0	4.8	7.1	5.8	2.3	2.0	4.4	5.7	3.5	3.6
20 – 24	58.7	56.5	64.9	65.7	54.9	56.4	52.0	51.9	48.3	51.6	58.6	58.9
25 – 30	28.1	30.6	24.0	24.9	25.0	27.9	30.1	33.2	34.5	32.0	31.4	32.2
30 – 34	6.5	7.2	4.2	3.3	8.9	7.2	11.3	9.9	9.4	8.0	4.8	4.1
<u>></u>35	2.6	2.3	1.9	1.2	4.1	2.7	4.3	3.0	3.4	2.7	1.7	1.2
Total non-missing	(32,538) 100	(551,568) 100	(13,105) 100	(120,155) 100	(1,914) 100	(58,591) 100	(6,562) 100	(263,508) 100	(2,779) 100	(21,694) 100	(8,178) 100	(87,620) 100
missing	(2,455) 7.0	(21,988) 3.8	(1,469) 10.1	(12,645) 9.5	(15) 0.8	(290) 0.5	(0) 0	(1) 0	(30) 1.1	(420) 1.9	(941) 10.3	(8,642) 9.0
Social caste												
General caste	12.2	11.3	10.8	10.9	3.7	3.5	14.8	12.8	37.3	38.2	7.1	5.0
Other backward	40.0	38.5	41.7	44.1	48.5	43.8	38.3	35.0	17.4	11.5	43.4	43.5
Scheduled caste	29.1	22.1	34.7	34.1	14.3	14.7	11.2	11.1	37.2	42.8	32.2	34.2
Scheduled tribe	18.7	28.2	12.8	10.9	33.6	38.1	35.8	41.2	8.1	7.6	17.3	17.3
Total non-missing	(32,800) 100	(535,328) 100	(14,400) 100	(131,924) 100	(652) 100	(27,427) 100	(6,551) 100	(263,204) 100	(2,201) 100	(17,148) 100	(8,996) 100	(95,625) 100
DK/ missing	(2,193) 6.3	(38,238) 6.7	(174) 1.2	(876) 0.7	(1,277) 66.2	(31,454) 53.4	(11) 0.2	(305) 0.1	(608) 21.6	(4,966) 22.5	(123) 1.3	(637) 0.7
Economic class												
BPL	87.7	75.9	99.7	99.6	96.7	97.0	54.2	55.9	62.7	67.0	99.5	99.5
Others	12.3	24.1	0.3	0.4	3.3	3.0	45.7	44.1	37.3	33.0	0.5	0.5
Total non-missing	(32,296) 100	(511,447) 100	(14,357) 100	(131,391) 100	(61) 100	(2824) 100	(6,540) 100	(262,991) 100	(2,395) 100	(19,041) 100	(8,943) 100	(95,200) 100
DK/ missing	(2,697) 7.7	(62,119) 10.8	(217) 1.5	(1,409) 1.1	(1,868) 96.8	(56,057) 95.2	(22) 0.3	(518) 0.2	(414) 14.7	(3,073) 13.9	(176) 1.9	(1,062) 1.1
Area												
Rural/tribal	68.7	85.6	67.4	79.5	92.1	92.7	62.8	89.3	75.9	95.2	67.6	77.8
Urban	31.3	14.4	32.6	20.5	7.9	7.3	37.2	10.7	24.1	4.8	32.4	22.2
Total non-missing	(34,910) 100	(570,042) 100	(14,555) 100	(132,510) 100	(1,929) 100	(58,881) 100	(6,562) 100	(263,509) 100	(2,770) 100	(18,952) 100	(9,094) 100	(96,190) 100
Missing	(78) 0.2	(3,524) 0.6	(19) 0.1	(290) 0.2	(0) 0	(0) 0	(0) 0	(0) 0	(39) 1.4	(3,162) 14.3	(25) 0.3	(72) 0.1
Died before ambulance reached	0.07	0.04	0.04	0.02	0.36	0.14	0.02	0.03	0	0.01	0.02	0.02
Delivery en-route												
At pick up site	0.3	1.4	0.2	0.8	1.2	2.8	0.5	1.7	0.2	1.7	0.2	0.7
In ambulance	0.6	1.0	0.4	0.7	0.8	1.5	0.6	0.9	1.7	3.5	0.4	0.7

IFT= Inter-facility transfer ; non-IFT = non-inter-facility transfer

For peer review only

For peer review only - http://bmjopen.bmj.com/site/about/guidelines.xhtml

Obstetric emergencies among women transferred by '108' ambulance for IFT and non-IFT

The majority of transfers by '108' ambulances were for normal labour. By state, between 2.7% and 9.3% of transferred pregnant women had an obstetric emergency; the overall average was 4.8%. The IFTs (8.4%) had higher proportion of pregnant women who had an obstetric emergency compared to non-IFTs (4.4%) (Table-4). Himachal Pradesh had highest proportion of obstetric emergencies transferred for both IFT (21.0%) and non-IFT (7.8%). Andhra Pradesh was the lowest proportion of obstetric emergencies transferred for IFT (5.4%) and non-IFT (3.1%).

The most common obstetric emergencies were 'abnormal presentation of foetus' and 'bleeding in pregnancy' in Telangana, Andhra Pradesh and Gujarat; and 'bleeding in pregnancy' and 'medical conditions complicating pregnancy' in Himachal Pradesh and Chhattisgarh. IFTs had higher proportions of 'bleeding in pregnancy' cases across all the states and 'medical conditions complicating pregnancy' in Himachal Pradesh and Chhattisgarh (Table-4). In Telangana and Andhra Pradesh, some IFTs (0.5% and 0.2%) and non-IFTs (8.43% and 0.53%) were for antenatal women requiring check-ups at higher institutions (data not shown).

Destination facilities

About 86% of all transfers were to government or government supported hospitals. This figure was highest in Himachal Pradesh (97%) and lowest in Andhra Pradesh where around 82% of women were transferred to government hospitals (table-4). With type of government facility, across the States, half of the IFTs were to district level secondary or tertiary hospitals while

For peer review only - http://bmjopen.bmj.com/site/about/guidelines.xhtml

non-IFTs were to sub-district Area or Civil hospitals or CHCs. Gujarat reported highest percentage of transfers to middle level facilities. Himachal Pradesh had more transfers to Civil-hospitals or higher (Table-4).

Distance and time travelled by 108 ambulance for pregnant women

The median distances travelled and time taken by '108' ambulances to transfer pregnant women are shown in Table-4. Ambulances travelled less than 4 kilometres to reach half of the pregnant women for IFTs across four States but up to 10 kilometres in Andhra Pradesh. For non-IFTs, ambulances travelled farther (between 9 and 12 kilometres) to reach half of the pregnant women. Among IFTs median distances from pick-up site to destination facility were between 16 kilometres in Chhattisgarh to 32 kilometres in Himachal Pradesh, but nearer in non-IFTs (between 8 and 32).

The median time taken for the ambulance to travel to the pick-up site for IFTs was between 10 minutes and 15 minutes for four states but longer (24 minutes) in Andhra Pradesh. The median time taken for travel from the referring to the destination facility was less than 40 minutes in Andhra Pradesh, Chhattisgarh and Gujarat but longer for Telangana (48 minutes) and Himachal Pradesh (83 minutes). For non-IFT, these times were about 30 minutes each, except for Himachal Pradesh where travel times were longer (Table-4).

For peer review only - http://bmjopen.bmj.com/site/about/guidelines.xhtml

Table 4: Characteristics of transfers for IFT and non-IFT by state (April 2013 to March 2014)

	Total		Andhra Pradesh		Chhattisgarh		Gujarat		Himachal Pradesh		Telangana	
	IFT (N=34,993)	Non-IFT (N=573,566)	IFT (N=14,574)	Non-IFT (N=132,800)	IFT (N=1,929)	Non-IFT (N=58,881)	IFT (N=6,562)	Non-IFT (N=263,509)	IFT (N=2,809)	Non-IFT (N=22,114)	IFT (N=9,119)	Non-IFT (N=96,262)
Obstetric emergency, %	8.4	4.4	5.4	3.1	18.0	3.9	10.4	5.5	21.0	7.8	5.8	3.2
Abnormal presentation	1.5	2.0	1.0	1.0	1.3	1.2	3.2	3.1	0.7	0.4	1.3	1.1
Bleeding in pregnancy	3.5	0.9	2.0	0.6	1.6	0.4	4.5	1.0	14.1	5.6	2.1	0.6
Eclampsia/ convulsion	0.7	0.1	0.6	0.2	0.7	0.1	0.6	0.1	1.1	0.1	0.8	0.2
Foetal loss	0.2	0.1	0.1	0.1	0.6	0.4	0.1	0.02	0.4	0.1	0.1	0.1
Medical condition	1.8	0.7	0.9	0.6	13.6	1.7	1.0	0.4	3.8	1.1	0.8	0.8
Previous caesarian	0.4	0.4	0.5	0.3	0.3	0.2	0.5	0.5	0.3	0.3	0.4	0.3
Precious pregnancy	0.3	0.3	0.2	0.2	0.1	0.1	0.5	0.4	0.5	0.2	0.3	0.2
Other emergency, %	3.7	1.8	4.1	2.8	8.7	1.2	2.3	1.0	0	0	4.2	3.5
Destination facility, %												
Government	82.0	79.6	82.1	81.7	87.6	96.8	70.2	72.0	96.0	97.6	85.3	84.1
District Secondary or tertiary hospital	53.2	19.8	54.2	29.4	64.3	28.5	30.1	7.3	86.9	46.7	55.7	32.0
Area / Civil hospital	19.8	13.4	18.1	21.1	0.6	0.8	31.5	8.3	7.7	27.3	21.3	22.5
Community Health Centre	7.6	34.6	8.3	19.7	20.5	57.4	7.4	43.6	0.9	20.4	6.3	16.2
Primary Health Centre	1.1	9.0	1.2	11.3	1.9	9.3	0.2	7.0	0.3	3.2	1.9	13.1
Sub-Health Centre	0	0.1	0	0	0.3	0.9	0	0	0	0	0	0
Information Missing	0.4	2.8	0.3	0	0	0	0.9	5.6	0	0	0.2	0.2
Government supported	3.4	7.7	0	0	0	0	16.9	15.9	0	0	0	0
Private	14.5	12.8	17.9	18.3	12.4	3.2	12.9	12.1	4.0	2.4	14.7	15.9
Total non-missing	(31,911) 100	(531,392) 100	(12,693) 12.9	(116,028) 100	(1,784) 100	(54,093) 100	(6,442) 100	(256,898) 100	(2,777) 100	(21,449) 100	(8,215) 100	(82,924) 100
Missing	(3082) 8.8	(42,174) 7.4	(1,881) 12.9	(16,772) 12.6	(145) 7.5	(4,788) 8.1	(120) 1.8	(6,611) 2.5	(32) 1.1	(665) 3.0	(904) 9.9	(13,338) 13.9
To different district	32.1	28.5	29.8	28.8	62.5	54.3	17.7	22.0	31.6	10.1	39.7	34.3
To different mandal	86.8	55.9	89.5	75.7	83.2	68.5	74.1	33.0	96.3	93.5	89.5	74.9
Distance call to pick-up site* Km Median (IQR)	2(1-14)	12(6-18)	10(3-18)	12(6-19)	4(1-6)	9(2-17)	1(1-10)	12(7-18)	1(1-1)	11(6-18)	3(1-15)	12(6-20)
Distance pick-up site to hospital,* km Median (IQR)	28(18-40)	15(8-23)	21(12-31)	17(10-26)	16(5-30)	8(3-15)	24(14-37)	14(8-21)	32(21-48)	12(6-20)	29(19-43)	17(10-28)
Time call to pick-up site,* minutes ; Median (IQR)	11(7-28)	25(16-37)	24(12-37)	27(17-40)	15(7-33)	27(17-39)	10(7-19)	24(16-34)	12(8-27)	35(22-56)	12 (7-29)	26(16-39)
Time pick-up site to hosp,* minutes ; Median(IQR)	49(32-70)	26(16-40)	37(23-55)	32(20-46)	40(26-64)	21(13-32)	40(26-60)	23(14-34)	83(57-116)	43(28-65)	48 (33-70)	31(20-48)
Time call to hospital,* minutes ; Median (IQR)	77(56-102)	63(46-84)	75(55-97)	72(54-93)	70(50-97)	56(39-77)	64(47-86)	56(42-74)	105(72-143)	80(54-121)	77 (57-103)	70(52-93)

IFT= Inter-facilty transfer; IQR=Interquartile range; *N varies- excludes deliveries by EMT that were not transported or missing value

For peer review only - http://bmjopen.bmj.com/site/about/guidelines.xhtml

Determinants of Inter-facility transfer

Overall, women with obstetric emergencies transported by 108 were roughly twice as likely to have an IFT as women with no obstetric emergency (crude OR 2.25, 95% CI 2.16-2.34) (Table-5). In the adjusted analysis (excluding Chhattisgarh), obstetric emergencies had 1.95 (95% CI, 1.83–2.06) times higher odds of having IFT compared to non-emergencies (Table-5). Women from urban areas were twice as likely to have IFTs as women in rural areas (AOR 2.34, 95% CI 2.26 – 2.40). There was no evidence of an independent effect of high-priority districts on IFT. Overall, there was evidence of a 'J' shaped trend with maternal age. IFTs were marginally more likely in women less than 25, with a trend of increasing odds in women aged 30 or more, compared to women aged 25-30 years (AOR 1.01, 1.04, 1, 1.21, 1.43 respectively for age groups $\leq$19, 20-24, 25-30, 30-34 and $\geq$35 years). Although the effects were small, women from backward castes (AOR 0.95, 95% C.I. 0.92-0.99), scheduled castes (AOR 0.94, 95% C.I. 0.90-0.98), and scheduled tribes (AOR 0.98, 95% C.I. 0.95-1.00) were less likely to have IFT than women from general castes, after adjustment. Women from below the poverty line (AOR 0.92, 0.88-0.96) were less likely to have IFTs compared to women above the poverty line.

The determinants of IFT had different pattern of effect across states as shown in table-5. In respect to individual state models, the association between obstetric emergencies and IFT was strongest for Chhattisgarh (AOR 5.32, 95% CI 4.70-6.02) followed by Himachal Pradesh (AOR 3.03, 95% CI 2.65-3.47).

For peer review only - http://bmjopen.bmj.com/site/about/guidelines.xhtml

For peer review only

Table-5 Determinants of IFT across states (Logistic regression analysis showing Odds ratio (95% C.I.))

	Total*	Andhra Pradesh	Chhattisgarh	Gujarat	Himachal Pradesh	Telangana
Bivariate logistic regression: OR (95% CI)						
Obstetric emergency	2.25 (2.16 – 2.34)	1.81 (1.67 – 1.95)	5.39 (4.76 – 6.09)	2.01 (1.85 – 2.17)	3.15 (2.84 – 3.49)	1.90 (1.73 – 2.09)
Urban area	2.29 (2.23 – 2.34)	1.87 (1.80 – 1.94)	1.09 (0.92 – 1.29)	4.93 (4.69 – 5.20)	6.34 (5.68 – 7.07)	1.68 (1.60 – 1.76)
High priority district	0.96 (0.94 – 0.98)	1.10 (1.06 – 1.14)	1.28 (1.13 – 1.44)	0.69 (0.65 – 0.73)	0.92 (0.83 – 1.01)	0.95 (0.90 – 1.00)
Age group (years)						
≤19	1.06 (1.00 – 1.13)	1.08 (0.98 – 1.18)	1.37 (1.13 – 1.66)	1.28 (1.08 – 1.51)	0.70 (0.57 – 0.86)	1.01 (0.88 – 1.14)
20 – 24	1.03 (1.00 - 1.05)	1.02 (0.98 – 1.07)	1.09 (0.97 – 1.21)	1.10 (1.04 – 1.16)	0.87 (0.79 – 0.95)	1.02 (0.97 – 1.07)
25 – 30	1	1	1	1	1	1
30 – 34	1.24 (1.18 - 1.30)	1.29 (1.17 – 1.42)	1.38 (1.16 – 1.65)	1.25 (1.15 – 1.36)	1.08 (0.93 – 1.25)	1.21 (1.08 – 1.35)
≥35	1.52 (1.41 – 1.64)	1.62 (1.41 – 1.86)	1.68 (1.31 – 2.15)	1.58 (1.39 – 1.80)	1.16 (0.93 – 1.46)	1.47 (1.23 – 1.76)
Social caste						
General caste	1	1	1	1	1	1
Backward caste	0.92 (0.89 – 0.96)	0.95 (0.90 – 1.01)	1.04 (0.68 – 1.58)	0.94 (0.87 – 1.02)	1.55 (1.36 – 1.77)	0.71 (0.65 – 0.77)
Scheduled caste	0.91 (0.87 – 0.94)	1.02 (0.97 – 1.09)	0.91 (0.58 – 1.44)	0.87 (0.79 – 0.96)	0.89 (0.80 – 0.99)	0.67 (0.61 – 0.73)
Scheduled tribe	0.90 (0.87 – 0.94)	1.18 (1.11 – 1.28)	0.83 (0.54 – 1.27)	0.75 (0.69 – 0.81)	1.10 (0.93 – 1.31)	0.71 (0.65 – 0.78)
Below poverty line	0.92 (0.88 – 0.96)	1.45 (1.04 – 2.01)	0.92 (0.22 – 3.80)	0.93 (0.89 – 0.98)	0.83 (0.76 – 0.90)	1.03 (0.75 – 1.41)
Multivariate logistic regression model: AOR (95% CI)**						
Obstetric emergency**	1.96 (1.83 – 2.06)	1.80 (1.65 – 1.95)	5.32 (4.70 – 6.02)	1.89 (1.74 – 2.05)	3.03 (2.65 – 3.47)	1.79 (1.61 – 1.98)
Urban area	2.35 (2.26 – 2.41)	1.86 (1.79 – 1.94)	1.09 (0.92 – 1.29)	4.75 (4.50 – 5.01)	5.76 (5.03 – 6.59)	1.64 (1.56 – 1.73)
High priority district	0.98 (0.95 – 1.00)	1.09 (1.05 – 1.13)	1.25 (1.11 – 1.41)	0.90 (0.85 – 0.96)	1.17 (1.03 – 1.33)	1.00 (0.95 – 1.06)
Age group (years)						
≤19	1.01 (0.94 – 1.06)	1.01 (0.92 – 1.11)	1.36 (1.12 – 1.65)	1.25 (1.06 – 1.49)	0.82 (0.65 – 1.05)	0.91 (0.80 – 1.05)
20 – 24	1.04 (0.98 – 1.06)	1.03 (0.99 – 1.08)	1.11 (1.00 – 1.24)	1.12 (1.06 – 1.18)	0.92 (0.82 – 1.03)	1.02 (0.97 – 1.07)
25 – 30	1	1	1	1	1	1
30 – 34	1.21 (1.14 – 1.27)	1.26 (1.14 – 1.39)	1.33 (1.11 – 1.59)	1.21 (1.11 – 1.32)	0.94 (0.77 – 1.14)	1.19 (1.07 – 1.34)
≥35	1.43 (1.35 – 1.56)	1.56 (1.36 – 1.80)	1.52 (1.19 – 1.95)	1.44 (1.27 – 1.64)	0.96 (0.72 – 1.30)	1.43 (1.19 – 1.71)
Social caste						
General caste	1	1	NA#	1	1	1
Backward caste	0.95 (0.92 – 0.99)	0.93 (0.87 – 0.99)	NA#	1.02 (0.94 – 1.10)	1.80 (1.55 – 2.10)	0.79 (0.71 – 0.87)

For peer review only - http://bmjopen.bmj.com/site/about/guidelines.xhtml

Scheduled caste	0.94 (0.90 – 0.98)	1.02 (0.95 – 1.08)	NA#	0.99 (0.90 – 1.09)	1.01 (0.90 – 1.14)	0.74 (0.61 – 0.73)
Scheduled tribe	0.98 (0.96 – 1.00)	1.22 (1.13 – 1.31)	NA#	0.94 (0.87 – 1.01)	1.31 (1.08 – 1.61)	0.82 (0.67 – 0.82)
Below poverty line	0.92 (0.88 – 0.96)	1.40 (1.00 – 1.96)	NA#	0.95 (0.90 – 1.00)	0.85 (0.77 – 0.95)	1.34 (0.95 – 1.88)
Pseudo R2	0.076	0.014	0.033	0.054	0.085	0.010

*adjusted for i.state and does not include State of Chhattisgarh; ** Adjusted for obstetric emergency, urban area, high priority district and age group, as appropriate; # for Chhattisgarh social caste and below poverty line were not included.

DISCUSSION

This is the first study assessing IFTs for pregnant women using the '108' ambulance service—the largest provider of the emergency medical services in India. We discuss findings with respect to patterns of use and the existing health system.

We estimated that '108' transferred around one fifth of all pregnancies and institutional deliveries in the five states. However, '108' service transported only 1% of all institutional deliveries in the population for IFTs. Only 1% of all institutional deliveries were transported by '108' for obstetric emergencies. The findings suggest that the '108' service is not a preferred choice for transport from lower-level facility to a higher-level facility, or for obstetric emergencies. Details about characteristics of obstetric emergencies transported by '108' ambulance services and discussion about coverage are published in another paper.[28]

The proportion of IFTs among all institutional deliveries will depend on the pattern of use of level of health care, referral practices and the availability of transport for between facility transfers. Roughly one-half of the non-IFTs went to peripheral birthing centres or basic EmOC centres in our study. A systematic review from India (including most studies from public health facilities) found that between 14% and 36% of all pregnancies were referred from nurse-run

For peer review only - http://bmjopen.bmj.com/site/about/guidelines.xhtml

delivery or basic EmOC centres, and a further 2% to 7% were referred from doctor- run basic EmOC centres for complications or emergencies.[13] Assuming the pattern of use of health facilities in our study and evidence from the review, we estimate that between 40,000 and 80,000 institutional deliveries who used '108' for non-IFTs may require further referral to higher facility. In addition, among the estimated 80% (2,300,000) institutional deliveries who went to their first facility by other means of transport, some women may be referred further. Thus the absolute numbers of pregnant women referred and requiring transport for IFT are likely to be large while '108' transports only about 35,000 pregnant women for IFT.

In our analysis, of all the transfers by '108' only 5.8% were IFTs—lowest 2.4% in Gujarat and highest 11.3% in Himachal Pradesh. The proportion of IFT was higher in States of Karnataka and Tamil Nadu (12.8% and 35.7%) among '108' users in 2013-14.[24] It appears that there is potential for increasing use of '108' for IFTs in the study States.

A study conducted in Andhra Pradesh in 2009, found that none of the pregnant women who used '108', was referred from a facility.[35] Some women did not prefer to wait for '108' if they perceived any emergency.[35] A maternal death review in Uttar Pradesh in 2010 found that only 5 of the 32 mothers who were transferred between facilities used an ambulance.[6] However, for other public transportation schemes, it was found that a high proportion (two-thirds) of all the inter-facility referrals in a study from Madhya Pradesh used Janani Express service (non-ambulance) while others used personal transport, taxis, autorikshaws or public transport.[10]

For peer review only - http://bmjopen.bmj.com/site/about/guidelines.xhtml

For peer review only

Although IFTs in our study were twice as likely to transport pregnant women who had any obstetric emergency compared to non-IFTs, there was a very large proportion of IFTs with no obstetric emergency or complication (92%). One of the ‘108’ doctors, during discussion to understand IFT processes, mentioned that on many occasions they were not convinced of the need for IFT. However on insistence of the referring staff, the ‘108’ doctor approved transport for IFT (personal communication). Often the referral was done because there was no doctor on duty or other resources were not available, as was also found in Madhya Pradesh.[10] These non-emergency IFTs will add unnecessary load at higher facilities and also make the ambulances unavailable for other emergencies.

In our study, IFTs had a higher overall proportion of women from disadvantaged social castes, below-the-poverty-line and urban areas compared to non-IFTs. Greater proportions of urban women deliver in health facilities,[30-32] and thus have a higher probability of early detection of complications and referral to a higher facility. Studies have also shown that higher proportions of women who use publically financed transportation schemes (‘108’/‘102’/Janani Express Yojana) belong to historically disadvantaged and backward social caste, below-the-poverty-line, are less educated and mostly from rural geographical areas compared to non-users.[21, 35-38]

The median time from call to ‘108’ and reaching the destination facility was similar for non-IFT, and IFT, and it ranged from 1 hour to 1.3 hour. A study of 57 maternal death reviews from Uttar

For peer review only - http://bmjopen.bmj.com/site/about/guidelines.xhtml

Pradesh found much longer times; the mean time taken to arrange transport and travel from home to facility was about 4 hours, and transport from one facility to another was about 10 hours.[6] Although the '108' service has a mandate to inform the destination facility before arrival to reduce delays in treatment, this is not practiced as the list of contact points is not provided to EMTs.[Source: Dr. GVR Rao] Few women were transferred to a district different from the originating district due to sparse distribution of referral facilities. These women also travelled longer distances. Continuity of care and monitoring of IFTs can be better if transfers are in same administrative unit i.e. district.

The '108' ambulances are stationed close to CHCs and should be readily available, but if the ambulance is on route for pick-up or drop-off of another client then the IFT client with complications will have to wait or arrange for another means of transportation.[21] The '108' service may consider prioritising IFTs by having dedicated fleet of ambulances for IFTs over transporting all non-IFTs by increasing the fleet of ambulances stationed closer to beneficiaries.[25] In some states, the under-utilised ambulances at the health facilities are being used through the '102' call centre along with '108', however the distribution of these into IFT and non-IFT use has not been well laid out.[21] In other States, '102' has new ambulances that focus on IFT or transfers back to home for pregnant women.[26] Since 2013, state of Assam has a dedicated fleet of 450 ambulances for IFTs operating through the '102' call system. Of these, over half of all IFTs were for pregnant women in 2015-16.[Source: GVK EMRI annual statistics] This stresses the need for dedicated ambulances to deal with emergency IFTs, equipped with advanced life and obstetric support facilities. Other services like Janani Express

For peer review only - http://bmjopen.bmj.com/site/about/guidelines.xhtml

yojana, do not provide clinical support during transfer and are found to take longer times in transfer of pregnant women.[21, 25]

The role of the '108' service in improving care at the referring and referral facility, and its overall impact on maternal morbidity and mortality reduction, could not be estimated from our analysis nor has been reported in other studies. Nonetheless, the '108' service is accepted as an important and successful intervention to improve patient transportation for obstetric, medical and other emergencies.[21, 24, 26, 35, 37] A study from five states, showed that '108' ambulances provide pre-hospital stabilising care to all pregnant women, and delivery and post-delivery care to those delivered at home or on the ambulance.[39] However, a study from Punjab found that about half of '108' ambulances did not meet required standards for basic life support.[37] Monitoring of the quality of en-route care and referral systems is integral for better outcomes and monitoring of any transport intervention.[16, 21, 40]

As we included representative states from all regions of India and included universal sample thus our results are generalizable to the all the pregnant women transported by '108' ambulance service in India. There are a few limitations to our analysis. Details of the type of emergency in the '108' database was based on the doctor's report or the diagnoses by the EMTs for IFTs, and only the EMTs for non-IFTs. This information is thus subject to inter-observer variability and differential reporting. However, we cannot estimate if this would have led to over-estimation or under-estimation of the effect estimate. A very few women who used '108' for antenatal care may have used it again for delivery care, and would have been counted

For peer review only - http://bmjopen.bmj.com/site/about/guidelines.xhtml

twice. The database did not have information if the women was referred after childbirth thus postpartum referrals could not be computed separately. Data for treatment given en-route, and doctors' notes on IFTs, were mostly not recorded in the '108' database and thus could not be studied. Details of the source hospital for IFTs were not available and thus details of transfers between type of facilities could not be assessed. There is a possibility that few calls for IFT for the women residing in rural areas were wrongly recorded, as they called from the health facilities in urban areas. This would have contributed to higher proportion of urban women among IFTs. The '108' service from Chhattisgarh was taken over by the '102' service since October 2013 thus use of '108' may be under-estimated for this state. We had large proportions of missing information on social and economic status from two states. We considered that the missingness was not at random and was not associated with outcome. There is evidence that in such situations a complete case analysis, as we reported, is associated with negligible bias compared to a multiple imputation approach.[41]

CONCLUSION

Of all the estimated institutional deliveries in India, only a very small proportion (6%) made use of the '108' ambulance for transfer between facilities. Among '108' users for IFTs around 92% did not have any complication or emergency. After adjusting for confounding factors, IFTs were more likely for women with obstetric emergencies, more than 30 years of age and from urban areas. Pregnant women from socially disadvantaged castes, below poverty line and from high priority districts were less likely to have IFTs. Utilisation of the '108' service and its determinants varied across states. Primary research is required to understand variation in

For peer review only - http://bmjopen.bmj.com/site/about/guidelines.xhtml

utilisation and to explore the potential of the '108' ambulance service to serve a higher proportion of women requiring IFTs, in particular those having obstetric emergencies. The '108' service would benefit by having a triage system to ensure that women with an obstetric emergency requiring an IFT are prioritised.

ACKNOWLEDGEMENTS

We acknowledge GVK-EMRI for sharing the data and allowing scientific study without any interference.

FUNDING

This work was supported by a Wellcome Trust Capacity Strengthening Strategic Award to the Public Health Foundation of India and a consortium of UK universities.

COMPETING INTERESTS

The authors have declared that no competing interests exist.

AUTHOR CONTRIBUTIONS

Conceived and designed the protocol: SS, PD, OC, GVR, GVSM. Contributed in analysis plan: SS, PD, OC, LO, GVSM. Analyzed and interpreted the data: SS. Contributed in preparation of manuscript: SS, PD, OC, LO, GVR, GVSM.

ETHICS APPROVAL

For peer review only - http://bmjopen.bmj.com/site/about/guidelines.xhtml

The research obtained ethics approval from ethics committees of both LSHTM and IIPH-Hyderabad. (LSHTM Ethics Ref: 9613; IIPHH Ethics Ref: IIPHH/TRC/IEC/009/2014)

DATA SHARING

The data were obtained and analysed within the premises of GVK-EMRI office under license for the current study. The data are not publically available however can be available upon reasonable request directly to GVK-EMRI.

REFERENCES

1. National Rural Health Mission (NRHM). All India executive summary: status as on 31st March 2013. New Delhi: NRHM, Government of India 2013.

2. Registrar General of India. SRS: Maternal Mortality Ratio Bulletin 2011-2013. New Delhi: Government of India 2014. http://www.censusindia.gov.in/vital_statistics/mmr_bulletin_2011-13.pdf (accessed 30 September 2016).

3. WHO. Trends in maternal mortality: 1990 to 2013. Estimates by WHO, UNICEF, UNFPA, The World Bank and the United Nations Population Division. Geneva: WHO 2014. http://apps.who.int/iris/bitstream/10665/112682/2/9789241507226_eng.pdf (accessed 30 September 2016).

4. UNICEF. Levels & Trends in Child Mortality: Report 2015. Estimates Developed by the UN Inter-agency Group for Child Mortality Estimation United Nations Levels. New York: UNICEF 2015.

For peer review only - http://bmjopen.bmj.com/site/about/guidelines.xhtml

http://www.unicef.org/publications/files/Child_Mortality_Report_2015_Web_9_Sept_15.pdf (accessed 30 September 2016).

5. WHO. The world health report 2005: Make every mother and child count. Geneva: WHO 2005. http://www.who.int/whr/2005/whr2005_en.pdf?ua=1 (accessed 30 September 2016).

6. Raj SS, Manthri S, Sahoo PK. Emergency referral transport for maternal complication: lessons from the community based maternal death audits in Unnao district, Uttar Pradesh, India. *International Journal of Health Policy and Management* 2015;4:99-106.

7. Singh S, Murthy GVS, Thippaiah A, Upadhyaya S, Krishna M, Shukla R, et al. Community based Maternal Death Review: Lessons learned from ten districts in Andhra Pradesh, India. *Maternal and Child Health Journal* 2015;19(7):1447-54.

8. WHO, UNFPA, UNICEF, AMDD. Monitoring emergency obstetric care: a handbook. Geneva: WHO 2009. http://apps.who.int/iris/bitstream/10665/44121/1/9789241547734_eng.pdf (accessed 30 September 2016).

9. Murraya SF, Pearson SC. Maternity referral systems in developing countries: Current knowledge and future research needs. *Social Science & Medicine* 2006;62:2205-15.

10. Chaturvedi S, Randive B, Diwan V, De Costa A. Quality of Obstetric Referral Services in India's JSY Cash Transfer Programme for Institutional Births: A Study from Madhya Pradesh Province. *PLoS ONE* 2014;9(5):e96773. doi:10.1371/journal.pone.0096773.

11. David KV, Pricilla RA, Venkatesan S, Rahman SP, G S YK, Vijayaselvi R. Outcomes of deliveries in a midwife-run labour room located at an urban health centre: results of a 5-year retrospective study. *National Medical Journal of India* 2012;25(6):323-6.

For peer review only - http://bmjopen.bmj.com/site/about/guidelines.xhtml

12. Iyengar K, Iyengar SD. Emergency obstetric care and referral: experience of two midwife-led health centres in rural Rajasthan, India. *Reproductive Health Matters* 2009;17(33):9-20.

13. Singh S, Doyle P, Campbell OM, Mathew M, Murthy GVS. Referrals between public sector health institutions for women with obstetric high-risk, complications, or emergencies in India –A systematic review. *PLoS ONE* 2016;11(8):e0159793. doi:10.1371/journal.pone.0159793

14. Raj SS, Manthri S, Sahoo PK. Emergency referral transport for maternal complication: lessons from the community based maternal death audits in Unnao district, Uttar Pradesh, India. *International Journal of Health Policy and Management* 2015;4:99-106.

15. Giovine A, Ostrowski C. Technical report on Improving Transportation and Referral for Maternal Health: Knowledge Gaps and Recommendations. Washington DC: 2010. https://www.wilsoncenter.org/sites/default/files/Transport%20Referral%20Meeting%20Technical%20Report.pdf (accessed 30 September 2016).

16. UNICEF. Operating Perinatal Referral Transport Services in Rural India: A toolkit. New Delhi: UNICEF 2010. https://www.mailman.columbia.edu/sites/default/files/pdf/unicef_2010_operating_perinatal_transport_services_in_rural_india_toolkit.pdf (accessed 30 September 2016).

17. Thaddeus S, Maine D. Too far to walk: maternal mortality in context. *Social science & medicine* 1994;38(8):1091-110.

18. Hussein J, Kanguru L, Astin M, Munjanja S. The effectiveness of emergency obstetric referral interventions in developing country settings: a systematic review. *Plos medicine* 2012;9(7):e1001264. doi:10.1371/journal.pmed.1001264.

For peer review only - http://bmjopen.bmj.com/site/about/guidelines.xhtml

19. Ministry of Health and Family Welfare. Directory of Innovations Implemented in the health Sector. New Delhi: MOHFW, Government of India 2008. http://planningcommission.nic.in/reports/genrep/health/Directory_of_innovations_march.pdf (accessed 30 September 2016).

20. Ministry of Health and Family Welfare. Operational Guidelines on Maternal and Newborn Health. New Delhi: MOHFW, Government of India 2010.

21. Sundararaman T, Chakraborty G, Nair A, Mokashi T, Ved R. Publicly Financed Emergency Response and Patient Transport Systems Under NRHM. New Delhi: Government of India 2012.

22. Ministry of Health and Family Welfare. Rural Health Statistics: 2014-15. New Delhi: Statistics Division, MOHFW, Government of India 2016. http://wcd.nic.in/sites/default/files/RHS_1.pdf (accessed 30 September 2016).

23. Kumutha J, Rao GVR, Sridhar BN, Vidyasagar D. The GVK EMRI maternal and neonatal transport system in India: A mega plan for a mammoth problem. *Seminars in Fetal & Neonatal Medicine* 2015;20(5).

24. Raj AX. Saving lives through rural ambulance services: Experiences from Karnataka and Tamil Nadu states, India. *Transport and Communications Bulletin for Asia and the Pacific*. 2014;84:52-66.

25. NHSRC. Emergency Medical servcie (EMS) in India: A concept paper. New Delhi: NRHM, MOHFW, Government of India 2012.

26. National Health Mission. ERS/Patient Transport Service: National Ambulance Service (NAS), MOHFW, Government of India 2015. http://nrhm.gov.in/nrhm-components/health-systems-strengthening/emri-patient-transport-service.html (accessed 30 September 2016).

For peer review only - http://bmjopen.bmj.com/site/about/guidelines.xhtml

27. GVK-EMRI. National footprints- Reaching unreachable Hyderabad, AP: GVK-EMRI 2015. http://www.emri.in/our-presence (accessed 30 September 2016).

28. Singh S, Doyle P, Campbell OM, Oakley L, Rao GVR, Murthy GVS. Transport of pregnant women and obstetric emergencies in India: An analysis of '108' ambulance service system data. BMC Pregnancy and Childbirth. 2016;16(299).

29. Ministry of Health and Family Welfare. Guidance note for implementation of RMNCH+A interventions in High Priority Districts. New Delhi: MOHFW, Government of India 2013.

30. IIPS. Himachal Pradesh: District level household and facility survey (2012-13) Mumbai: MOHFW, Government of India 2014. https://nrhm-mis.nic.in/DLHS4/Reports/Himachal%20Pradesh.pdf (accessed 30 September 2016).

31. IIPS. Telangana: District level household and facility survey (2012-13) Mumbai: MOHFW, Government of India 2014. https://nrhm-mis.nic.in/DLHS4/Reports/Telangana.pdf (accessed 30 September 2016).

32. IIPS. Andhra Pradesh: District level household and facility survey (2012-13) Mumbai: MOHFW, Government of India 2014. https://nrhm-mis.nic.in/DLHS4/Reports/Andhra%20Pradesh.pdf (accessed 30 September 2016).

33. NRHM. Formulas for calculating health and family planning indicators: NRHM, Government of India; 2008 [Available from: http://pbnrhm.org/formulas/health_indicators.pdf (accessed 10 February 2017).

34. Registrar General of India. Annual Health Survey 2012-13 Factsheet: Chhattisgarh. New Delhi: Ministry of Home affairs, Government of India 2014

For peer review only - http://bmjopen.bmj.com/site/about/guidelines.xhtml

http://www.censusindia.gov.in/vital_statistics/AHSBulletins/AHS_Factsheets_2012-13/FACTSHEET-Chhattisgarh.pdf (accessed 30 September 2016).

35. The Institute of Health Systems. A study of Emergency Response Services in Three Districts of Andhra Pradesh. New Delhi: NHSRC, Government of India 2011.

36. Prinja S, Aggarwal AK, Kaur M. Evaluation of Haryana Emergency Ambulance Services. New Delhi: PGIMER, Chandigarh & NHSRC, 2011.

37. Prinja S, Bahuguna P, Lakshmi PVM, Mokashi T, Aggarwal AK, Kaur M, et al. Evaluation of publicly financed and privately delivered model of emergency referral services for maternal and child health care in India. *Plos ONE*. 2014;9(10): e109911. doi: 10.1371/journal.pone.0109911.

38. Prinja S, Jeet G, Kaur M, Aggarwal AK, Manchanda N, Kumar R. Impact of referral transport system on institutional deliveries in Haryana, India. *Indian Journal of Medical Research* 2014;139:883-91.

39. Strehlow MC, Newberry JA, Bills CB, Min HE, Evensen AE, Leeman L, et al. Characteristics and outcomes of women using emergency medical services for thirdtrimester pregnancy-related problems in India: a prospective observational study. *BMJ Open* 2016;6:e011459. doi:10.1136/bmjopen-2016-011459.

40. Murray SF, Davis S, Phiri KR, Ahmed Y. Tools for monitoring the effectiveness of district maternity referral systems. *Health Policy and Planning*. 2001;16:353-61.

41. White IR, John B. Carlin. Bias and efficiency of multiple imputation compared with complete-case analysis for missing covariate values. *Statistics in medicine* 2010;29(28):2920-31.

For peer review only - http://bmjopen.bmj.com/site/about/guidelines.xhtml

STROBE 2007 (v4) Statement—Checklist of items that should be included in reports of *cross-sectional studies*

Section/Topic	Item #	Recommendation	Reported on page #
**Title and abstract**	1	(*a*) Indicate the study's design with a commonly used term in the title or the abstract	1,3,4
		(*b*) Provide in the abstract an informative and balanced summary of what was done and what was found	3,4
**Introduction**			
Background/rationale	2	Explain the scientific background and rationale for the investigation being reported	6-8
Objectives	3	State specific objectives, including any prespecified hypotheses	8
**Methods**			
Study design	4	Present key elements of study design early in the paper	9
Setting	5	Describe the setting, locations, and relevant dates, including periods of recruitment, exposure, follow-up, and data collection	9,10
Participants	6	(*a*) Give the eligibility criteria, and the sources and methods of selection of participants	9,10
Variables	7	Clearly define all outcomes, exposures, predictors, potential confounders, and effect modifiers. Give diagnostic criteria, if applicable	10-12
Data sources/ measurement	8*	For each variable of interest, give sources of data and details of methods of assessment (measurement). Describe comparability of assessment methods if there is more than one group	10
Bias	9	Describe any efforts to address potential sources of bias	10,12,27
Study size	10	Explain how the study size was arrived at	Universal sample
Quantitative variables	11	Explain how quantitative variables were handled in the analyses. If applicable, describe which groupings were chosen and why	11,12
Statistical methods	12	(*a*) Describe all statistical methods, including those used to control for confounding	11,12
		(*b*) Describe any methods used to examine subgroups and interactions	12
		(*c*) Explain how missing data were addressed	12
		(*d*) If applicable, describe analytical methods taking account of sampling strategy	NA
		(*e*) Describe any sensitivity analyses	12
**Results**			

For peer review only

Participants	13*	(a) Report numbers of individuals at each stage of study—eg numbers potentially eligible, examined for eligibility, confirmed eligible, included in the study, completing follow-up, and analysed	12,13
		(b) Give reasons for non-participation at each stage	13
		(c) Consider use of a flow diagram	NA
Descriptive data	14*	(a) Give characteristics of study participants (eg demographic, clinical, social) and information on exposures and potential confounders	15,16
		(b) Indicate number of participants with missing data for each variable of interest	16,17, Table-3
Outcome data	15*	Report numbers of outcome events or summary measures	14-16,
Main results	16	(*a*) Give unadjusted estimates and, if applicable, confounder-adjusted estimates and their precision (eg, 95% confidence interval). Make clear which confounders were adjusted for and why they were included	15-19
		(*b*) Report category boundaries when continuous variables were categorized	15-19
		(*c*) If relevant, consider translating estimates of relative risk into absolute risk for a meaningful time period	15-19
Other analyses	17	Report other analyses done—eg analyses of subgroups and interactions, and sensitivity analyses	20-22
**Discussion**			
Key results	18	Summarise key results with reference to study objectives	23-26
Limitations	19	Discuss limitations of the study, taking into account sources of potential bias or imprecision. Discuss both direction and magnitude of any potential bias	27
Interpretation	20	Give a cautious overall interpretation of results considering objectives, limitations, multiplicity of analyses, results from similar studies, and other relevant evidence	27
Generalisability	21	Discuss the generalisability (external validity) of the study results	27
**Other information**			
Funding	22	Give the source of funding and the role of the funders for the present study and, if applicable, for the original study on which the present article is based	29

*Give information separately for cases and controls in case-control studies and, if applicable, for exposed and unexposed groups in cohort and cross-sectional studies.

Note: An Explanation and Elaboration article discusses each checklist item and gives methodological background and published examples of transparent reporting. The STROBE checklist is best used in conjunction with this article (freely available on the Web sites of PLoS Medicine at http://www.plosmedicine.org/, Annals of Internal Medicine at http://www.annals.org/, and Epidemiology at http://www.epidem.com/). Information on the STROBE Initiative is available at www.strobe-statement.org.

For peer review only
